# Correlation of Polymer–drug Composition with Micelle Properties, Performance, and Cytotoxicity for the Oligoelectrolyte-mediated pH-triggered Release of Hydrophobic Drugs

**DOI:** 10.3390/polym18020247

**Published:** 2026-01-16

**Authors:** Md. Saddam Hussain, Riya Khetan, Hugo Albrecht, Marta Krasowska, Anton Blencowe

**Affiliations:** 1 Applied Chemistry and Translational Biomaterials (ACTB) Group, Centre for Pharmaceutical Innovation (CPI), School of Pharmacy and Biomedical Sciences, College of Health, Adelaide University, Adelaide, SA 5000, Australia; mdsaddam.hussain@adelaide.edu.au; 2 Department of Pharmacy, Faculty of Biological Sciences, Noakhali Science and Technology University, Noakhali 3814, Bangladesh; 3 Centre for Pharmaceutical Innovation (CPI), School of Pharmacy and Biomedical Sciences, College of Health, Adelaide University, Adelaide, SA 5000, Australia; riya.khetan@adelaide.edu.au (R.K.); hugo.albrecht@adelaide.edu.au (H.A.); 4 Surface Interactions and Soft Matter (SISM) Group, Future Industries Institute, Adelaide University, Mawson Lakes, SA 5095, Australia; marta.krasowska@adelaide.edu.au

**Keywords:** diblock copolymer, polymeric micelles, oligoelectrolyte, pH-responsive, triggered release, drug delivery

## Abstract

Polymeric micelles have the potential to improve the efficacy and safety of drug delivery by improving drug solubility, enhancing bioaccumulation and reducing off-target toxicity. Despite excellent safety profiles, a major limitation with polymeric micelles is their inability to rapidly release their payload once they have reached their target, leading to the inadequate delivery of therapeutic doses. To address this limitation, we have developed a novel strategy to impart pH-responsiveness in non-responsive micelles through the co-encapsulation of oligoelectrolytes with drugs. Herein, we investigate the influence of copolymer composition and drug identity in combination with oligoelectrolyte—oligo(2-vinyl pyridine) (OVP)—loading on pH-triggered drug release from micelles and their cytotoxicity. A library of OVP-loaded micelles was prepared using conventional and well-established non-responsive block copolymers. Dynamic light scattering (DLS) was used to monitor the changes in the micelles as a function of pH. Regardless of the copolymer composition, an abrupt decrease in the hydrodynamic diameter (*D_h_*) was observed as the pH was reduced due to OVP expulsion from the core, which was also confirmed by release studies. In general, co-encapsulation of OVP and model drugs (doxorubicin (DOX), gossypol (GP), paclitaxel (PX), and 7-ethyl-10-hydroxycamptothecin (SN38)) in the micelles provided good to excellent encapsulation efficiency percentage (EE%) values. In vitro studies revealed the pH triggered release of drugs from the OVP-loaded micelles regardless of the drug identity, which increased as the OVP loading increased. This general behaviour was observed in all cases, largely independent of the copolymer composition, albeit with subtle differences in the release profile for different drugs. Compared to their blank counterparts, the drug-loaded micelles displayed a slight increase in cytotoxicity against a panel of cancer cell lines, in a dose dependent manner. However, drug- and OVP-loaded micelles displayed a significant increase in cytotoxicity (up to 8-fold increase) that was independent of the copolymer composition. These results demonstrate the versatility of the oligoelectrolyte-mediated approach to furnish non-responsive micelles with a pH-trigger that allows the rapid release of drugs, regardless of the micelle composition or the drug identity.

## 1. Introduction

Over the last twenty years, many drug development programmes have trended towards more potent and lipophilic molecules, despite the greater clinical risk of poor selectivity and toxicological attrition [1]. A significant proportion of drugs in the developmental pipelines, as well as those already approved, are considered as ‘poorly soluble’ [2]. To improve the solubility, stability, and delivery of lipophilic drugs, various formulation approaches have been adopted, including surfactant solubilisation, co-solvency, cyclodextrin complexation, and nano/microemulsions [2]. In particular, lipid-based formulations have proven effective for the oral delivery of lipophilic drugs, with numerous examples already approved by regulatory agencies [3]. In addition, many liposomal formulations have been approved, or are undergoing clinical trials, for the intravenous and intramuscular delivery of lipophilic drugs that are not amenable to oral delivery [4]. However, liposomal formulations are prone to vesical destabilisation and drug leakage, and clearance by the reticuloendothelial system via opsonization and the accelerated blood clearance phenomenon, resulting in poor biodistribution and circulation times [5].

Polymeric micelles have been extensively studied as promising carriers for the intravenous delivery of lipophilic drugs [6,7]. Compared to liposomal formulations, polymeric micelles typically display higher lipophilic drug loadings, improved stability, reduced drug leakage, reduced clearance and enhanced circulation times, and smaller sizes promoting tissue penetration and bioaccumulation [8,9]. In addition, studies have shown that traditional, non-responsive polymeric micelles are generally well tolerated, with low toxicity and few adverse effects [10]. However, the inability to control drug release from non-responsive micelles (and liposomes) at the cellular or organelle level can lead to poor or imprecise drug delivery and the potential for off-target effects that can reduce therapeutic efficacy, resulting in toxicity to healthy tissues, and increasing the risk of drug resistance [11].

Alternatively, polymers can be readily engineered to afford stimuli-responsive micelles that respond to changes (e.g., temperature, pH, redox) in their surroundings to trigger drug release [12,13,14], overcoming limitations associated with poor or prolonged payload release from non-responsive systems [15,16]. To date, many bespoke stimuli-responsive micelle platforms have been developed for the delivery of specific drugs [15,16]. Despite these advantages, few polymeric micelle formulations have been clinically approved (e.g., Genexol-PM), although there are numerous formulations undergoing clinical trials [17]. Of note, the majority of these formulations are prepared from well-established, non-responsive block copolymers [6,18]. Some of the challenges with the clinical development of novel polymeric micelles include, long developmental periods, large scale production and reproducibility, and lengthy regulatory approval processes that are currently the same as for new drugs [18]. Thus, the translation of unique and bespoke stimuli-responsive polymeric micelles is individually more challenging as they are inherently more complex in their design and require more vigorous safety and efficacy testing. Perhaps, a more appealing and universal approach is to augment traditional, ‘generally regarded as safe’ (GRAS), non-responsive micelles with a common drug release trigger that could be broadly applied regardless of the drug identity or polymer composition (vide infra).

Of the various stimuli-responsive polymeric micelles that have been explored in the literature, pH-responsive micelles have emerged as versatile vectors that can exploit subtle changes in the pH of the extra- and intracellular environments to trigger delivery of their payloads [19]. pH-responsivity has been engineered into micelles via several approaches, which can be broadly classified according to the type of copolymer used and/or the mechanism that leads to drug release [14,20]. Acid-catalysed hydrolysis of certain functional groups or linkers has been commonly employed to switch the hydrophobicity of block copolymers [21,22] or release covalently conjugated drugs [23,24,25], respectively. An alternative approach that has also been extensively studied involves the use of copolymers whereby one block contains ionisable groups that become protonated at acidic pH, resulting in micelle disruption and the release of physically encapsulated drugs [26,27,28].

Micelles prepared from ionisable block copolymers typically display pH-dependent drug release, which has been shown to significantly enhance drug delivery in vitro and in vivo [29,30,31]. Nevertheless, the release of hydrophobic payloads from these types of micelles can be relatively slow (e.g., <30% at pH 5 over 5 h) [29,30,31] compared to endosomal trafficking, leading to their degradation in the lysosome. Furthermore, the encapsulation and release of payloads has also been reported to be highly dependent on drug identity and specific drug–polymer interactions [32]. pH-responsive micelles are generally larger in size, have greater positive charges and have higher critical micellization concentrations (CMCs) when compared to their non-responsive counterparts; these characteristics can lead to poor stability in biological environments, enhanced recognition and clearance by the immune system, and haemolysis due to undesirable interactions with components in the blood [33,34]. While mixed micelles, consisting of non-responsive copolymers and pH-responsive copolymers, have been studied to address these issues, they are still prone to many of these deficiencies [29,30,31,35].

Given the challenges of developing novel pH-responsive micelle delivery systems, particularly their apparent specificity to drug–polymer combinations, it is evident that a more universal approach towards pH-triggered drug release from micelles is required. Ideally, any such triggered payload delivery platforms should be broadly applicable to different GRAS polymeric micelles and drug classes while maintaining high encapsulation efficiencies, allowing rapid drug release at endosomal pH, with the potential for facilitating endosomal escape, and should not significantly influence the micelle size, charge or stability. In response to this challenge, we present the concept of oligoelectrolyte-mediated, pH-triggered released from non-responsive micelles. This strategy involves the co-encapsulation of polybasic oligoelectrolytes and lipophilic drugs in polymer micelles, with protonation of the oligoelectrolyte at pH values around its pk_a_ resulting in its ejection from the micelle core along with any co-encapsulated drugs. While our previous investigations demonstrated the proof-of-concept of oligoelectrolyte-mediated pH-triggered release using PEG-PCL micelles [36,37], the present study substantially expands this approach to compositionally diverse non-responsive copolymers and multiple drug classes with distinct physicochemical characteristics. This broader, systematic investigation demonstrates the versatility of this approach across different copolymer compositions and drug classes, maintaining high encapsulation efficiencies and achieving rapid release kinetics in response to pH changes, while preserving micelle charge, stability, and CMC.

## 2. Materials and Methods

### 2.1. Materials

α-Methoxy-ω-hydroxy PEG (molecular weight 10 kDa) was purchased from Advanced BioChemicals (Lawrenceville, GA, USA), and was dried at 120 °C under vacuum (0.01 mbar) for 60 min before use. PEG-PLA and PEG-PS were purchased from PolySciTech (West Lafayette, IN, USA) and Polymer Source (Montreal, QC, Canada), respectively. ε-Decalactone (≥99%), stannous octoate (Sn(Oct)_2_, ≥92.5–100.0%), 2-vinyl pyridine (VP, ≥97%), 1-dodecanthiol (≥98%), azobisisobutyronitrile (AIBN, 12 wt% in acetone), anhydrous toluene (≥99.8%), chloroform-d (CDCl_3_, 99.9% D), deuterium oxide (D_2_O, 99.9% D), deuterium chloride (DCl, 30 wt% in D_2_O, 99% D), sodium deuteroxide (NaOD, 99% D), anhydrous *N*,*N*-dimethylformamide (DMF, ≥99.8%), pyrene (≥99%), sodium acetate (NaOAc, ≥99.8%), PBS tablets, McCoy′s 5A medium (with L-glutamine and sodium bicarbonate), Roswell park memorial institute (RPMI)-1640 medium, foetal bovine serum (FBS), penicillin–streptomycin, Dulbecco′s phosphate-buffered saline (DPBS), dimethyl sulfoxide (DMSO, ≥99.5%), MTT (≥98%) and trypan blue solution were purchased from Merck (Rahway, NJ, USA). Analytical grade tetrahydrofuran (THF, ≥99.8%), dichloromethane (DCM, 99.8%), ethyl acetate (EA, ≥99.9%), n-hexane (≥95%), dioxane (≥99.8%), methanol (≥99.9%), acetone (≥99.8%), acetic acid (≥99.8%), chloroform (99.9%), acetonitrile (ACN, ≥99.9%) and diethyl ether (DEE, ≥99.9%) were purchased from ChemSupply (Gillman, SA, Australia). DOX (≥98%), PX (≥99%), SN38 (≥99.8%) and GP (≥98%) were purchased from the Cayman Chemical Company (Ann Arbor, MI, USA), Selleck Chemicals (Houston, TX, USA), MedChemExpress (Monmouth Junction, NJ, USA) and Shaanxi Ciyuan biotech (Xi’an, China), respectively. PBS solutions (10 mM, pH 7.4) were prepared according to the manufacturer’s guidelines. NaOAc buffer (10 mM, pH 4.5) was prepared in high-purity water. High-purity water was obtained from a Sartorius Arium^®^ Pro Ultrapure Water Systems (Göttingen, Germany) UV-T-TOC and had a resistivity of ≥ 18.2 MΩ.cm. Ultrahigh purity argon (99.999%) was purchased from the British Oxygen Company (BOC) (North Ryde, NSW, Australia) and passed through Drierite (W. A Hammond, Xenia, OH, USA) prior to use. Prostate cancer (PC-3) and ovarian cancer OvCa (SKOV-3) cell lines were sourced from Sigma (St. Louis, MO, USA) and ovarian cancer (ES-2) cells were purchased from in vitro technologies (Noble Park North, VIC, Australia). Trypsin-EDTA (0.25%)-phenol red solution, 0.22 µm nylon syringe filters and Nunc™ MicroWell™ 96-Well, Nunclon Delta-treated, flat-bottom microplates were purchased from Thermo Fisher Scientific (Waltham, MA, USA).

### 2.2. Instrumentation

^1^H NMR spectroscopy was carried out at 23 ± 1 °C using Bruker NMR AVANCE III HD 500 or 600 spectrometers (Bruker BioSpin, Billerica, MA, USA) operating at 500 and 600 MHz, respectively. CDCl_3_ was employed as the solvent for polymer analysis, with the residual solvent peak (CHCl_3_; *δ_H_* 7.26 ppm) used as an internal reference. For NMR spectroscopy investigations, micelles were prepared in 10 mM PBS-d at a concentration of 0.5 mg/mL, and the pH was adjusted with DCl or NaOD before analysis.

pH measurements were carried out using an Oakton pH 700 benchtop pH metre (Charleston, SC, USA) with an Oakton All-in-one pH/ATC Electrode, Single Junction, 12 mm probe. In experiments involving D_2_O, the same H_2_O-calibrated pH metre was used, providing pD values that were then converted to pH using the formula reported by Krȩżel and Bal: pH = 0.929pD + 0.41 [38].

To determine the molecular weight characteristics, *M_n_*, weight-average molecular weight (*M_W_*), and dispersity (*Đ*) of the polymers, GPC was performed at 40 °C using a Prominence liquid chromatography system (Shimadzu, Nakagyo-ku, Kyoto, Japan) equipped with a Shimadzu RID-10A refractive index detector and two Shimadzu Shim-pack columns in series (GPC-8025D and GPC-805D). The mobile phase used was THF at a flow rate of 1 mL/min. Samples were prepared at a concentration of ~10 mg/mL and filtered through 0.22 μM nylon syringe filters before analysis. The molecular weight characteristics were determined by comparing them to a conventional column calibration with narrow *Đ* PEG or PS standards (Polymer Standards Service GmbH, Mainz, Germany).

DSC was performed on a TA Instruments Discovery Differential Scanning Calorimeter (New Castle, DE, USA). The polymers were accurately weighed in aluminium pans using an Aczet CM 19 microbalance (Maharashtra, Mumbai, India). Subsequently, the pans were sealed with lids using the Tzero DSC sample encapsulation press. To prevent oxidative degradation, all analyses were conducted under a nitrogen flow with a cell purge rate of 50 mL/min.

DLS was performed on a Malvern Zetasizer NANO ZS (Worcestershire, UK) equipped with a 4 mW He–Ne laser source operating at a wavelength of 633 nm. UV-vis spectrophotometry was conducted at 21 ± 1 °C on Thermo Fisher Scientific Evolution 201/220 UV–visible spectrophotometer (Waltham, MA, USA) using low volume (1 mL, 10 mm) quartz cuvettes (Starna Pty Ltd., Melbourne VIC, Australia). CMCs were determined at 21 ± 1 °C using a Shimadzu RF-6000 spectrofluorometer (Nakagyo-ku, Kyoto, Japan).

HPLC was conducted on a Prominence liquid chromatography system (Shimadzu, Nakagyo-ku, Kyoto, Japan) equipped with a quaternary pump (LC-20AD), autosampler (SIL-20A HT), photodiode array detector (SPD-M20A), and fluorescence detector (RF-20A). A μBondapak TM C18 column (3.9 × 300 mm, 10 μm) was used for all analyses. For the quantification of PX and OVP, separate solvent gradient methods were employed at a flow rate of 1 mL/min and 50 μL injection volume, using UV detection at *λ_max_* = 227 and 264 nm, respectively. A 19 min ramp gradient method was used for PX: 40% ACN + 60% water for 9 min (1 mL/min); 80% ACN + 20% water for rest 5 min (1 mL/min); 40% ACN + 60% water for rest 5 min (1 mL/min). A 15 min ramp gradient method was used for OVP: 80% ACN + 20% water for 5 min (1 mL/min); 95% ACN + 5% water for rest 5 min; 80% ACN + 20% water for 5 min (1 mL/min).

In vitro cell studies were performed in a Safe 2020 Class II biosafety cabinet (Thermo Fisher Scientific, Waltham, MA, USA). Cell lines were cultured at 37 °C in a Heracell 150i CO_2_ Incubator (Thermo Fisher Scientific, Waltham, MA, USA). Cell counting was conducted using Neubauer Haemocytometer Counting Chambers and an Olympus CK 2 phase contrast microscope (Hampton, NH, USA). Optical density (OD) measurements were recorded using an EnSpire Multimode Plate Reader (PerkinElmer, Waltham, MA, USA).

### 2.3. Procedures

#### 2.3.1. Characterisation of Diblock Copolymers and Oligo(2-Vinyl Pyridine)

Synthesis and characterisation of the PEG_10_PDL_10_, PEG_14_PS_12_ and PEG_10_PLA_1_**_0_** copolymers and OVP was previously reported [36]. The PEG_5_PS_5_ copolymer was characterised via ^1^H NMR spectroscopy and GPC (Supplementary Materials (SM), Figure S1). ^1^H NMR (500 MHz, CDCl_3_) *δ_H_* 1.12–1.83 (PS repeat unit (RU), –C**H**_2_(C**H**)C**H**_2_–), 3.39 (PEG end group, –OC**H**_3_), 3.63 (PEG RU, –OC**H**_2_C**H**_2_–), 7.05–6.25 (PS RU, –C_6_**H_5_**) ppm; *M_n,NMR_* = 10.7 kDa. GPC (relative to PEG standards): *M_w,GPC_* = 12.9 kDa, *M_n,GPC_* = 11.5 kDa, *Đ* = 1.12.

For DSC analysis, the PEG_10_PDL_10_, PEG_5_PS_5_, PEG_14_PS_12,_ and PEG_10_PLA_10_ copolymers were heated from 25 to 160 °C (first heating cycle), cooled from 160 to −80 °C (first cooling cycle), and heated from −80 to 160 °C (second heating cycle) at a ramp rate of 10 °C/min and 5 °C/min (for PEG_10_PLA_10_ only) (SM, Figures S1–S4 and Table S1). *T_m_*, *T_cryst_* and *T_g_* and their associated enthalpic changes were determined by linearly integrating the thermal transitions using the Universal Analysis software package (v4.5A) (TA Instruments, New Castle, DE, USA).

#### 2.3.2. Preparation and Characterisation of Copolymeric Micelle Solutions

Micelles were prepared via the solvent evaporation as previously described [36,37]. Detailed procedures for the preparation and characterisation of blank, OVP-loaded and drug-loaded copolymeric micelles are provided in the Supplementary Materials. For characterisation of the blank and OVP-loaded micelles via DLS, ^1^H NMR spectroscopy, and UV-vis spectrophotometry, micelles were prepared in PBS-d solutions and the pH was adjusted with NaOD or DCl solutions. Preliminary experiments revealed negligible difference in the *D_h_* of micelles prepared in H_2_O or D_2_O solutions. For determination of drug and OVP (co)encapsulation and release, micelles were prepared in PBS solutions. Release studies were conducted using dialysis membranes and a switch in the receiving solution from pH 7.4 to 4.5. The concentration of drugs and OVP in the receiving solution was monitored using a combination of UV-vis spectrophotometry and HPLC.

#### 2.3.3. Cell Studies

Prostate (PC-3) and ovarian cancer (SKOV-3 and ES-2) cell lines were used to evaluate the cytotoxicity of blank and OVP-loaded copolymeric micelles with model drugs via an MTT assay, as previously described [36,37]. Detailed procedures for cell studies with blank, OVP-loaded, and drug-loaded copolymeric micelles are provided in the Supplementary Materials.

## 3. Results and Discussions

### 3.1. Influence of Copolymer Composition on Oligoelectrolyte Encapsulation and pH-Triggered Release

The influence of oligoelectrolyte loading and copolymer composition on the oligoelectrolyte-mediated, pH-triggered release of therapeutics from micelles was studied using a library of non-responsive block copolymers including poly[(ethylene glycol)-*b*-(styrene)] (PEG_x_PS_y_), poly[(ethylene glycol)-*b*-(ε-decalactone)] (PEG_x_PDL_y_), and poly[(ethylene glycol)-*b*-(D,L-lactic acid)] (PEG_x_PLA_y_) (Table 1), whereby x and y denote the number-average molecular weight (*M_n_*) of the blocks as determined by proton nuclear magnetic resonance (^1^H NMR) spectroscopy. The weight fraction of the hydrophobic block (*f*_HB_) was fixed at ~0.5 to allow the influence of polymer composition to be studied independently of the hydrophilic–lipophilic balance (HLB). Additionally, two different molecular weight PEG_x_PS_y_ copolymers (PEG_5_PS_5_ and PEG_14_PS_12_) were used to investigate the effect of overall molecular weight. The various copolymer compositions were selected as they show good biocompatibility, have been commonly employed for drug encapsulation and delivery [39,40,41,42], and represent hydrophobic blocks (HBs) with very different compositions, physical characteristics and morphologies. OVP was used as the oligoelectrolyte due to its pK_a_ of ~5, allowing it to respond to the late endosomal environment while effectively being neutral and hydrophobic at physiological pH values [36].

The thermal properties of the copolymers were investigated by differential scanning calorimetry (DSC) with the primary aim of identifying the glass transition temperature (*T_g_*) of the HB, which may play a role in the encapsulation and release of cargo in the copolymeric micelles (SM, Figures S1–S4 and Table S1). For the PEG_10_PDL_10_ copolymer, a *T_g_* was observed at −55 °C, which was assigned to the PDL block, as previously reported in the literature [40]. In contrast, the other copolymers did not display obvious *T_g_* transitions, but it can be reasoned based on prior literature that the PLA and PS block would be expected to have *T_g_* values around 60 and 100 °C, respectively [43,44]. Other features that were observed in the thermograms of the copolymers were related to the melting and crystallisation of the PEG block. All copolymers displayed melting peak maxima (*T_m_*) for the PEG block between 50 and 60 °C, consistent with previous literature [45,46]. However, the PEG block temperature of crystallisation (*T_cryst_*) varied significantly depending on the HB and molecular weight. Despite the shorter and longer PEG blocks of the PEG_5_PS_5_ and PEG_14_PS_12_ copolymers presenting with *T_cryst_* of 11 and 38 °C, respectively, the PEG degree of crystallinity (*X_c_*) was constant at 38%. For the PEG_10_PDL_10_ copolymer, the *T_cryst_* and *X_c_* were 41 °C and 53%, respectively. The thermal behaviour of the PEG_10_PLA_10_ copolymer was distinct from the other copolymers, with the heating cycle revealing an exothermic cold crystallisation peak [47,48], and the occurrence of a PEG *T_cryst_* at 20 °C (*X_c_* = 38%) being highly dependent on the cooling rate. These results are consistent with the more crystalline PS and PLA blocks having a larger influence on the PEG crystallinity, as compared to the amorphous PDL block [49].

The solvent evaporation approach was employed to prepare blank and OVP-loaded (0.1, 0.2, and 0.3 mg/mg of copolymer) micelles in phosphate-buffered saline (PBS; 10 mM, pH 7.4). The OVP-loaded micelles were designated PEG_x_HB_y_OVP_z_, whereby *z* refers to the OVP loading in mg/mg of copolymer. Upon visual inspection, all micelle solutions appeared completely transparent without any visible precipitation (SM, Figure S5), indicating the successful formation of micelles and the encapsulation of OVP. Furthermore, no visual changes were observed in the solutions upon storage, indicating that OVP encapsulation does not negatively impact the colloidal stability of the micelles, as previously reported for PEG-PCL micelles [36]. In contrast, control experiments with OVP in the absence of the copolymers resulted in immediate precipitation of the OVP. The EE% and loading (wt%) of OVP was predominately dependent on the OVP loading rather than the composition of the polymer (Table 2 and SM, Table S2). PEG_14_PS_12_OVP_z_ displayed the highest OVP EE% values (≥92%) regardless of the OVP loading, while its lower molecular weight counterpart displayed some of the lowest at each OVP loading, which was rationalised to be due to the smaller hydrophobic core resulting in a reduced encapsulation capacity (Table 2). Overall, the OVP loadings were consistent for all micelles, with values of ~9, 15, and 20 wt% resulting from initial OVP loadings of 0.1, 0.2, and 0.3 mg/mg of copolymer, respectively.

The critical micelle concentration (CMC) of the copolymers (Table 2 and SM, Table S3) was determined using pyrene as a probe in conjunction with ratiometric fluorescent spectroscopy [50] (SM, Figures S6–S9). The CMC values of the PEG_x_PS_y_ copolymers were determined to range between 0.6–0.8 mg/L. For the PEG_10_PDL_10_ and PEG_10_PLA_10_ copolymers the CMCs ranged between 0.5–0.7 mg/L. These CMC values are in agreement with previously reported values for PEG-*b*-PS [51], PEG-*b*-PDL [41], and PEG-*b*-PLA [52] copolymers of similar molecular weights. Importantly, the encapsulation of OVP had a negligible effect on the CMC values of the copolymers, implying that the micelles could be rendered pH-responsive without negatively impacting their stability. In contrast, previous attempts to render traditional micelles pH-sensitive through the formation of mixed micelles with pH-responsive block copolymers have typically resulted in significant increases in the CMC by orders of magnitude [29,31]. The zeta potential (ζ) values of the blank polymeric micelles were very slightly negative and trended upwards upon the encapsulation of OVP (Table 2), although the magnitude of the values is low and typically the surface charge of the micelles would be considered as near neutral. Similar, near-neutral surface charges are also characteristic of clinically approved PEGylated micelles, which demonstrate excellent colloidal stability and prolonged circulation [53,54].

**Figure 1:**
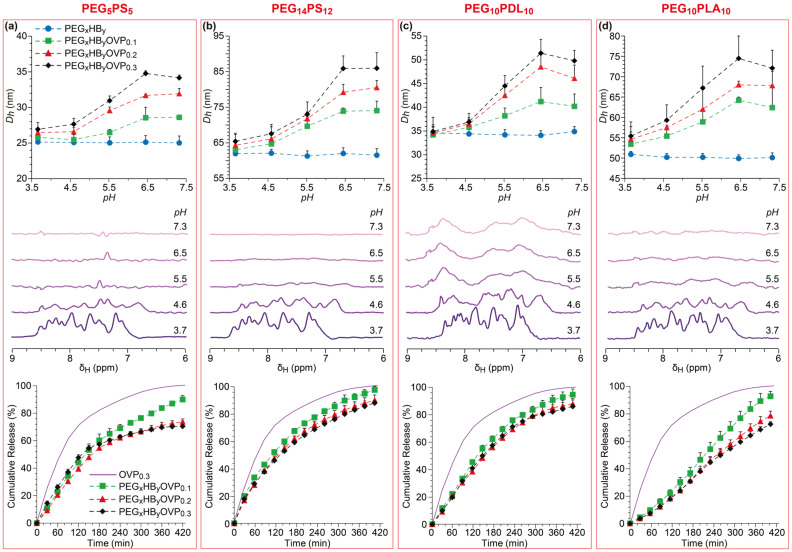
Hydrodynamic diameter (*D_h_*) as a function of pH (top) for the blank and OVP-loaded (0.1, 0.2, and 0.3 mg/mg of copolymer) (**a**) PEG_5_PS_5_, (**b**) PEG_14_PS_12_, (**c**) PEG_10_PDL_10_, and (**d**) PEG_10_PLA_10_ micelles as determined from DLS; data are shown as the number-average size + SD (*n* = 3). ^1^H NMR spectra (600 MHz, 25 ± 1 °C) as a function of pH (middle) showing the pyridyl proton resonance region for OVP-loaded (0.3 mg/mg of copolymer) micelles. In vitro OVP release (bottom) across a dialysis membrane for free OVP (purple line) and from OVP-loaded (0.1, 0.2, and 0.3 mg/mg of copolymer) micelles in 10 mM acetate buffer (pH 4.5, 22 ± 1 °C); data are shown as the average cumulative release (%) + SD (*n* = 3). Only symbols represent the experimental data; the lines are guides for the eyes.

A combination of DLS, NMR spectroscopy, and UV-vis spectrophotometry were used to investigate the pH-dependent changes in the blank and OVP-loaded micelle size and OVP ionisation. To allow these experiments to be conducted on the same micelle samples, micelles were prepared in 10 mM PBS in D_2_O (PBS-d; pH 7.3) and subsequently pH adjusted (SM, Tables S4–S7). The pH-dependent changes in the particle size distributions (PSDs) and polydispersity index (PDI) values of the blank and OVP-loaded micelles were measured by DLS. In all cases, narrow monomodal number, volume, and intensity PSDs (SM, Figures S10–S13) with low PDI values (Table 2 and SM, Table S8) were observed for all micelles at all pH values, providing comprehensive evidence of sample uniformity. For the blank micelles, there was negligible change in the hydrodynamic diameter (*D_h_*) or PDI, regardless of the pH (Figure 1 and SM, Table S9). Unsurprisingly, an increase in the copolymer molecular weight going from blank PEG_5_PS_5_ to PEG_14_PS_12_ micelles led to an increase in the *D_h_* from ~25 (PDI ~ 0.14) to ~62 nm (PDI ~ 0.16). The *D_h_* values of the blank PEG_10_PDL_10_ and PEG_10_PLA_10_ micelles were ~35 (PDI ~ 0.14) and ~50 nm (PDI ~ 0.16), respectively. The relatively small *D_h_* of the PEG_10_PDL_10_ micelles (*cf.* PEG_14_PS_12_ and PEG_10_PLA_10_ micelles) likely arises from the low *T_g_* and amorphousness of the HB, allowing for a more compact core with reduced free volume [49,55].

Encapsulation of OVP at pH 7.4 led to a loading-dependent increase in the size of the micelles, with the *D_h_* increasing by ~15% (*cf.* blank micelles) each time the OVP loading was increased, regardless of the copolymer composition (Figure 1). This increase in size was also accompanied by an OVP loading-dependent increase in the PDI values (SM, Table S8). Considering the OVP EE%, the theoretical hydrodynamic diameter (*D_h,theo_*) of the micelles was calculated at 100% loading (presuming that the OVP packing in the core remains consist regardless of the loading and the volume scales linearly with loading), which revealed a proportional relationship between the *D_h_* and OVP loading (Table 2 and SM, Figure S14 and Table S9). It should be noted that the *D_h,theo_* values were extrapolated by assuming that the micelles are 100% loaded with OVP and are extensively used for modelling purposes to visualise loading-dependent trends, whereas actual quantitative analyses and all experimental interpretations used the measured EE% values. Interestingly, this linear relationship extended to the blank micelles for PEG_5_PS_5_ and PEG_10_PDL_10_, while a larger initial increase was observed in going from the blank micelles to PEG_14_PS_12_OVP_0.1_ and PEG_10_PLA_10_OVP_0.1_ micelles, which is possibly related to the higher crystallinity of the HB in the micelle core and the initial disruption of the polymer chain packing to accommodate the OVP.

A decrease in the pH to 6.5 resulted in negligible changes in the *D_h_* of the OVP-loaded micelles, while further decreases resulted in a significant reduction, with the *D_h_* at pH 4.5 approximating that of the blank micelles, with the exception of the PEG_10_PLA_10_OVP_z_ micelles (Figure 1 and SM, Figures S10–S13). These results are consistent with ionisation of the OVP and its release from the core of the micelles, leading to core collapse and shrinkage, and resulting in micelles with similar *D_h_* and PDI values as their blank counterparts (vide infra). For the PEG_10_PLA_10_OVP_z_ micelles, the release of OVP and decrease in the size of the micelles was more gradual and only approximated that of the blank micelles at pH 3.5. This distinct behaviour suggests that there may be stronger interactions between the protonated OVP and the PLA block (*cf.* with other copolymer compositions) that leads to a delayed release of the OVP, and a higher fraction of ionisation and electrostatic repulsion is required to overcome these interactions (vide infra). While further experiments are required to confirm the exact nature of these interactions, ion–dipole forces between the carbonyl and pyridinium groups may be responsible.

^1^H NMR spectra of the blank and OVP-loaded PEG_5_PS_5_, PEG_14_PS_12,_ and PEG_10_PLA_10_ micelles at pH 7.3 revealed resonances corresponding to the methylene protons of the PEG backbone (*δ_H_* 3.65 ppm), while resonances corresponding to the HBs or OVP were absent (Figure 1 and SM, Figures S15–S18). This is in contrast with previous analyses of PEG-*b*-PCL micelles [36] and the PEG_10_PDL_10_ micelles at pH 7.3, whereby characteristic resonances of the PEG and polylactone blocks, and OVP pyridyl protons (*δ_H_* 6–8.5 ppm) can be observed. These observations are consistent with the *T_g_* of the HBs. While PS and PLA both have *T_g_* values well above ambient temperature, the low *T_g_* of PDL provides increased rotational mobility of protons in the hydrophobic core and long enough relaxation times for detection of the core components [56]. It is also probable that the amorphousness of the PEG_10_PDL_10_ micelle core results in a semi-hydrated core or boundary layer that further enhances proton mobility [56,57], while the more crystalline core of the PEG_5_PS_5_, PEG_14_PS_12_ and PEG_10_PLA_10_ micelles favours the exclusion of water. At pH 5.5, weak pyridyl proton resonances can be observed for the PEG_14_PS_12_ and PEG_10_PLA_10_ micelles, implying some release of OVP from the core (Figure 1). Interestingly, pyridyl proton resonances remained absent from the PEG_5_PS_5_ micelle spectrum at pH 5.5, suggesting that OVP is retained in the micelle. When the pH was decreased further, the intensity of the pyridyl proton resonances increased for all the copolymeric micelles (as quantified in the OVP/PEG signal integral ratio; SI, Figures S15–S18), and there were changes in the chemical shifts (Figure 1), which were consistent with OVP ionisation and its release from the core.

Ionisation of the OVP was further confirmed by UV-vis spectrophotometry by following changes in the pH-sensitive absorbance at *λ*_264_ (π–π* transition) [58,59] (SM, Figures S19–S22). Regardless of the copolymer composition or OVP loading, an increase in the OVP absorbance was observed below pH 6.5, with the absorbance from PEG_x_HB_y_OVP_0.3_ micelle solutions approximating those of an equivalent amount of free OVP. An exception to this trend was the PEG_5_PS_5_OVP_0.3_ micelle solutions, which had lower than expected absorbance values consistent with incomplete OVP ionisation or release (SM, Figure S19), and hinting at unique structural differences in the micelle core (*cf*. PEG_14_PS_12_ micelles).

Release of OVP from the micelles (SM, Table S10) was studied using an in vitro dialysis assay, whereby OVP-loaded micelles in PBS (pH 7.4) were placed in a dialysis membrane (molecular weight cut-off (MWCO) 100 kDa) that was suspended in either PBS at pH 7.4 (no release was observed at this pH) or acetate buffer at pH 4.5 (Figure 1 and SM, Figure S23). Regardless of the OVP loading, rapid release was observed for the PEG_14_PS_12_OVP_z_ and PEG_10_PDL_10_OVP_z_ micelles, with the release profiles approximating those of control experiments with OVP in the absence of micelles. The release profiles for free OVP represents the diffusion limit for OVP across the membrane, which is dependent on the concentration gradient, as confirmed by a strong correlation to first-order kinetics (SM, Table S11). Thus, the similarity in the OVP release profiles for the control and PEG_14_PS_12_OVP_z_ and PEG_10_PDL_10_OVP_z_ micelles implies that the majority of the encapsulated OVP is instantaneously ejected from the core following buffer exchange (Figure 1). This was further supported by modelling of the OVP release profiles from the PEG_14_PS_12_OVP_z_ and PEG_10_PDL_10_OVP_z_ micelles, which revealed excellent fits with first-order kinetics (SM, Table S11). While there are several goodness-of-fit statistical tests that can be used to evaluate the model fitting of the release profile, the model selection criterion (MSC)—a modified reciprocal form of the Akaike information criterion (AIC), which is normalised to be independent of the scaling of the data points—was used primarily in this study. Similar behaviour was also observed for the PEG_5_PS_5_OVP_z_ micelles; however, for higher OVP loadings the release profiles plateaued at ~65%, implying that the OVP is not completely released and remains associated with the micelles, consistent with the UV-vis spectrophotometry results. Modelling of the OVP release profile from the PEG_5_PS_5_OVP_0.3_ micelles revealed reasonable fits with Higuchi and Korsmeyer–Peppas models, implying that the OVP release was limited by either water ingress into the micelle core, OVP dissolution or OVP–core interactions (SM, Table S11). These observations are similar to the behaviour of PEG_4_PCL_4_ micelles [37] and imply heterogeneous distribution of OVP within the PEG_5_PS_5_ micelles.

OVP release from the PEG_10_PLA_10_OVP_z_ micelles displayed distinctly different behaviour, with near linear release profiles suggesting that OVP is released more gradually from the micelles, as observed from DLS measurements (Figure 1). Modelling revealed good correlations with Korsmeyer–Peppas kinetics, with exponents close to 1 indicating zero-order release resulting from polymer degradation or relaxation (SM, Table S11), although polymer degradation is expected to be extremely slow relative to the time over which these release studies were conducted [60,61].

### 3.2. Influence of Copolymer Composition and OVP Loading on Drug Co-Encapsulation

The influence of copolymer composition and OVP loading on drug co-encapsulation and release from micelles (SM, Table S12) was investigated with respect to different hydrophobic drugs. The inhibitors of DNA replication/repair, DOX (anthracycline derivative) and SN38 (camptothecin derivative), dehydrogenase inhibitor GP (polyphenolic derivative) and mitosis inhibitor PX (taxoid derivative) were selected to represent a broad range of drug classes with different physiochemical properties. In addition, two different drug loadings (0.1 and 0.2 mg/mg of copolymer) were adopted for encapsulation studies to assess the interplay between drug and OVP co-encapsulation. The drug-loaded micelles were designated as PEG_x_HB_y_OVP_z_D_#_, whereby D refers to the drug acronym and # refers to the drug loading in mg/mg of copolymer. Except for PEG_5_PS_5_ micelles with SN38, all micelle preparations formed homogeneous solutions with no obvious precipitation, regardless of the OVP or drug loading, implying successful co-encapsulation. Due to the poor encapsulation of SN38 in PEG_5_PS_5_ micelles in the absence of OVP (EE% ~12–17%) (SM, Table S13), no attempts were made to co-encapsulate the drug with OVP. Similar trends in SN38 EE% with HB length have also been reported by Duan et al. for a series of chitosan-*g*-PCL copolymeric micelles, whereby shorter PCL segments resulted in lower EE% values [62].

**Figure 2:**
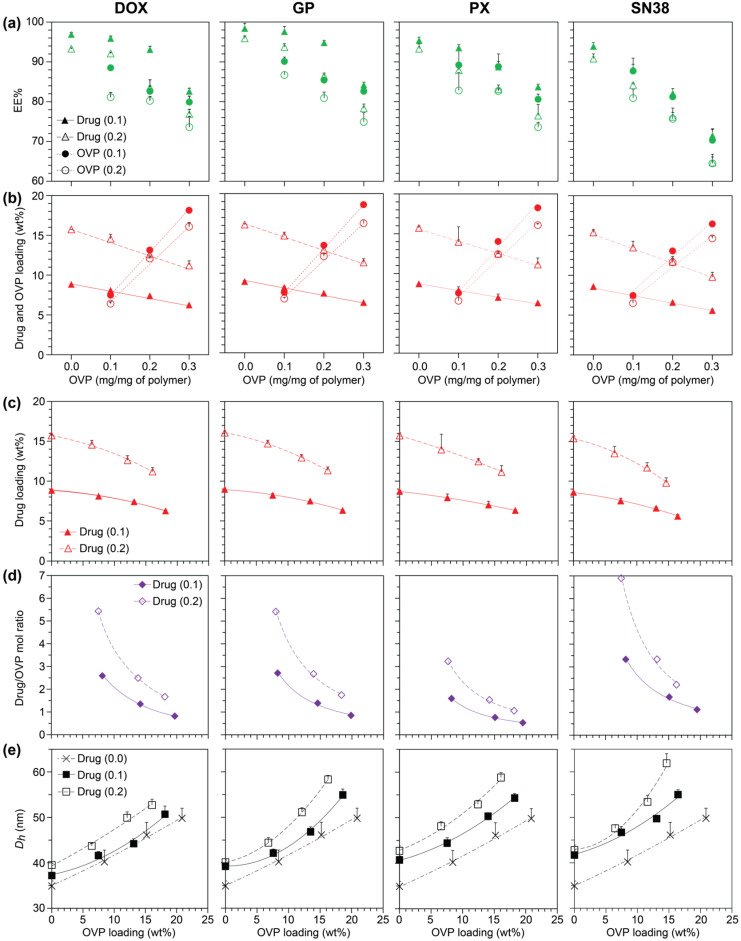
Characterisation of drug- and OVP-loaded PEG_10_PDL_10_ micelles. Columns correspond to the respective drugs indicated at the top. Drug and OVP (**a**) encapsulation efficiency percentage (EE%) and (**b**) loading (wt%) relative to the initial OVP loading (mg/mg of copolymer) used to prepare micelles at drug loadings of 0.1 and 0.2 mg/mg of copolymer (as indicated by numbers in brackets in legends). Symbols represent the experimental data; the (dashed/solid) lines are guides to the eyes. (**c**) Drug loading (wt%), (**d**) drug/OVP mole ratio, and (**e**) micelle *D_h_* (*D_h_* of blank micelles also shown for comparison) relative to the experimentally determined OVP loading (wt%) at drug loadings of 0.1 and 0.2 mg/mg of copolymer (as indicated by numbers in brackets in legends). All data are shown as the mean + SD (*n* = 3). Symbols represent the experimental data; dashed/dotted/solid lines represent power law fits to the data.

In the absence of OVP, drug EE% values were > 90% at both drug loadings (SM, Table S13), demonstrating good compatibility between the drugs and HBs. As exemplified with the PEG_10_PDL_10_ micelles (Figure 2a), in general, the EE% of both the drugs and OVP decreased with an increase in the drug or OVP loading (SM, Figures S24–S26). Nevertheless, even at the highest drug and OVP loadings, the drug and OVP EE% values were typically > 80 and 70%, respectively, for the PEG_14_PS_12_, PEG_10_PDL_10_ and PEG_10_PLA_10_ copolymeric micelles with DOX, GP and PX. These drug EE% values are comparable to other polymeric micelles reported in the literature [63,64,65,66] that were prepared in the absence of OVP. In contrast, lower drug and OVP EE% values (~55–75%) were obtained for the lower molecular weight PEG_5_PS_5_ micelles (SM, Figure S26), highlighting the limited encapsulation capacity of the smaller hydrophobic core. Similar trends in the drug EE% values with the HB length have also been reported for the encapsulation of etoposide and cyclosporine A in PEG-PCL micelles [67,68], and camptothecin in PEG-*b*-poly(valerolactone) (PEG-PVL) micelles [69]. For the PEG_14_PS_12_, PEG_10_PDL_10_ and PEG_10_PLA_10_ copolymeric micelles, SN38 proved to be the most challenging drug to encapsulate regardless of the copolymer composition, with drug EE% values of 70–76% at the highest OVP loading, which decreased further to 63–71% as the drug loading increased. Nevertheless, these EE% values for SN38 are generally an improvement over other micelle systems reported in the literature [62,70,71].

Considering the drug and OVP EE% values, their loading (wt%) were calculated relative to the initial OVP loading (Figure 2b and SM, Figures S24 and S25). Almost exclusively this revealed proportional relationships whereby an increase in the OVP wt% led to a concurrent decrease in the drug wt% regardless of the copolymer composition, drug identity or initial amount of drug encapsulated. This behaviour is unsurprising as both the OVP and drug contribute to the micelle mass, and therefore, an increase in the OVP loading at a constant drug loading would result in an overall lower weight fraction of drug.

**Figure 3:**
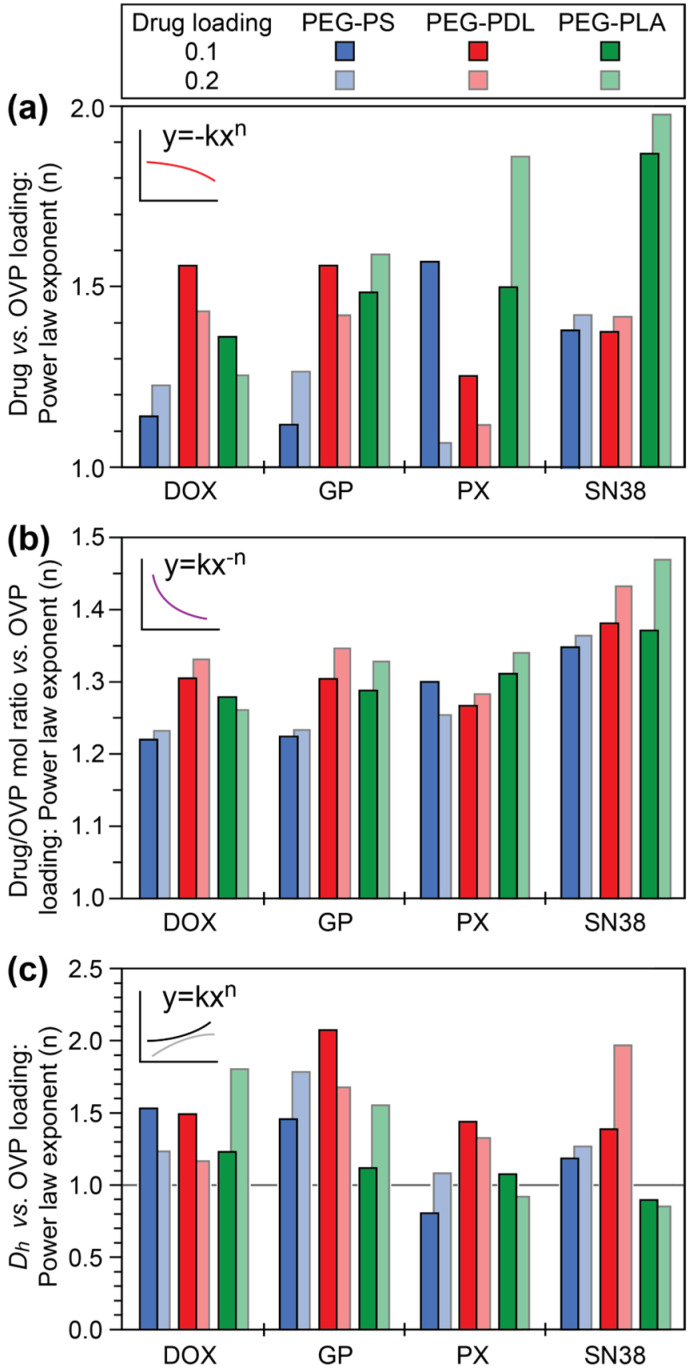
Comparison of power law exponents of fitted curves for trends in data correlating the (**a**) drug loading (wt%), (**b**) drug/OVP mole ratio and (**c**) *D_h_* to the OVP loading (wt%) for different drugs, drug loadings (0.1 and 0.2 mg/mg of copolymer) and copolymeric micelles.

To quantify the influence of OVP loading on the drug loading, the drug wt% was plotted against the OVP wt% (Figure 2c and SM, Figures S24 and S25), which were fitted with power laws of the format y = −kx^n^; coupled with a -k value, a ‘n’ exponent value of 1 indicated an inversely proportional relationship where drug encapsulation was not influenced by OVP encapsulation, whereas a ‘n’ value > 1 indicated a more drastic decrease in drug wt% with an increase in OVP wt% indicative of a greater influence of OVP on drug encapsulation. To assess trends between the different drugs, drug loadings and copolymeric micelles, the exponents were compared (Figure 3a). For the PEG_10_PDL_10_OVP_z_ micelles, OVP had a relatively strong influence on the co-encapsulation of DOX and GP as compared to PX and this trend persisted at higher drug loadings. This behaviour was reversed for the PEG_14_PS_12_OVP_z_ micelles, whereby OVP had little effect on the encapsulation of DOX and GP, but a more pronounced effect on PX encapsulation. The contrasting trends were attributed to drug and OVP interactions with the copolymeric micelle cores. For PEG_14_PS_12_OVP_z_ micelles, the polycyclic aromatic scaffolds of DOX and GP can form favourable π-π stacking interactions with the core allowing efficient encapsulation of both drug and OVP. The absence of these interactions in the PEG_10_PDL_10_OVP_z_ micelles results in OVP competing with DOX and GP and reducing their encapsulation potential. Given that PX is a significantly larger molecule compared to DOX and GP it is unsurprising that it is more efficiently incorporated in the amorphous and pliable core of the PEG_10_PDL_10_OVP_z_ micelles. However, at higher drug loading, PX encapsulation in the PEG_14_PS_12_OVP_z_ micelles was largely unaffected by OVP, possibly indicating changes in the micelle core structure, molecular dispersion of the drug or the mechanism of micellization. The encapsulation of SN38 was significantly influenced by OVP for all of the copolymers, especially PEG_10_PLA_10_OVP_z_, suggesting unfavourable interactions between the drug and OVP in the micelle core. In general, drug encapsulation in the PEG_10_PLA_10_OVP_z_ micelles was reduced by OVP regardless of the drug identity or loading, possibly resulting from the higher crystallinity of the micelle core. These results provide insights into the optimal drug–copolymer combinations for co-encapsulation with OVP, allowing the drug and OVP loading to be maximised.

Subsequently, the drug/OVP mole ratio as a function of OVP wt% was calculated (Figure 2d and SM, Figures S24 and S25), which is a function of the drug’s molar mass and would be expected to decrease as the OVP loading increased at a fixed drug loading. However, a reduction in the micelle’s ability to co-encapsulate the drugs due to the incorporation of OVP can lead to a more pronounced decrease in the drug/OVP mole ratio. To assess trends between the different drugs and micelles, the drug/OVP mole ratio as a function of OVP wt% was fitted with power laws of the format y = kx^−n^, whereby a ‘n’ exponent of 1 is indicative of the expected trend in the drug/OVP mole ratio and larger exponent values indicate a more drastic change leading to lower than expected ratios (Figure 3b). For DOX, GP and PX, the exponent values were similar (~1.2–1.3) regardless of the micelle composition, and these generally increased slightly as the drug loading was increased. In contrast, the exponent values were noticeably higher for SN38 at both drug loadings and with all micelles, consistent with a more drastic reduction in the drug/OVP mole ratio at higher OVP loadings.

DLS was employed to study the influence of drug and OVP co-encapsulation on the *D_h_* and PDI of the micelles (SM, Figures S27–S42 and Table S14). Initially, the data for the drug-loaded micelles in the absence of OVP was analysed. To assess the influence of drug loading alone and polymer composition on the micelle size, the *D_h,theo_* of the micelles was calculated at a drug EE% of 100% (SM, Figure S43 and Table S15). While the *D_h,theo_* increased linearly with drug loading for some copolymers, typically a smaller increase in size was noted as the drug loading was increased from 0.1 to 0.2 mg/mg of copolymer for most copolymer–drug combinations. For the PEG_14_PS_12_ micelles, the size linearly correlated with the drug loading regardless of the drug identity, although the magnitude of the increase was drug dependent. For the PEG_10_PDL_10_ and PEG_10_PLA_10_ micelles with GP, PX, and SN38, the addition of drugs at a loading of 0.1 mg/mg of copolymer resulted in a large increase in the size while a further increase in the drug loading led to relatively small increases in size. These results imply a change in the drug packing in the micelle core as the drug loading is increased or a change in the copolymer aggregation number (*N_agg_*). In the case of the former, this may involve a switch from a more homogeneous molecular dispersion of the drugs throughout the core to a heterogeneous distribution of drug aggregates/crystallites as the drug loading is increased. In comparison, the linear increase in size with drug loading observed for the PEG_14_PS_12_ micelles implies favourable interactions between the drugs and the hydrophobic block leading to homogeneous molecular dispersion of the drugs throughout the core regardless of the drug loading. These findings highlight the dynamic interplay between the drug and copolymer properties on the encapsulation of drugs and the micelle size. Unfortunately, a similar analysis could not be conducted in the presence of OVP as the combined influence of the OVP and drugs on changes in the micelle size could not be decoupled from one another.

The co-encapsulation of drugs and OVP typically resulted in an increase in the micelle *D_h_* as the OVP loading was increased, regardless of the copolymer composition or drug (Figure 2e and SM, Figures S24 and S25). Generally, the co-encapsulation of drugs resulted in an increase in the *D_h_* values of the micelles by ~5–10% as compared to their blank or OVP-loaded counterparts, although there were some distinct outliers that suggest specific drug–polymer interactions, disruption of core crystallinity, packing of the drugs in the core, or changes in the copolymer *N_agg_*. For example, the co-encapsulation of DOX and GP in the PEG_14_PS_12_OVP_z_ micelles led to relatively small increases in the *D_h_* (SM, Figure S24), while the addition of PX and SN38 to the PEG_10_PLA_10_OVP_z_ micelles led to large increases in the *D_h_* (SM, Figure S25). To compare the trends in *D_h_* as a function of OVP wt% for the different drugs and copolymeric micelles the data was fitted with power laws of the format y = kx^n^ (Figure 3c), whereby a ‘n’ exponent value of 1 indicated a proportional relationship, a ‘n’ value > 1 indicated a more drastic (more than proportional) increase in *D_h_* with an increase in OVP loading and a ‘n’ value < 1 indicated a less drastic increase in *D_h_* with an increase in OVP loading. For the PEG_10_PDL_10_OVP_z_ micelles, an increase in the OVP wt% resulted in exponent values greater than 1 regardless of the drug or the drug loading. In comparison, the increase in *D_h_* with OVP wt% was more proportional for the PEG_10_PLA_10_OVP_z_ micelles regardless of the drug at a lower loading. At a higher drug loading, the exponent was > 1 for DOX and GP and < 1 for PX and SN38, suggesting that the drug packing in the core or *N_agg_* is drug- and loading-dependent. Similarly, for the PEG_14_PS_12_OVP_z_ micelles, DOX and GP led to exponents > 1 while PX and SN38 led to proportional or < 1 relationships. These results provide quantitative relationships between *D_h_* and OVP loading for the different drugs, drug loadings, and drug–copolymer combinations, which can be used to predict and target the size of micelle formulations for specific applications.

### 3.3. Influence of Copolymer Composition and OVP Loading on pH-Dependent Drug Release

**Figure 4:**
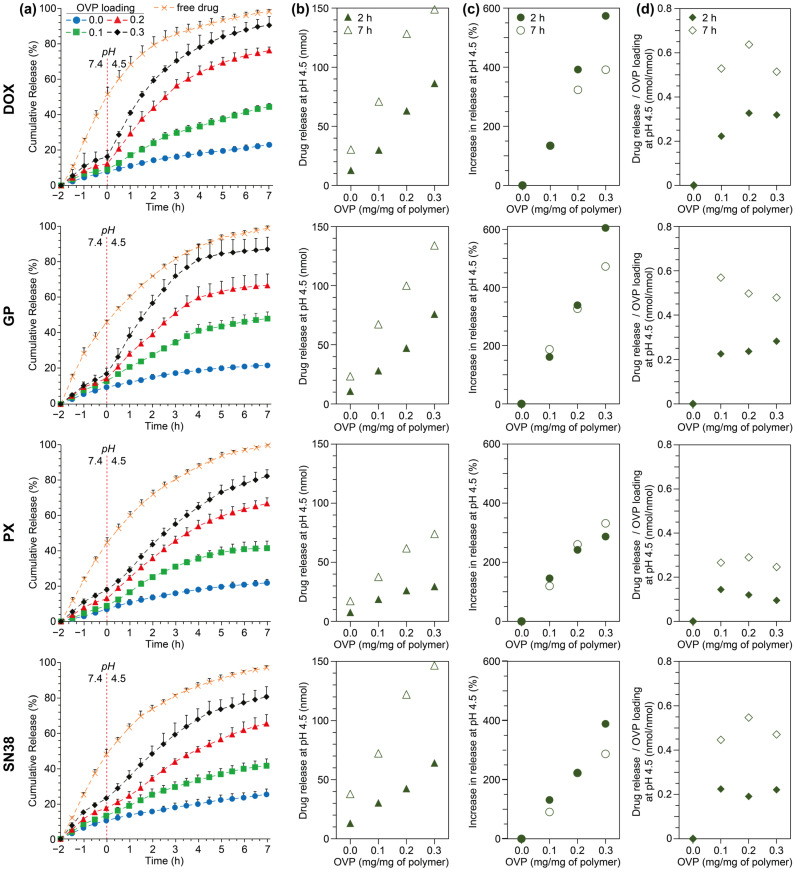
pH-dependent drug release from PEG_10_PDL_10_ micelles. Rows correspond to the respective drugs indicated on the left-hand side. (**a**) In vitro dialysis assay showing the cumulative release of drugs (all 0.1 mg/mg of copolymer) from free drug solutions and micelles with various OVP loadings (0, 0.1, 0.2, and 0.3 mg/mg of copolymer) against PBS (pH 7.4) for several hours (−2 to 0 h), followed by acetate buffer (pH 4.5) for 7 h (0 to 7 h); experiments were conducted at 22 ± 1 °C and the drug release was measured via UV-vis spectrophotometry (DOX, GP and SN38) or high-performance liquid chromatography (HPLC) (PX). Data are shown as the average cumulative release (%) + SD (*n* = 3). Only symbols represent the experimental data; the dashed lines are guides to the eyes. Release of the drugs at pH 4.5 after 2 and 7 h (excluding any release at pH 7) was analysed with respect to the OVP loading (0, 0.1, 0.2 and 0.3 mg/mg of copolymer) as (**b**) the mole amount of drug released, (**c**) the percentage increase in drug release relative to micelles without OVP, and (**d**) the amount of drug released relative to the amount of OVP loaded (nmol/nmol).

pH-dependent drug release from the OVP-loaded micelles was quantitatively assessed using an in vitro dialysis assay (SM, Table S16), whereby the micelles were initially exposed to simulated physiological conditions (PBS, 10 mM, pH 7.4) for 2 h before a switch to late endosomal conditions (acetate buffer, 10 mM, pH 4.5) (Figure 4 and SM, Figures S44–S46). For this study, the drug loading was kept constant at 0.1 mg/mg of copolymer and drug release from the micelles was compared to the diffusion of the free drug through the dialysis membrane, which is limiting. As expected, release of the free drug through the membrane follows first-order kinetics as it is only dependent on the concentration gradient and is not affected by the pH of the solution (SM, Table S17). As exemplified for the PEG_10_PDL_10_ micelles (Figure 4), there was a modest release of the drugs (typically between 5 and 20%) at pH 7.4, regardless of the drug identity, which increased as the OVP loading increased. This trend also extended to the PEG_14_PS_12_ and PEG_10_PLA_10_ micelles (SM, Figures S45 and S46). Drug release from the micelles at pH 7.4 was attributed to a combination of factors, including leakage from the micelles and the diffusion of unencapsulated drug, which was not separated from the micelles prior to the release study; however, considering the drug EE% values for the micelles (Figure 2) it is likely that most of the drug release at pH 7.4 results from the latter.

In the absence of OVP, a change in the pH to 4.5 had negligible influence on the release rate of the drugs from the micelles, with the release slowly continuing upwards or plateauing (reaching ~15–20% after 7 h), which was attributed to slow diffusion from the micelle core (Figure 4 and SM, Figures S45 and S46). Modelling of the release profiles provided good fits with Korsmeyer–Peppas and/or Higuchi models irrespective of the polymer composition, which is consistent with the slow diffusion of drugs from polymeric matrices (SM, Table S18). Similar release profiles and model fits have been commonly reported by others for the release of drugs from non-responsive copolymeric micelles. Eawsakul et al. reported that the release of a semi-synthetic andrographolide from PEG-PCL and PEG-PLA micelles fitted well with the Korsmeyer–Peppas model [72], while release of moxifloxacin and clarithromycin from PEG-PCL micelles was reported by Mohammadi et al. to fit the Higuchi model [73].

For the PEG_14_PS_12_OVP_z_, PEG_10_PDL_10_OVP_z_ and PEG_10_PLA_10_OVP_z_ micelles there was a significant increase in the drug release rate when the pH was changed to 4.5 as emphasised in first derivative release curves (SM, Figures S47–S49), which was more prominent at higher OVP loadings. Determination of the amount of drug released after 2 and 7 h at pH 4.5 revealed approximately linear correlations with the initial OVP loading (Figure 4b and SM, Figures S45b and S46b), which equated to 20–40, 35–60 and 45–85% drug release after 7 h as the initial OVP loading was increased from 0.1, 0.2 to 0.3 mg/mg of copolymer, respectively (SM, Table S19). This general behaviour was observed in all cases, largely independent of the copolymer composition, albeit with subtle differences in the release profile for different drugs. For instance, the pH switch caused an apparent burst release of DOX and GP from OVP-loaded micelles as accentuated in first derivative release curves, whereas as increase in the PX and SN38 release was slightly delayed and less pronounced (SM, Figures S47–S49). Given that similar trends are observed regardless of the copolymer composition, the differences in drug release profiles are believed to result from their physical parameters [36,37].

Generally, for the drug-loaded PEG_x_HB_y_OVP_0.2_ and PEG_x_HB_y_OVP_0.3_ micelles, the profile of the release curves following a switch to pH 4.5 mirrored the free drug diffusion profile at equivalent cumulative release, indicating that a significant fraction of the drugs are ejected from the core along with the OVP. These results are consistent with previous observations for PEG-PCL micelles [36], but further emphasise that oligoelectrolyte-mediated, pH-triggered release can be generally applied to non-responsive amphiphilic copolymeric micelles and different hydrophobic drugs. However, the copolymer molecular weight, particularly in the case of PEG-PS copolymers, appears to significantly influence the triggered release of OVP and drugs, with no acceleration in drug release being observed for the PEG_5_PS_5_ micelles in response to a change in the pH (SM, Figure S50).

To compare the effect of the initial OVP loading on the immediate (2 h) and sustained release (7 h) of drugs at pH 4.5, the percentage increase in drug release was calculated relative to the drug-loaded micelles (no OVP) at that time (Figure 4c and SM, Figures S45c and S46c). Consistent with the release profiles, an increase in OVP loading led to a drastic increase in drug release; however, the total increase at 2 and 7 h tended to be specific to drugs or drug–polymer combinations. Comparing the 2 h data, the increase in release of DOX and GP was greater than PX and SN38 for the PEG_10_PDL_10_OVP_z_ and PEG_10_PLA_10_OVP_z_ micelles. In comparison, the increase in the release of DOX from the PEG_14_PS_12_OVP_z_ micelles appeared retarded at higher OVP loadings while the increase in release of PX and SN38 was substantially greater. In general, the increase in release of all drugs at 2 h was similar or greater than at 7 h, indicating a substantial burst release of the drugs from the micelle core at pH 4.5. These results show that the co-encapsulation of OVP results in significant increases in drug release related to the OVP ejection for the micelle core, regardless of the polymer composition or drug identify, although some specific drug–polymer combinations may be optimal in maximising the release kinetics.

While an increase in the initial OVP loading led to an increase in the total amount of drug released and the rate of release, the data was further scrutinised to determine the amount of drug released relative to the OVP loaded (nmol/nmol ratio) at 2 and 7 h as a function of the initial OVP loading (Figure 4d and SM, Figures S45d and S46d). In most cases this revealed that the ratio relative to the initial OVP loading at 2 and 7 h was maximised when the initial OVP loading was 0.1 mg/mg of copolymer, and the ratio either remained consistent or decreased with further increases in the initial OVP loading. PEG_10_PDL_10_OVP_z_DOX_0.1_ and PEG_14_PS_12_OVP_z_SN38_0.1_ micelles were noticeable exceptions to this trend, with the ratio maximised at an initial OVP loading of 0.2 mg/mg of copolymer. While higher initial OVP loadings ultimately lead to greater drug release, these results indicate that there is a threshold amount of drug that is released per OVP and that this ratio varies depending on the drug–polymer combination, allowing the formulations to be optimised to maximise drug release and minimise the OVP concentration.

Modelling of the drug release profiles following a switch to pH 4.5 revealed several interesting differences in the release kinetics for certain copolymers or copolymer–drug combinations (SM, Table S18). In most cases, the release of drugs from the micelles was best fitted by the Korsmeyer–Peppas model, with exponents between 0.45 and 1 indicating anomalous, non-Fickian diffusion. However, drug release profiles from PEG_10_PDL_10_OVP_0.2_ and PEG_10_PDL_10_OVP_0.3_ micelles provided better fits to first-order kinetics regardless of the drug identity. Other notable exceptions that fitted first-order kinetics included PEG_10_PLA_10_OVP_0.2_ and PEG_10_PLA_10_OVP_0.3_ micelles with GP, and PEG_14_PS_12_OVP_0.2_ and PEG_14_PS_12_OVP_0.3_ micelles with PX. Nevertheless, for most of the OVP-loaded micelles, particularly at the higher OVP loadings, the MSC values for fitting to both the first-order and Korsmeyer–Peppas models were similar and >3, which is indicative of a good fit. Thus, the release profiles are likely reflective of a combination of pH-triggered burst release of the drugs caused by expulsion of OVP from the core, as indicated by the profile of the release curves and first derivative curves (Figure 4 and SM, Figures S45–S49), and a more gradual diffusion of any remaining drug fraction in the core. This behaviour is consistent with the OVP release profiles for the PEG_14_PS_12_OVP_z_ and PEG_10_PDL_10_OVP_z_ micelles (Figure 1), whereby OVP is instantaneously ejected from the micelle core when the pH is switched to 4.5. However, the observed drug release profiles for the PEG_10_PLA_10_OVP_z_ micelles, which showed a burst or accelerated release at pH 4.5 (SM, Figure S46), are inconsistent with the sustained OVP release profiles (Figure 1). A possible explanation for this behaviour is that as the OVP becomes ionised, it causes hydration and swelling of the core, which leads to a rapid release of drugs followed by a more sustained release of the OVP. Overall, the release results demonstrated that the OVP-mediated, pH-triggered release of drugs is broadly compatible with compositionally diverse micelles and different drug classes.

### 3.4. Effect of Polymer Composition and OVP Loading on the Cytotoxicity of Drug-Loaded Micelles

**Figure 5:**
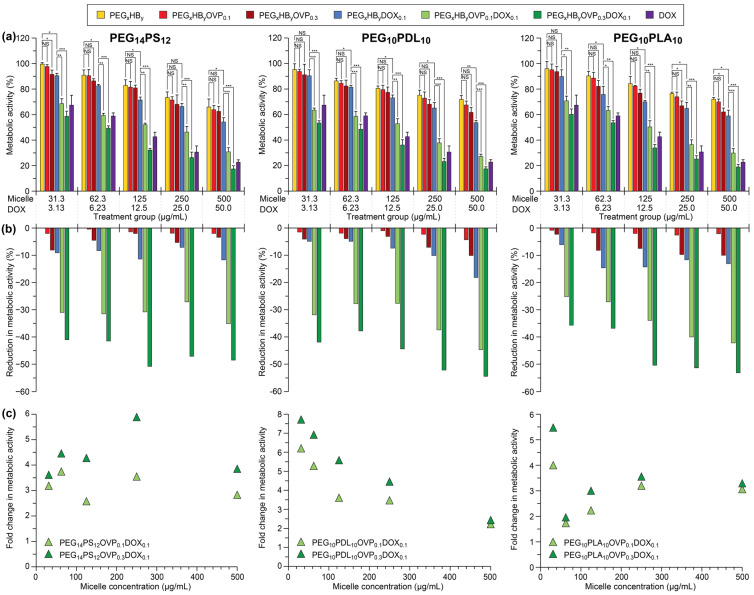
(**a**) In vitro percentage metabolic activity of SKOV-3 cells relative to negative controls (untreated cells) when treated with micelles (PEG_14_PS_12_ left, PEG_10_PDL_10_ middle, PEG_10_PLA_10_ right) and free DOX at various doses, following incubation for 48 h (a subset of this data has previously been published in a different format [36]); treatment group concentrations refer to the micelle (31.3–500 µg/mL) and DOX concentrations (3.13–50.0 µg/mL) if present. Data are shown as the mean metabolic activity (%) ± SD (*n* = 3); NS, *, ** and *** represent not significant (*p* ≥ 0.05), *p* < 0.05, *p* < 0.01 and *p* < 0.001, respectively. (**b**) Percentage reduction in metabolic activity of OVP and DOX-loaded micelles relative to blank (PEG_x_HB_y_) micelles. (**c**) Fold change in reduction in metabolic activity of PEG_x_HB_y_OVP_0.1_DOX_0.1_ and PEG_x_HB_y_ OVP_0.3_DOX_0.1_ micelles relative to PEG_x_HB_y_DOX_0.1_ micelles following subtraction of any reduction associated with OVP.

Previously, we reported that the co-encapsulation of OVP (0.3 mg/mg of copolymer) in DOX-loaded PEG_14_PS_12_, PEG_10_PDL_10_ and PEG_10_PLA_10_ micelles led to a significant increase in their cytotoxicity in human ovarian adenocarcinoma (SKOV-3) cells, regardless of the copolymer composition [36]. However, the influence of OVP loading on cytotoxicity remained unknown. Furthermore, the broad applicability of the oligoelectrolyte-mediated, pH-triggered release of drugs from compositionally diverse micelles had yet to be proven with different drugs and cell lines. Therefore, DOX- and OVP-loaded (0.1 or 0.3 mg/mg of copolymer) PEG_14_PS_12_, PEG_10_PDL_10,_ and PEG_10_PLA_10_ micelles were initially tested using a 3-(4,5-dimethylthiazol-2-yl)-2,5-diphenyltetrazolium bromide (MTT) metabolic activity assay with SKOV-3 cells, and compared to the blank PEG_x_HB_y_ micelles, DOX-loaded PEG_x_HB_y_DOX_0.1_ micelles, OVP-loaded PEG_x_HB_y_OVP_Z_ micelles and the free drug (Figure 5).

All micelle formulations and the free drug displayed a concentration dependent change in metabolic activity, with the DOX- and OVP-loaded micelles and free drug displaying significantly greater reductions in metabolic activity as compared to the other micelle formulations (Figure 5a). In general, the change in metabolic activities were consistent across the different formulations regardless of the copolymer used. To better compare the impact of the different micelle formulations, the percentage reduction in metabolic activity was calculated relative to the blank micelles (Figure 5b). For the OVP-loaded micelles (no DOX), the reduction in metabolic activity was typically <10% regardless of the micelle composition or concentration, and was significantly lower at an OVP loading of 0.1 mg/mg of copolymer, implying that OVP is generally well tolerated. The PEG_x_HB_y_DOX_0.1_ micelles (no OVP) displayed reductions in metabolic activity between 5 and 15% and were generally higher for the PEG_x_PLA_y_DOX_0.1_ micelles, possibly indicating greater release of DOX through either swelling or degradation of the PLA core. In comparison, the DOX- and OVP-loaded micelles resulted in significantly larger reductions in the metabolic activity ranging from 25–45 and 35–55% for OVP loadings of 0.1 and 0.3 mg/mg of copolymer, respectively. Considering the relatively minor reduction in metabolic activity resulting from the OVP-loaded micelles (no DOX) and DOX-loaded micelles (no OVP), it is evident that the large reductions in metabolic activity for the PEG_x_HB_y_OVP_z_DOX_0.1_micelles results from the OVP-mediated release of DOX from the micelles, although possible synergic effects between the DOX and OVP may also play a role.

To emphasise the significant cytotoxicity enhancement of the PEG_x_HB_y_OVP_z_DOX_0.1_micelles over their non-responsive PEG_x_HB_y_DOX_0.1_ counterparts (no OVP), the fold change in reduction in metabolic activity was calculated for the former relative to the latter, as a function of concentration (Figure 5c). Surprisingly, this revealed some interesting trends that were specific to the different copolymers. For the PEG_14_PS_12_OVP_Z_DOX_0.1_ micelles, the fold change was relatively consistent at ~3 and 4 for initial OVP loadings of 0.1 and 0.3 mg/mg of copolymer, regardless of the concentration. In contrast, there was a clear downwards trend in the fold change with concentration for the PEG_10_PDL_10_OVP_Z_DOX_0.1_ micelles, with exceptionally high values (~6–8-fold change) at the lowest concentration tested. Similarly, the largest fold change (~4–5.5) was noted at the lowest concentration for the PEG_10_PLA_10_OVP_Z_DOX_0.1_ micelles; however, as the concentration increased the fold change drastically decreased to ~2 before increasing again to ~3. While further experiments are required to establish the factors responsible for driving these trends, it is evident that the choice of copolymer and the micelle/drug concentration has a large influence on the effectiveness of drug delivery to the intracellular targets resulting in different reduction in the metabolic activities observed with the DOX- and OVP-loaded micelles. Nevertheless, in all cases a significant fold change is observed relative to the non-responsive micelles, demonstrating the broad applicability of oligoelectrolyte-mediated, pH-triggered release of drugs to compositionally diverse micelles.

**Figure 6:**
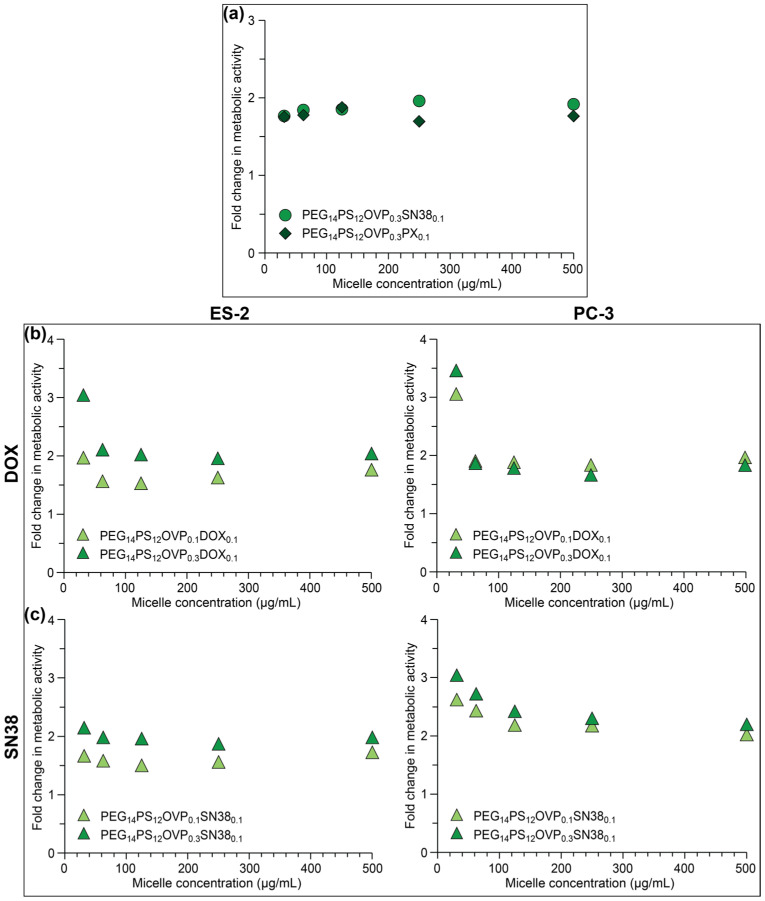
Fold change in reduction in metabolic activity as a function of micelle concentration for drug-loaded PEG_14_PS_12_OVP_z_ micelles relative to drug-loaded PEG_14_PS_12_ micelles (no OVP), following incubation for 48 h; drug concentrations (3.13-50.0 µg/mL) are 10 times less than micelle concentrations. Fold change for (**a**) PEG_14_PS_12_OVP_0.3_SN38_0.1_ and PEG_14_PS_12_OVP_0.3_PX_0.1_ micelles with SKOV-3 cells, (**b**) PEG_14_PS_12_OVP_0.1_DOX_0.1_ and PEG_14_PS_12_OVP_0.3_DOX_0.1_ micelles with ES-2 (**left**) and PC-3 (**right**) cells and (**c**) PEG_14_PS_12_OVP_0.1_SN38_0.1_ and PEG_14_PS_12_OVP_0.3_SN38_0.1_ micelles with ES-2 (**left**) and PC-3 (**right**) cells.

To assess the effectiveness of different drugs in combination with the OVP-loaded micelles, cytotoxicity assays were conducted on SKOV-3 cells using SN38- and PX-loaded PEG_14_PS_12_OVP_0.3_ micelles and compared to micelles without OVP (SM, Figure S51). All treatment groups displayed a concentration dependent change in metabolic activity. Typically, the PEG_14_PS_12_OVP_0.3_SN38_0.1,_ and PEG_14_PS_12_OVP_0.3_PX_0.1_ micelles resulted in similar reductions in the metabolic activities as the free drugs. In the absence of OVP, the PEG_14_PS_12_SN38_0.1_ and PEG_14_PS_12_PX_0.1_ micelles also displayed greater reductions in the metabolic activities as compared to blank and DOX-loaded micelles (Figure 5), implying that SN38 and PX were more cytotoxic to the SKOV-3 cells (*cf.* DOX). This was further confirmed through determination of the percentage reduction in metabolic activity relative to the blank micelles, which revealed reductions of ~25 and 30% for the PEG_14_PS_12_SN38_0.1_ and PEG_14_PS_12_PX_0.1_ micelles, respectively. For the PEG_14_PS_12_OVP_0.3_SN38_0.1_ and PEG_14_PS_12_OVP_0.3_PX_0.1_ micelles, these reductions were approximately doubled, as emphasised in the fold change in reduction in metabolic activity, which was ~1.8–2 regardless of the concentration or drug (Figure 6a). These results demonstrate that oligoelectrolyte-mediated drug release and enhanced cytotoxicity appear to be broadly applicable to other drugs, although the cytotoxicity of the drug appears to play a prominent role in the extent of the fold change observed.

Subsequently, we extended investigations to other cell lines, including the ovarian cancer ES-2 and prostate cancer PC-3 cell lines (SM, Figures S52 and S53). Assays were conducted using the PEG_14_PS_12_ micelles, two different OVP loadings (0.1 and 0.3 mg/mg of copolymer), and two different drugs (DOX and SN38). Interestingly, there were differences in how the two cell lines responded, particularly with respect to the OVP loading (vide infra). For the ES-2 and PC-3 cell lines, the blank PEG_14_PS_12_ micelles displayed a comparable reduction in metabolic activity at equivalent concentrations, which was also consistent with the values obtained for the SKOV-3 cell line (Figure 5a). The ES-2 and PC-3 cell lines also responded similarly to the inclusion of OVP in the micelles, with a small reduction in the metabolic activity of ~5–10% for the PEG_14_PS_12_OVP_0.1_ micelles (*cf.* blank micelles), increasing to ~10–15% for the PEG_14_PS_12_OVP_0.3_ micelles. As previously reported for PEG-PCL micelles, the small reduction in metabolic activity suggests that OVP has some inherent cytotoxicity that may result from disruption of the endosomal/lysosomal vesicles [36].

In the absence of OVP, the PEG_14_PS_12_DOX_0.1_ micelles resulted in similar reductions in metabolic activity in both the ES-2 and PC-3 cell lines, with values ranging from ~10–20% (*cf.* blank micelles) (SM, Figure S52). In general, co-encapsulation of OVP in the PEG_14_PS_12_OVP_Z_DOX_0.1_ micelles led to a further reduction in metabolic activity. In ES-2 cells, the OVP loading led to larger differences in metabolic activity that alone could not be accounted for by the inherent reduction in metabolic activity due to OVP. For instance, the PEG_14_PS_12_OVP_0.1_DOX_0.1_ and PEG_14_PS_12_OVP_0.3_DOX_0.1_ micelles displayed reductions in the metabolic activities ranging from ~30–45 and 50–60%, respectively. In comparison, the response of the PC-3 cells to the PEG_14_PS_12_OVP_0.1_DOX_0.1_ and PEG_14_PS_12_OVP_0.3_DOX_0.1_ micelles were similar, with reductions in the metabolic activities ranging from ~35–50%. The disparate behaviour observed across the two cell lines with OVP loading is believed to result from differences in the endolysosomal systems. Importantly, for both the ES-2 and PC-3 cell lines, the PEG_14_PS_12_OVP_z_DOX_0.1_ micelles resulted in a large reduction in metabolic activity relative to PEG_14_PS_12_DOX_0.1_ micelles as evident in the ~2-fold change in metabolic activity between the micelles (Figure 6b).

Experiments conducted on the ES-2 and PC-3 cell lines using SN38-loaded micelles (SM, Figure S53) revealed very similar trends to those observed for the DOX-loaded micelles, albeit with slightly lower metabolic activity values due to the higher cytotoxicity of SN38. The average fold change in metabolic activity for the PEG_14_PS_12_OVP_z_SN38_0.1_ micelles relative to PEG_14_PS_12_SN38_0.1_ micelles revealed that the OVP-loaded micelles displayed a greater enhancement in cytotoxicity for the PC-3 cells (*cf.* ES-2 cells). Overall, the in vitro experiments demonstrate that oligoelectrolyte augmented micelles have significantly higher cytotoxicities than their traditional counterparts, and that this strategy is broadly applicable to compositionally diverse non-responsive micelles and different hydrophobic drug classes.

## 4. Conclusions

The influence of copolymer composition on the OVP-mediated release of drugs from micelles and their cytotoxicity was studied using PEG-PS, PEG-PLA and PEG-PLA copolymers. Despite the significantly different chemical composition and *T_g_* of the core forming block, OVP encapsulation in the copolymer micelles proceeded efficiently with an inverse relationship between the OVP loading and the EE%. Encapsulation of OVP at pH 7.3 resulted in a loading-dependent increase in the micelle *D_h_*, the extent of which correlated with the copolymer molecular weight and *T_g_* of the hydrophobic block. For all micelles, a decrease in the pH below 6.5 led to a decrease in the *D_h_*, which was consistent with OVP ionisation and release, and was further supported by NMR spectroscopy and UV-vis spectrophotometry. The release of OVP from PEG_14_PS_12_ and PEG_10_PDL_10_ micelles at pH 4.5 was found to follow first-order kinetics, which is indicative of the abrupt expulsion of OVP from the micelles, whereas the release was incomplete from PEG_5_PS_5_ micelles and revealed fits with Higuchi and Korsmeyer–Peppas models. These results indicated that the molecular weight of the HB is responsible for differences in the distribution and packing of OVP in the micelle core, and future studies should focus on investigating the influence of copolymer molecular weight and HLB. In contrast, OVP release from PEG_10_PLA_10_ micelles followed a near linear release profile, which may result from stronger interactions between the OVP and PLA core coupled with polymer relaxation. From this perspective, it would be interesting to study the effect of glycolide inclusion and specifically the lactide-to-glycolide ratio.

Drug and OVP co-encapsulation in the PEG_14_PS_12_, PEG_10_PDL_10,_ and PEG_10_PLA_10_ micelles provided moderate to high EE% values, with an increase in the OVP or drug loading correlating with lower EE% values. Generally, the drug EE% values (at a fixed drug loading) were close regardless of the micelle composition, while the drug identity had a larger influence on the EE%. Except for PEG_5_PS_5_ micelles, all micelles, regardless of composition, OVP/drug loading, or drug identity, displayed a triggered release of drugs when the pH was switched from 7.4 to 4.5. Importantly, the drug release profiles for the PEG_10_PLA_10_ micelles were similar to the other micelles despite the gradual release of OVP, indicating that OVP does not necessarily need to be ejected from the micelle core to allow rapid drug release, and swelling of the core appears to be sufficient. In vitro cell studies revealed that the OVP- and drug-loaded micelles displayed significantly higher cytotoxicities than drug-loaded micelles in the absence of OVP, regardless of the copolymer composition, specific drug, or cell line. However, the extent of the increases in the cytotoxicity as measured by the fold change in metabolic activity was highly dependent on these variables. An increase in the OVP loading resulted in an increase in the cytotoxicity; however, the extent of which was dependent on the cell line. This fundamental study highlights the broad scope and versatility of the OVP-mediated, pH-triggered strategy for enhanced drug release and cytotoxicity of compositionally diverse micelles. In the future, studies are required to validate whether this enhanced drug release observed in vitro translates to an in vivo setting.

## Figures and Tables

**Table 1:** Composition and molecular weight characteristics of the polymers used in this study.

Polymer	*f_HB_* (kDa) ^a^	*M_n_*_,NMR_ (kDa) ^a^	*M_n_*_,GPC_ (kDa) ^b^	*Ð* ^b^
PEG_5_PS_5_	0.48	10.7	8.9	1.12
PEG_14_PS_12_	0.45	26.5	17.9	1.09
PEG_10_PDL_10_	0.49	19.8	15.4	1.11
PEG_10_PLA_10_	0.49	19.5	14.3	1.22
OVP	-	1.3	1.3	1.22

^a^ *f_HB_* and *M_n_*_,NMR_ were determined from ^1^H NMR spectroscopy. ^b^ *M_n_*_,GPC_ and dispersity (*Đ*) were determined from gel permeation chromatography (GPC) using a conventional column calibration with PEG standards.

**Table 2:** Characterisation of blank and OVP-loaded micelles prepared in PBS.

Copolymer	OVP Loading (mg/mg)	CMC (mg/L) ^a^	OVP EE% ^b^	OVP Loading (wt%) ^c^	*D_h_* (nm) at pH 7.3 ^d^	PDI at pH 7.3 ^d^	*D_h,theo_* (nm) ^e^	*ζ* (mV) ^f^
PEG_5_PS_5_	0	0.60	---	---	25.1 ± 1.0	0.13 ± 0.02	25	−1.1 ± 0.2
	0.1	0.70	92.9 ± 1.6	8.5 ± 0.2	28.6 ± 0.3	0.15 ± 0.01	29	3.6 ± 0.4
	0.2	0.70	86.4 ± 1.7	14.7 ± 0.3	31.9 ± 0.7	0.17 ± 0.01	33	4.8 ± 0.6
	0.3	0.65	79.5 ± 1.8	19.3 ± 0.5	34.2 ± 0.4	0.20 ± 0.01	37	6.5 ± 0.3
PEG_14_PS_12_	0	0.75	---	---	61.5 ± 1.9	0.16 ± 0.02	62	−0.9 ± 0.3
	0.1	0.70	96.2 ± 1.1	8.8 ± 0.1	74.1 ± 2.7	0.18 ± 0.02	75	−0.2 ± 0.2
	0.2	0.75	94.2 ± 0.6	15.9 ± 0.1	80.5 ± 2.1	0.20 ± 0.01	82	0.7 ± 0.2
	0.3	0.80	92.9 ± 1.9	21.8 ± 0.6	85.9 ± 4.4	0.24 ± 0.01	88	1.5 ± 0.6
PEG_10_PDL_10_	0	0.50	---	---	34.9 ± 1.1	0.14 ± 0.01	35	−2.4 ± 0.4
	0.1	0.50	92.1 ± 0.8	8.4 ± 0.1	40.2 ± 2.6	0.16 ± 0.01	41	−0.7 ± 0.2
	0.2	0.65	89.6 ± 1.3	15.2 ± 0.3	46.1 ± 2.8	0.17 ± 0.01	47	−0.2 ± 0.2
	0.3	0.60	87.9 ± 1.8	20.9 ± 0.5	49.8 ± 2.2	0.19 ± 0.01	52	0.4 ± 0.3
PEG_10_PLA_10_	0	0.60	---	---	50.1 ± 1.2	0.17 ± 0.02	50	−2.4 ± 0.6
	0.1	0.60	95.5 ± 1.1	8.7 ± 0.1	62.4 ± 5.1	0.18 ± 0.03	63	0.8 ± 0.2
	0.2	0.70	89.9 ± 2.3	15.2 ± 0.5	67.8 ± 3.5	0.21 ± 0.02	70	1.7 ± 0.1
	0.3	0.70	82.9 ± 1.6	19.9 ± 0.5	72.1 ± 4.4	0.22 ± 0.02	77	3.0 ± 0.2

^a^ Critical micelle concentration (CMC) estimated at pH 7.4 from ratiometric fluorescent emission of a pyrene probe. ^b^ Oligo(vinyl pyridine) (OVP) encapsulation efficiency percentage (EE%) determined from UV-vis spectrophotometry quantification of unencapsulated OVP isolated through centrifugation of micelle formulations. ^c^ OVP loading calculated from the initial loading and the EE%. ^d^ Hydrodynamic diameter (*D_h_*) and polydispersity index (PDI) of the micelles in PBS-d at pH 7.3, as determined by dynamic light scattering (DLS). ^e^ Theoretical hydrodynamic diameter (*D_h,theo_*) at 100% OVP EE% was calculated from the experimentally determined EE% and *D_h_*. ^f^ Zeta potential (*ζ*) was determined from measurement of the electrophoretic mobility of micelles in 1 mM NaCl at pH 7.4.

## Data Availability

Data will be made available upon request.

## Supplementary Materials

The following supporting information can be downloaded at: https://www.mdpi.com/article/10.3390/polym18020247/s1, Figures S1–S4: Structural and thermal characterization of the copolymers; Figure S5: Visual appearance of blank and OVP-loaded micelles; Figures S6–S9: CMC determination of the copolymers; Figures S10–S13: DLS PSDs of blank and OVP-loaded micelles as a function of pH; Figure S14: *D_h,theo_* at 100% OVP EE%; Figures S15–S18: ^1^H-NMR spectroscopy of blank and OVP-loaded micelles as a function of pH; Figures S19–S22: UV-vis spectrophotometry of blank and OVP-loaded micelles as a function of pH; Figure S23: In vitro pH-triggered release of OVP from micelles; Figures S24–S26: EE%, drug and OVP loading, D*_h_* as a function of OVP loading for the drug- and OVP-loaded micelles; Figures S27–S42: DLS PSDs for drug- and OVP-loaded micelles; Figure S43: *D_h,theo_* at 100% drug EE%; Figure S44: Summary of drug release profiles for blank and OVP-loaded micelles; Figures S45 and S46: pH-dependent drug release profiles and accompanying analysis from blank and OVP-loaded micelles; Figures S47–S49: First derivative drug release curves for micelles with and without OVP; Figure S50: Drug release profiles and first derivatives curves for blank and OVP-loaded PEG_5_PS_5_ micelles; Figures S51–S53: Comparative cytotoxicity of different drug- and OVP-loaded micelles; Table S1: Summary of thermal properties of block copolymers; Table S2: Formulation details for preparation of OVP-loaded micelles; Table S3: Formulation details for CMC determination; Tables S4–S7: Formulation details for pH adjustment of micelle solutions; Table S8: PDI values of micelles as a function of pH; Table S9: Summary of *D_h,theo_* values for OVP-loaded micelles; Table S10: Formulation details for preparation of micelles used in OVP release studies; Table S11: Kinetic modelling parameters for in vitro OVP release studies from micelles; Table S12: Formulation details for preparation of drug- and OVP-loaded micelles; Table S13: EE% of drugs and OVP for drug- and OVP-loaded micelles; Table S14: DLS PDI values for drug- and OVP-loaded micelles; Table S15: Summary of *D_h,theo_* values for drug-loaded micelles; Table S16: Formulation details for preparation of drug and OVP-loaded micelles for in vitro release studies; Table S17: Kinetic modelling parameters for drug diffusion; Table S18: Kinetic modelling parameters for in vitro drug release studies from blank and OVP-loaded micelles; Table S19: Summary of drug loading and release from drug- and OVP-loaded micelles. References [36,37,74,75,76,77,78] are cited in Supplementary Materials.
