# Supplementary material for: Correlation of Polymer–drug Composition with Micelle Properties, Performance, and Cytotoxicity for the Oligoelectrolyte-mediated pH-triggered Release of Hydrophobic Drugs
Source: Polymers (Basel). 2026 Jan 16;18(2):247. doi: 10.3390/polym18020247 (PMC12846188; doi:10.3390/polym18020247)
Supplement: Supplementary file 1 [file polymers-18-00247-s001.zip › polymers-4102449-supplementary.pdf]

Supplementary Materials

Correlation of Polymer–drug Composition with Micelle Properties Performance, and Cytotoxicity for the Oligoelectrolyte-mediated pH-triggered Release of Hydrophobic Drugs

*Md. Saddam Hussain[1,2], Riya Khetan[3], Hugo Albrecht[3], Marta Krasowska[4], Anton Blencowe[1]**

[1] Applied Chemistry and Translational Biomaterials (ACTB) Group, Centre for Pharmaceutical Innovation (CPI), School of Pharmacy and Biomedical Sciences, College of Health, Adelaide University, Adelaide, SA 5000, Australia; mdsaddam.hussain@adelaide.edu.au

[2] Department of Pharmacy, Faculty of Biological Sciences, Noakhali Science and Technology University, Noakhali 3814, Bangladesh

[3] Centre for Pharmaceutical Innovation (CPI), School of Pharmacy and Biomedical Sciences, College of Health, Adelaide University, Adelaide, SA 5000, Australia; riya.khetan@adelaide.edu.au; hugo.albrecht@adelaide.edu.au

[4] Surface Interactions and Soft Matter (SISM) Group, Future Industries Institute, Adelaide University, Mawson Lakes, SA 5095, Australia; marta.krasowska@adelaide.edu.au

* Correspondence: anton.blencowe@adelaide.edu.au

Detailed experimental

Critical micelle concentration (CMC) of the copolymers

The CMCs of the diblock copolymers in the absence and presence of oligo(2-vinyl pyridine) (OVP) were determined using fluorescence spectroscopy with pyrene as a probe [1]. Stock solutions of pyrene, copolymers, and OVP were individually prepared in acetone and specific volumes were combined in vials (SI, **Table S3**). Phosphate buffered saline (PBS; 10 mM, pH 7.4) was added dropwise with gentle agitation and the vials were heated at 60 °C on a heating block (Ratek Instruments, Australia) for 3 h and then placed under vacuum (0.1 mbar) at 21 ± °C for 1 h to remove any residual acetone. The volume of the solutions was readjusted to 2 mL with water to compensate for any water evaporation during the removal of acetone. This process resulted in a series of solutions with copolymer concentrations ranging from 0.0001 to 0.5 mg/mL, with or without OVP (0.1, 0.2 or 0.3 mg/mg of copolymer) and a pyrene concentration of 6×10^{-7} M. The pyrene fluorescence emission spectra were recorded between 350 and 470 nm at 2000 nm/s and 3 nm bandwidth following excitation at λ_{ex} = 334 nm. The intensity ratio of emission peaks at λ_{em} = 372 and 384 nm (I_{384}/I_{372}) was plotted against the copolymer concentration and the CMC was determined from the low concentration plateau (SI, **Figures S6–9**).

Preparation of micelle solutions for dynamic light scattering (DLS), proton nuclear magnetic resonance (^{1}H NMR spectroscopy) and ultraviolet-visible (UV-vis) spectrophotometry

Blank and OVP-loaded (0.1, 0.2 and 0.3 mg/mg of copolymer) copolymer micelles were prepared *via* the solvent evaporation technique. The copolymers and OVP were separately dissolved in acetone at a concentration of 1 mg/mL and then specific volumes of these solutions were combined in vials (SI, **Tables S4–7**). PBS-d (10 mM, pH 7.3) was added dropwise under gentle agitation and the solutions were heated at 60 °C on a heating block for 3 h to remove acetone. Subsequently, the solutions were placed under vacuum (0.1 mbar) at 21 ± °C for 1 h to ensure the removal of any residual acetone. The pH of the solutions was then adjusted to 6.4, 5.5, 4.6, and 3.7 using 1 M DCl and the total volume was adjusted to 2 mL with D_2O. Prior to analysis, the micelle solutions were filtered through 0.22 µm syringe filters (Millipore).

Preparation of micelle solutions for determination of OVP and drug encapsulation efficiency

The solvent evaporation approach was used for the co-encapsulation of drugs and/or OVP. Initially, stock solutions of the drugs (1 mg/mL), OVP (1 mg/mL) and the copolymers (1 mg/mL) were prepared in acetone or tetrahydrofuran (THF) and specific volumes were combined in vials, followed by the dropwise addition of an equal volume of PBS (10 mM, pH 7.4) under gentle agitation (SI, **Table S12**). The solutions were heated at 60–65 °C for 3–4 h to evaporate the organic solvent. To remove traces of the solvents, the solutions were placed under vacuum (0.01 mbar, 40 min), and then additional water was added on a mass

basis as required to correct to the desired micelle concentration (1 mg/mL). The micelle solutions were then centrifuged (15 krpm, 40 min) to separate any free drug and/or OVP in the solution. The supernatants were carefully recovered and analyzed *via* DLS to determine the particle size distributions (PSDs) and polydispersity index (PDI) values of the micelles (SI, **Figure S13**), and any precipitates were redissolved in either methanol (doxorubicin (DOX) samples) or ethanol (gossypol (GP), paclitaxel (PX) and 7-ethyl-10-hydroxycamptothecin (SN38) samples). The solutions were analyzed *via* UV-vis spectrophotometry and/or high-performance liquid chromatography (HPLC) against standard curves to determine the encapsulation efficiency percentage (EE%) of OVP and drugs [2]. All experiments were conducted in triplicate. The EE% values were calculated according to the following equation:

$$\mathrm{EE\%} = \frac{\text{Mass of drug or OVP in the micelles}}{\text{Mass of drug or OVP used in the formulation}}\ x\ 100\%$$

In vitro OVP release studies

The *in vitro* release of OVP from the copolymer micelles was investigated as function of pH using a dialysis assay. Micelle solutions were prepared in PBS (10 mM, pH 7.4) as previously described (SI, **Table S10**) and 2 mL aliquots were transferred to Float-a-Lyzers (Spectra-Por, USA; molecular weight cut-off (MWCO) 100 kDa) containing a stirrer bar and placed in vials containing PBS (10 mM, pH 7.4, 10 mL) as the receiving solution complete with stirrer bars. Experiments were conducted under continuous stirring (130 rpm) at 22 ± 1 °C. After monitoring the OVP release at pH 7.4 for 2 h at 30 min intervals, the receiving solution was replaced with NaOAc buffer (10 mM, pH 4.5, 10 mL) and the entire receiving solution (10 mL) was collected at 30 min intervals for 7 h and replaced with fresh NaOAc buffer (10 mL) each time. The concentration of OVP in the release media at each time point was determined *via* UV-vis spectrophotometry at λ = 264 nm against a calibration curve recorded in NaOAc buffer (pH 4.5). All experiments were repeated in triplicate. Control experiments (free OVP) were conducted in a similar manner by measuring the release of OVP (0.1, 0.2 and 0.3 mg/mL) dissolved in NaOAc buffer (10 mM, pH 4.5) against NaOAc buffer (10 mM, pH 4.5) as the receiving solution.

In vitro drug release studies

The *in vitro* release of drugs (DOX, GP, PX and SN38) from OVP-loaded (0.1, 0.2 and 0.3 mg/mg of copolymer) micelles was investigated as function of pH using a dialysis assay. Micelle solutions were prepared in PBS (10 mM, pH 7.4) as previously described (SI, **Table S16**) and 2 mL aliquots were transferred to Float-a-Lyzers (MWCO 100 kDa) containing a stirrer bar and placed in plastic vials containing PBS (10 mM, pH 7.4, 10 mL) as the receiving solution complete with stirrer bars. The plastic vials were wrapped with aluminium foil to prevent photolytic degradation of the drugs. Experiments were conducted under continuous stirring (150 rpm) at 22 ± 1 °C. The entire receiving solution (10 mL) was collected at 30 min intervals (30, 60 and 90 min) and replaced with fresh PBS (10 mL). For DOX-loaded micelles,

the receiving solution was replaced with acetate buffer (10 mM, pH 4.5, 10 mL) at 120 min and the receiving solution (10 mL) was collected at regular intervals (150, 180, 210, 240, 270, 300, 330, 360, 390, 420, 450, 480, 510 and 540 min) and replaced with fresh buffer (10 mL). For GP-loaded micelles, the same sampling procedure was followed using 0.5% v/v Tween 20 in PBS (10 mM, pH 7.4, 10 mL) as the receiving solution from 0 to 120 min, and from 120 min onwards 0.5% v/v Tween 20 in acetate buffer (10 mM, pH 4.5, 10 mL) was used as the receiving solution. For PX- and SN38-loaded micelles, the same sampling procedure was followed using 0.5% v/v Tween 80 in PBS (10 mM, pH 7.4, 10 mL) as the receiving solution from 0 to 120 min, and from 120 min onwards 0.5% v/v Tween 80 in acetate buffer (10 mM, pH 4.5, 10 mL) was used as the receiving solution. The concentration of DOX, GP and SN38 in the release media at each time point was determined via UV-vis spectrophotometry at λ_{max} = 479, 380 and 383 nm against calibration curves in the same release media used for the drug release; i.e., PBS or Tween in PBS (pH 7.4; 30, 60, 90 and 120 min samples) and acetate (pH 4.5) or Tween in acetate buffer (pH 4.5) (150–540 min samples) [2]. The concentration of PX in the release media at each time point was determined *via* HPLC. All experiments were repeated in triplicate. Control experiments (free drug) were conducted in a similar manner by measuring the release of DOX, GP, PX and SN38 (0.1 and 0.2 mg/mL) using the same sampling frequency and receiving buffers used for the micelles, however, the free drugs were initially dissolved in PBS (DOX), 0.5% v/v Tween 20 in PBS (GP) or 0.5% v/v Tween 80 in PBS (PX and SN38) at pH 7.4.

Cell viability assay

Prostate (PC-3) and ovarian cancer (SKOV-3 and ES-2) cell lines were used to evaluate the cytotoxicity of blank and OVP-loaded (0.1 and 0.3 mg/mg of copolymer) copolymer micelles with model drugs (0.1 mg/mg of copolymer), using a MTT assay. SKOV-3 and ES-2 cells were grown in McCoy's medium and PC-3 cells in RPMI medium, both supplemented with 1% (v/v) penicillin-streptomycin and 10% (v/v) FBS, at 37 °C in a humidified incubator with 5% CO_2. For the treatment, cells were seeded at a density of 5×10^3 cells/well in 100 μL media followed by incubation at 37 °C for 24 h. Separately, free drug and drug-loaded micelle stock solutions (prepared as previously described) were serially diluted with prewarmed media to provide final micelles concentrations of 500, 250, 125, 62.5 and 31.25 μg/mL, drug concentration of 50, 25, 12.5, 6.25 and 3.13 μg/mL, and OVP concentrations of either 50, 25, 12.5, 6.25 and 3.13 μg/mL (loading of 0.1 mg/mg of copolymer) or 150, 75, 37.5, 18.75 and 9.38 μg/mL (loading of 0.3 mg/mg of copolymer). The diluted solutions were then added to the wells containing the seeded cells (100 μL/well) and the plates were incubated for 48–72 h. The media was carefully removed from the wells and the cells were washed with Dulbecco's phosphate buffered saline (DPBS; 150 μL/well) and then treated with 3-(4,5-dimethylthiazol-2-yl)-2,5-diphenyltetrazolium bromide (MTT) solution in DPBS (0.5 mg/mL, 150 μL/well), followed by incubation at 37 °C for 4 h. After that, MTT solutions were removed from the wells and dimethyl sulfoxide (DMSO) was added (150 μL/well) subsequently and the plates were agitated on an orbital shaker (Ratek,

Australia) for 10 min. The absorbance of each sample was measured at λ = 570 nm using a multimode plate reader. All experiments were repeated in triplicate for two technical repeats. The percentage of cell viability relative to the non-treated cells (negative control) was calculated using the following equation [3]:

$$Cell\ viability\ \% = \left(\frac{Abs_{sample}}{Abs_{control}}\right) X\ 100\%$$

where Abs_{sample} and $Abs_{control}$ represents the absorbance of the treated sample and the absorbance of the control (untreated sample), respectively.

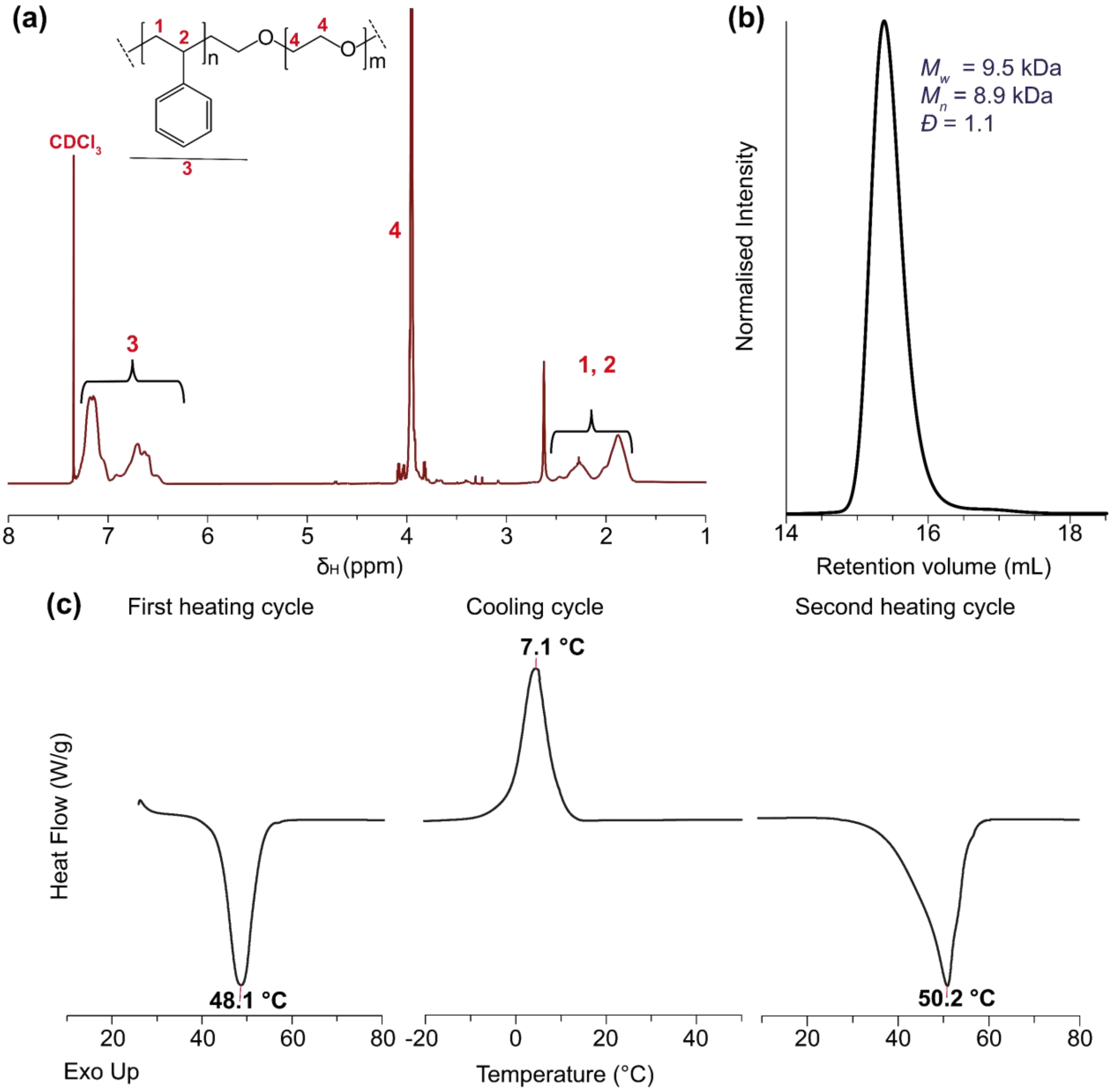

Figure S1: **(a)** ^{1}H NMR spectrum (500 MHz, $CDCl_3$, 25 °C) of the PEG_5PS_5 block copolymer. Comparison of the integral values of the resonances corresponding to the PEG (δ_H 3.65 ppm) and PS (δ_H 6.4–7.2 ppm) repeat units was used to determine the $M_{n,\mathrm{NMR}}$ of the copolymer (10.7 kDa). **(b)** Normalized gel permeation chromatography differential refractive index (GPC DRI) chromatogram of the PEG_5PS_5 block copolymer; molecular weight characteristics determined with reference to a conventional column calibration with narrow molecular weight PS standards. **(c)** Differential scanning calorimetry (DSC) thermograms of the PEG_5PS_5 copolymer showing the first heating, first cooling and second heating profiles (ramp rate 10 °C/min).

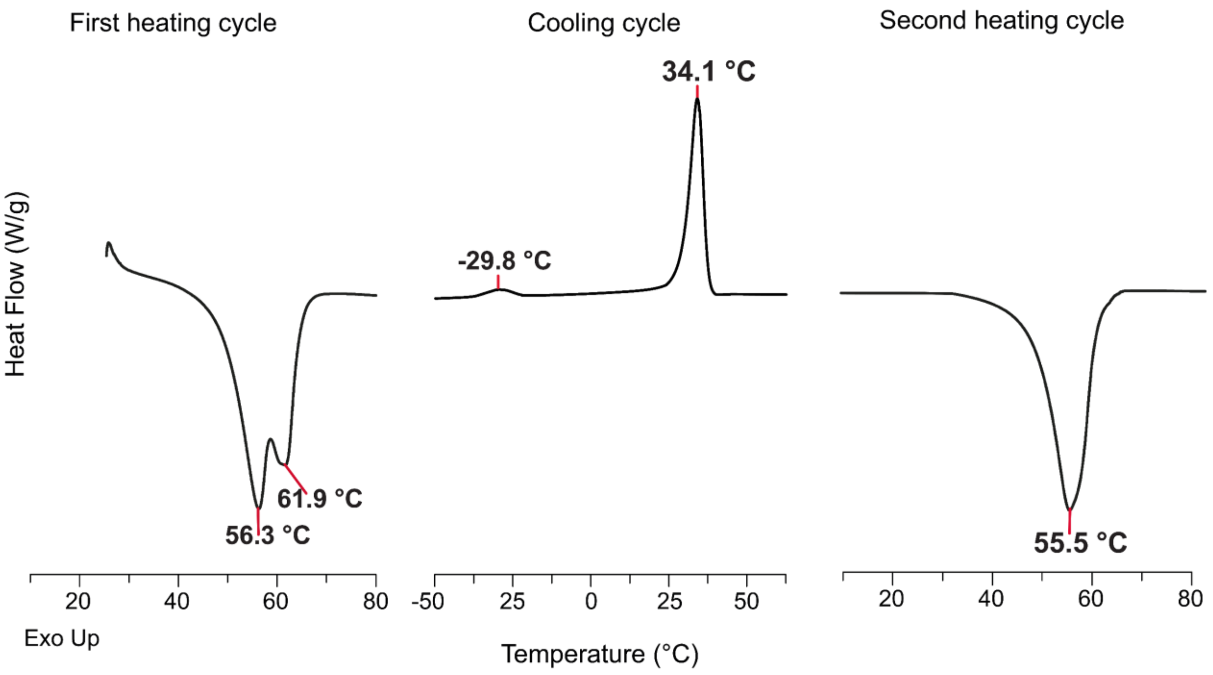

Figure S2: DSC thermograms of the PEG$_{14}$PS$_{12}$ copolymer showing the first heating, first cooling and second heating profiles (ramp rate 10 °C/min).

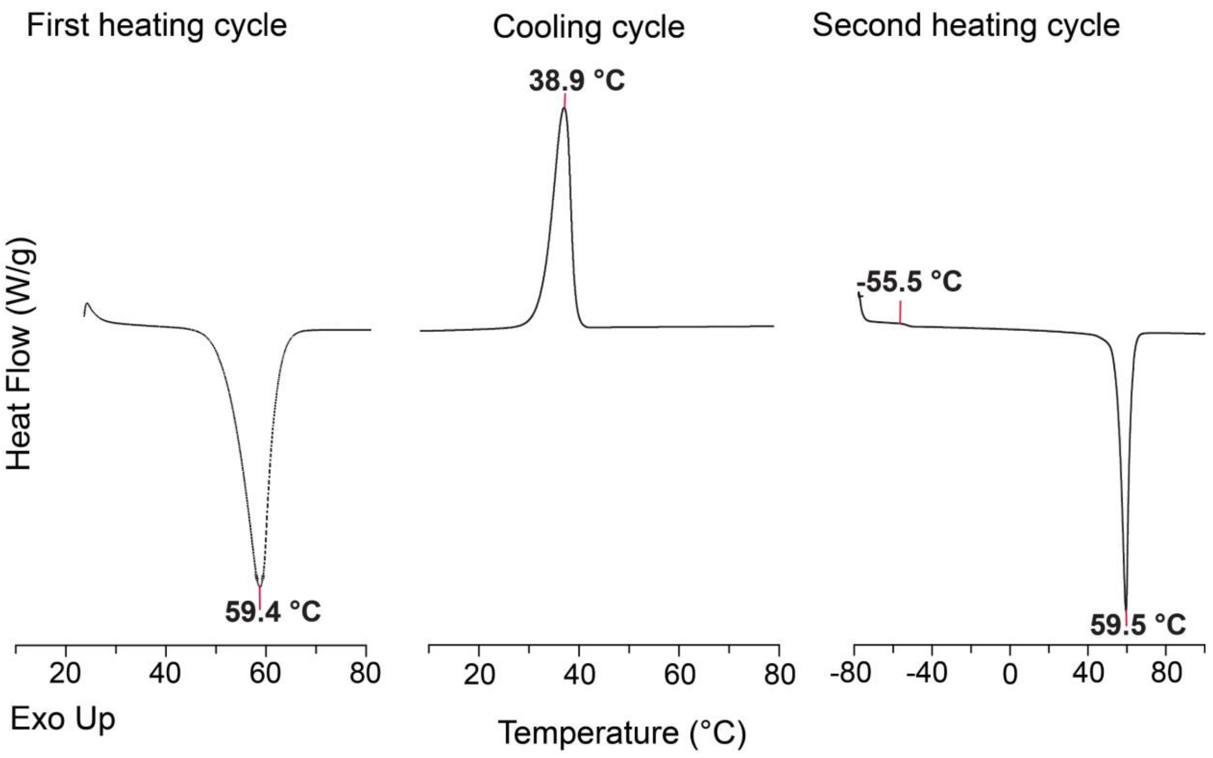

Figure S3: DSC thermograms of the PEG$_{10}$PDL$_{10}$ copolymer showing the first heating, first cooling and second heating profiles (ramp rate 10 °C/min).

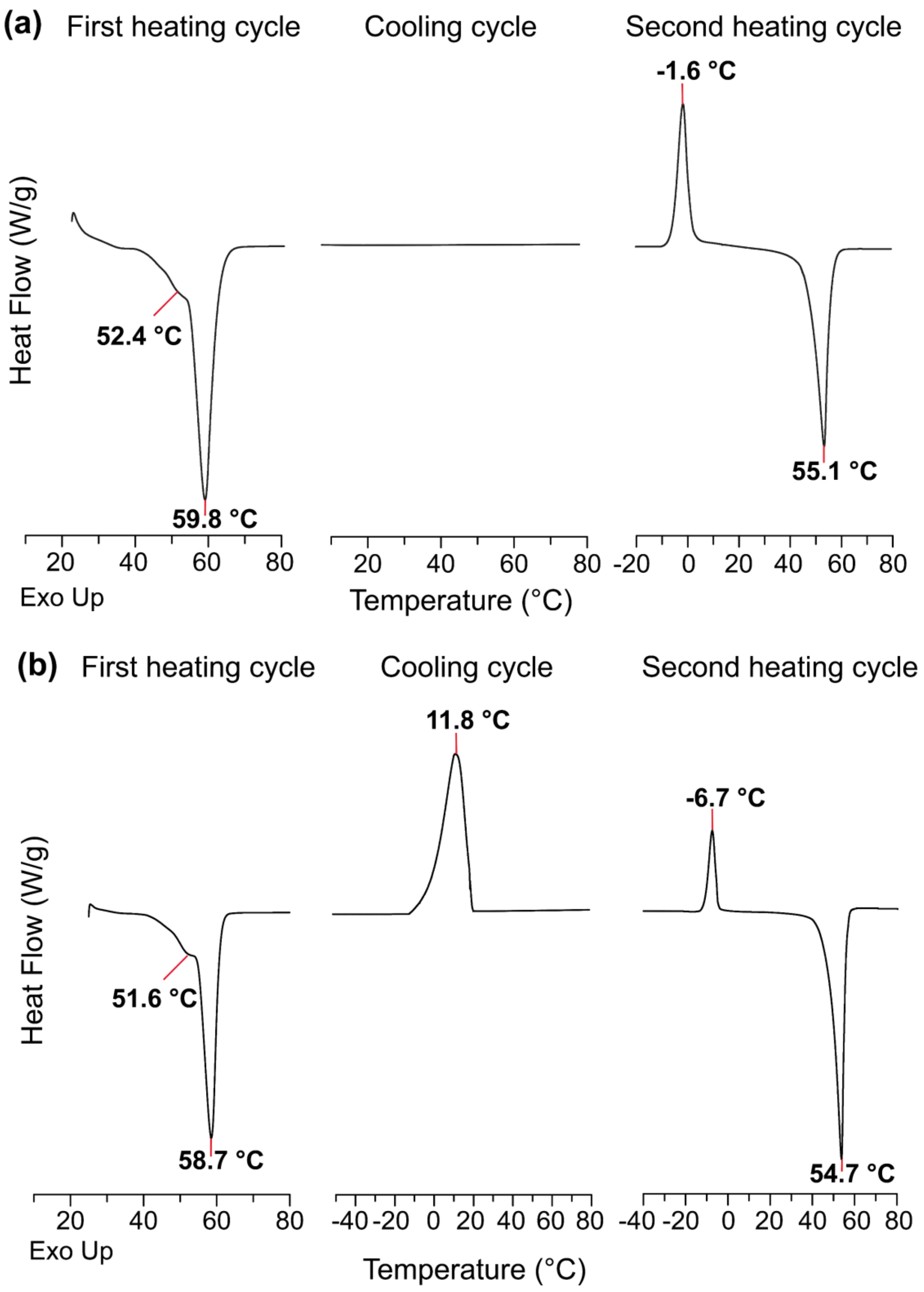

Figure S4: DSC thermograms of the $PEG_{10}PLA_{10}$ copolymer showing the first heating, first cooling and second heating profiles at ramp rates of **(a)** 10 and **(b)** 5 °C/min.

Table S1: Thermal properties of PEG_5PS_5, $PEG_{14}PS_{12}$ $PEG_{10}PDL_{10}$ and $PEG_{10}PLA_{10}$ copolymers determined *via* DSC (n = 3). Thermal transitions (melting temperature (T_m) and temperature of crystallisation (T_{cryst})) and their associated enthalpy changes (melting enthalpy (ΔH_m) and crystallization enthalpy (ΔH_{cryst})) determined from the second heating and first cooling cycles, respectively.

Copolymer	T_m (°C)	T_m peak max (°C)	ΔH_m (J/g)	T_{cryst} (°C)	T_{cryst} peak max (°C)	ΔH_{cryst} (J/g)	X_c (%)[c]
**PEG_5PS_5[a]**	42.3 ± 0.3	50.2 ± 0.3	76.9 ± 1.6	11.4 ± 0.2	7.1 ± 0.2	69.3 ± 2.1	38
**$PEG_{14}PS_{12}$[a]**	48.5 ± 0.7	55.5 ± 0.1	78.3 ± 1.8	37.5 ± 0.6	34.1 ± 0.7	75.8 ± 3.4	38
				-29.8 ± 0.1	-22.6 ± 0.1	3.5 ± 0.2	2
**$PEG_{10}PDL_{10}$[a]**	54.8 ± 0.4	59.5 ± 0.2	107.7 ± 2.8	41.1 ± 0.5	38.9 ± 0.5	105.1 ± 3.3	53
**$PEG_{10}PLA_{10}$[a]**	48.9 ± 0.6	54.7 ± 0.3	81 ± 2.1	-	-	-	40
	-5.6 ± 0.2	-1.6 ± 0.1	35.4 ± 0.7				
**$PEG_{10}PLA_{10}$[b]**	48.8 ± 0.5	54.5 ± 0.3	78.0 ± 1.9	19.6 ± 0.3	11.8 ± 0.2	13.6 ± 1.3	38
	-9.9 ± 0.1	-6.8 ± 0.1	22.2 ± 0.5				

[a] Determined using a ramp rate of 10 °C/min. [b] Determined using a ramp rate of 5 °C/min. [c] The degree of crystallinity (X_c) of the PEG block was calculated from the equation: $X_{DSC} \approx (\Delta H_m/\Delta H_u)$*100%, where ΔH_u is the enthalpy of fusion per crystalline repeat unit and has been reported to be 205 J/g for 100% crystalline PEG [4].

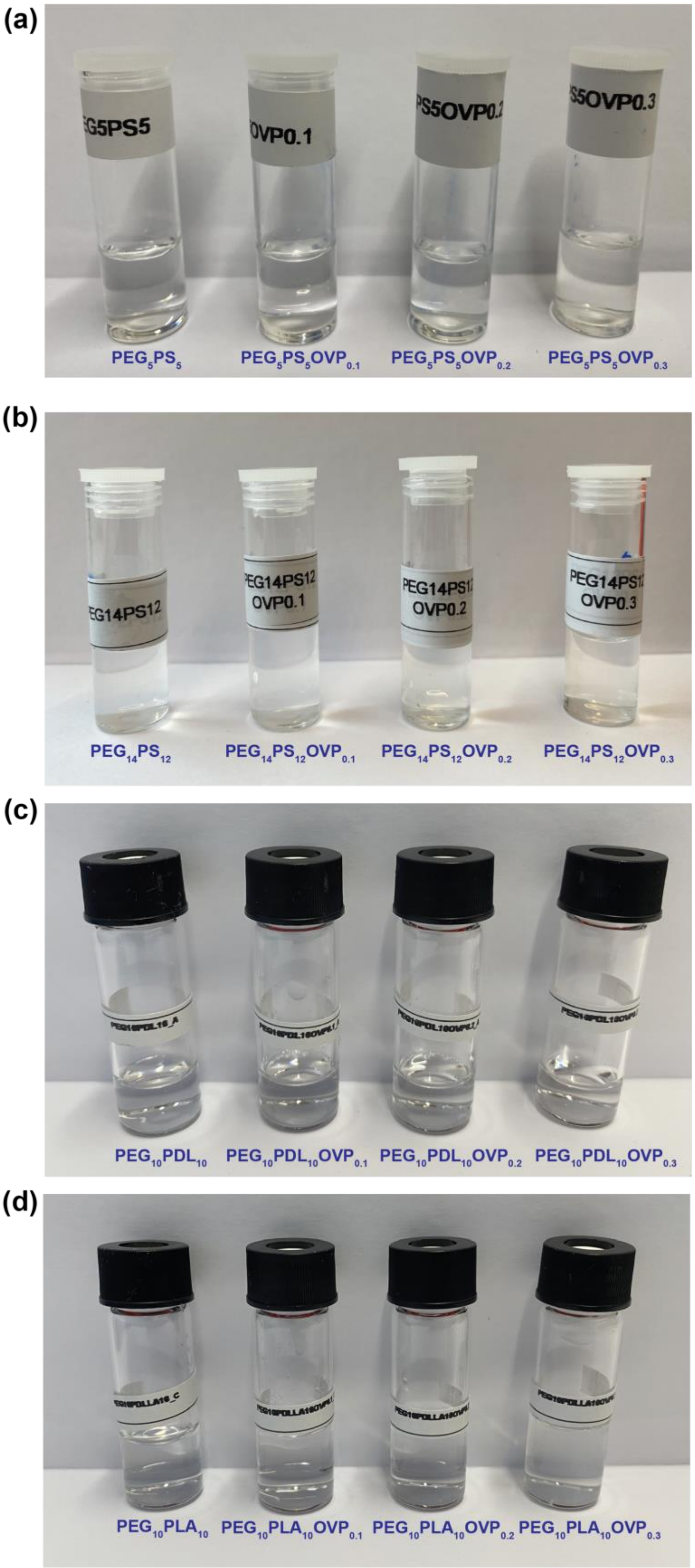

Figure S5: Images of blank and OVP-loaded (0.1, 0.2 and 0.3 mg/mg of copolymer) (**a**) PEG_5PS_5, (**b**) $PEG_{14}PS_{12}$, (**c**) $PEG_{10}PDL_{10}$ and (**d**) $PEG_{10}PLA_{10}$ micelles in PBS (pH 7.4).

Table S2: Concentration and volume of stock solutions combined to prepare copolymer solutions in PBS for the OVP encapsulation EE% analysis for OVP-loaded (0.1, 0.2 and 0.3 mg/mg of copolymer) PEG_xHB_y diblock micelles. Samples were prepared in triplicate.

Micelle composition[a]	Solvent used for copolymer and OVP stock solutions	Conc. of copolymer stock solution (mg/L)	Volume of copolymer stock solution (µL)	OVP mass fraction (mg/mg of copolymer)	Conc. of OVP stock solution (mg/L)	Volume of OVP stock solution (µL)	Volume of PBS added (10 mM, pH 7.4) (µL)
$PEG_xHB_yOVP_{0.1}$	Acetone	1000	1000	0.1	1000	100	1000
$PEG_xHB_yOVP_{0.2}$	Acetone	1000	1000	0.2	1000	200	1000
$PEG_xHB_yOVP_{0.3}$	Acetone	1000	1000	0.3	1000	300	1000

[a]Micelle solutions were prepared in PBS *via* the solvent evaporation approach. HB stands for hydrophobic block and represents PS, PDL or PLA.

Table S3: Concentration and volume of stock solutions combined to prepare copolymer solutions in PBS (10 mM, pH 7.4) for the determination of the CMC of blank and OVP-loaded (0.1, 0.2 and 0.3 mg/mg of copolymer) PEG_5PS_5, $PEG_{14}PS_{12}$, $PEG_{10}PDL_{10}$ and $PEG_{10}PLA_{10}$ micelles.

Conc. of copolymer in PBS (mg/L)[a]	Volume of pyrene stock solution (μL)[b]	Conc. of copolymer stock solution (mg/L)[c]	Volume of copolymer stock solution (μL)	OVP mass fraction/mg of copolymer	Conc. of OVP stock solution (mg/L)[c]	Volume of OVP stock solution (μL)	Volume of acetone (μL)	Volume of PBS (10 mM, pH 7.4) (μL)
500	20	2000	500	0	-	-	1480	2000
				0.1	2000	50	1430	
				0.2	2000	100	1380	
				0.3	2000	150	1330	
100	20	2000	100	0	-	-	1880	2000
				0.1	2000	10	1870	
				0.2	2000	20	1860	
				0.3	2000	30	1850	
50.0	20	1000	100	0	-	-	1880	2000
				0.1	1000	10	1870	
				0.2	1000	20	1860	
				0.3	1000	30	1850	
10.0	20	1000	20	0	-	-	1960	2000
				0.1	1000	2	1958	
				0.2	1000	4	1956	
				0.3	1000	6	1954	
5.00	20	1000	10	0	-	-	1970	2000
				0.1	100	10	1960	
				0.2	100	20	1950	
				0.3	100	30	1940	
2.50	20	100	50	0	-	-	1930	2000
				0.1	100	5	1925	
				0.2	100	10	1920	
				0.3	100	15	1915	

Table continued on next page

1.00	20	100	20	0	-	-	1960	2000
				0.1	10	20	1940	
				0.2	10	40	1920	
				0.3	10	60	1900	
0.75	20	100	15	0	-	-	1965	2000
				0.1	10	15	1950	
				0.2	10	30	1935	
				0.3	10	45	1920	
0.50	20	100	10	0	-	-	1970	2000
				0.1	10	10	1960	
				0.2	10	20	1950	
				0.3	10	30	1940	
0.25	20	10	50	0	-	-	1930	2000
				0.1	10	5	1925	
				0.2	10	10	1920	
				0.3	10	15	1915	
0.10	20	10	20	0	-	-	1960	2000
				0.1	10	2	1958	
				0.2	10	4	1956	
				0.3	10	6	1954	

[a]Copolymer solutions were prepared in PBS via the solvent evaporation approach with a constant pyrene concentration of 6×10^{-7} M. [b] A 6×10^{-5} M pyrene stock solution in acetone was used to prepare all solutions. [c]All stock solutions were prepared in acetone.

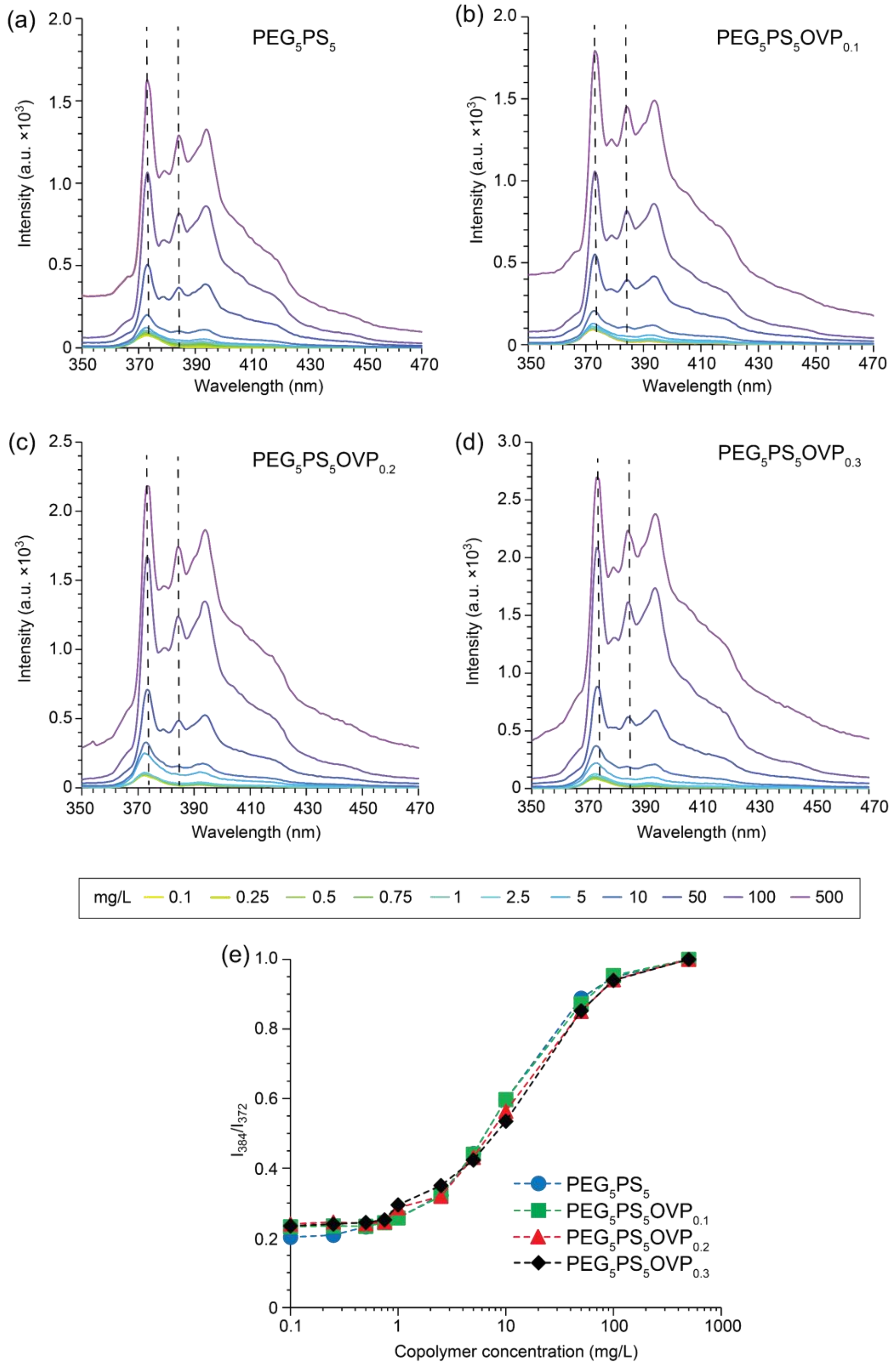

Figure S6: Pyrene (6 x 10^{-7} M) fluorescent emission spectra (λ_{ex} = 334 nm) for **(a)** PEG_5PS_5, **(b)** $PEG_5PS_5OVP_{0.1}$, **(c)** $PEG_5PS_5OVP_{0.2}$ and **(d)** $PEG_5PS_5OVP_{0.3}$ micelles at various copolymer concentrations. **(e)** Ratiometric intensity of the pyrene emission (I_{384}/I_{372}) as a function of copolymer concentration for blank and OVP-loaded PEG_5PS_5 micelles in PBS (10 mM, pH 7.4). Only symbols represent the experimental data; the (dashed/dotted/solid) lines are guides to the eyes.

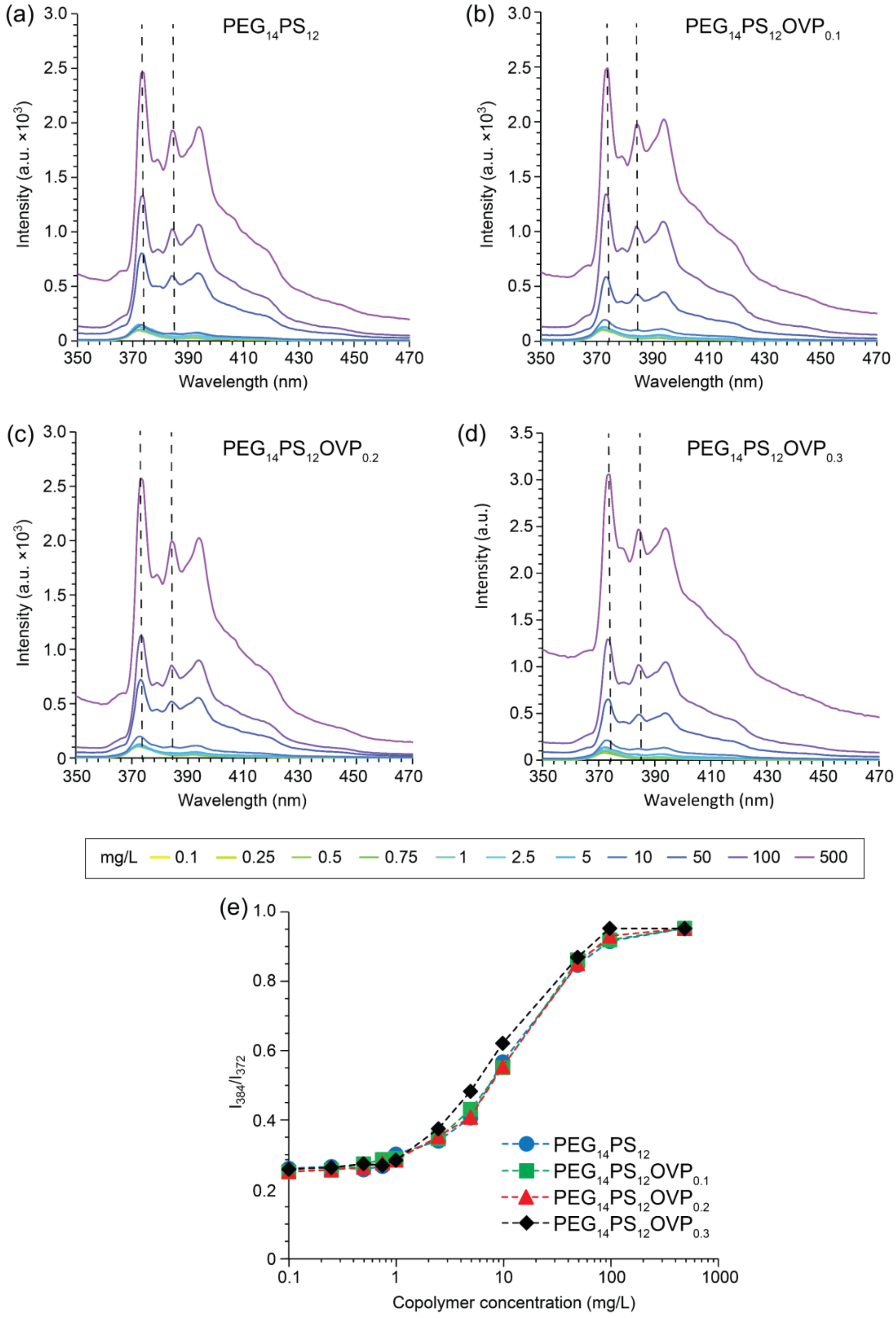

Figure S7: Pyrene (6 x 10^{-7} M) fluorescent emission spectra (λ_{ex} = 334 nm) for **(a)** $PEG_{14}PS_{12}$, **(b)** $PEG_{14}PS_{12}OVP_{0.1}$, **(c)** $PEG_{14}PS_{12}OVP_{0.2}$ and **(d)** $PEG_{14}PS_{12}OVP_{0.3}$ micelles at various copolymer concentrations. **(e)** Ratiometric intensity of the pyrene emission (I_{384}/I_{372}) as a function of copolymer concentration for blank and OVP-loaded $PEG_{14}PS_{12}$ micelles in PBS (10 mM, pH 7.4). Only symbols represent the experimental data; the (dashed/dotted/solid) lines are guides to the eyes.

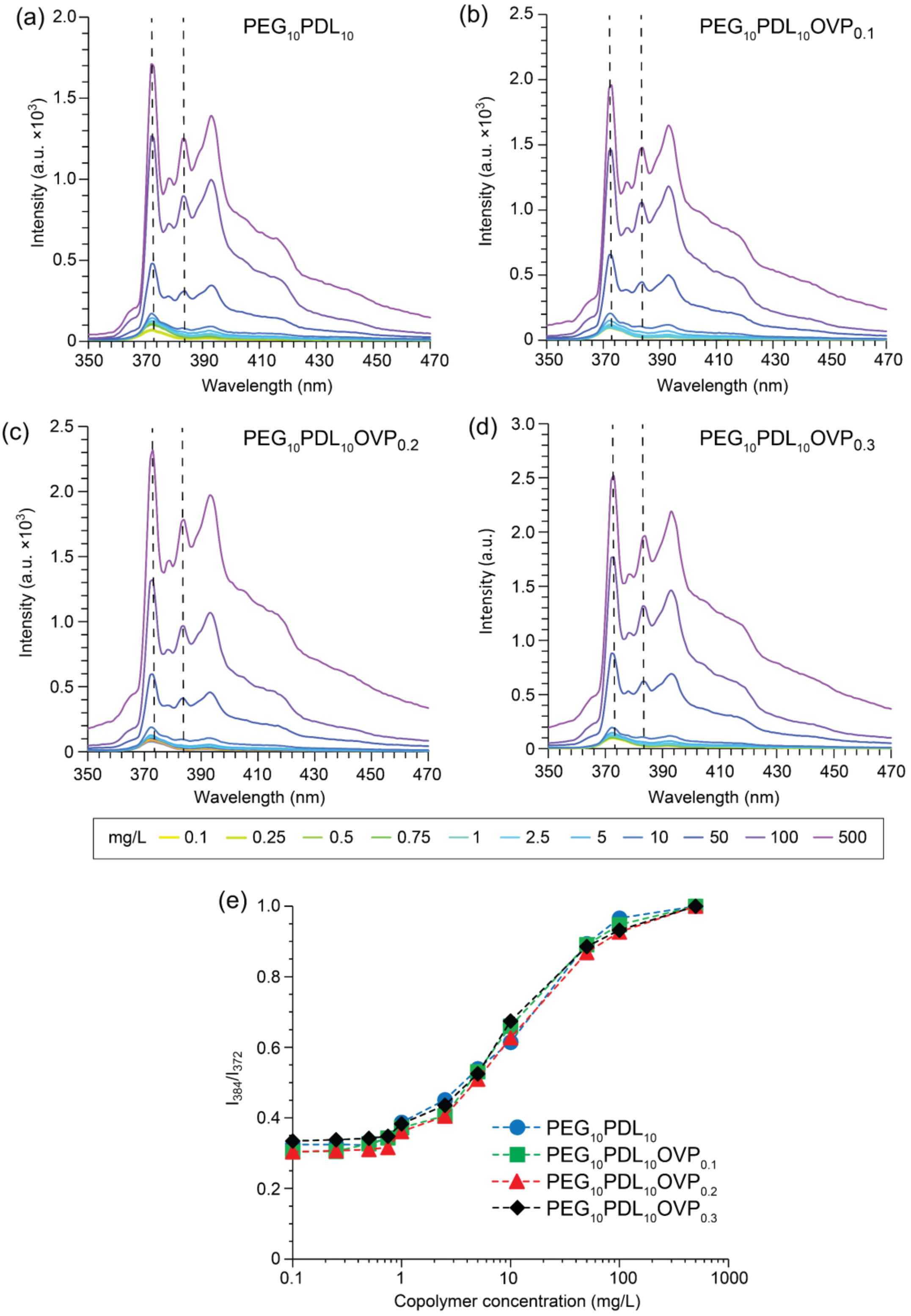

Figure S8: Pyrene (6 x 10^{-7} M) fluorescent emission spectra (λ_{ex} = 334 nm) for **(a)** PEG$_{10}$PDL$_{10}$, **(b)** PEG$_{10}$PDL$_{10}$OVP$_{0.1}$, **(c)** PEG$_{10}$PDL$_{10}$OVP$_{0.2}$ and **(d)** PEG$_{10}$PDL$_{10}$OVP$_{0.3}$ micelles at various copolymer concentrations. **(e)** Ratiometric intensity of the pyrene emission (I_{384}/I_{372}) as a function of copolymer concentration for blank and OVP-loaded PEG$_{10}$PDL$_{10}$ micelles in PBS (10 mM, pH 7.4). Only symbols represent the experimental data; the (dashed/dotted/solid) lines are guides to the eyes.

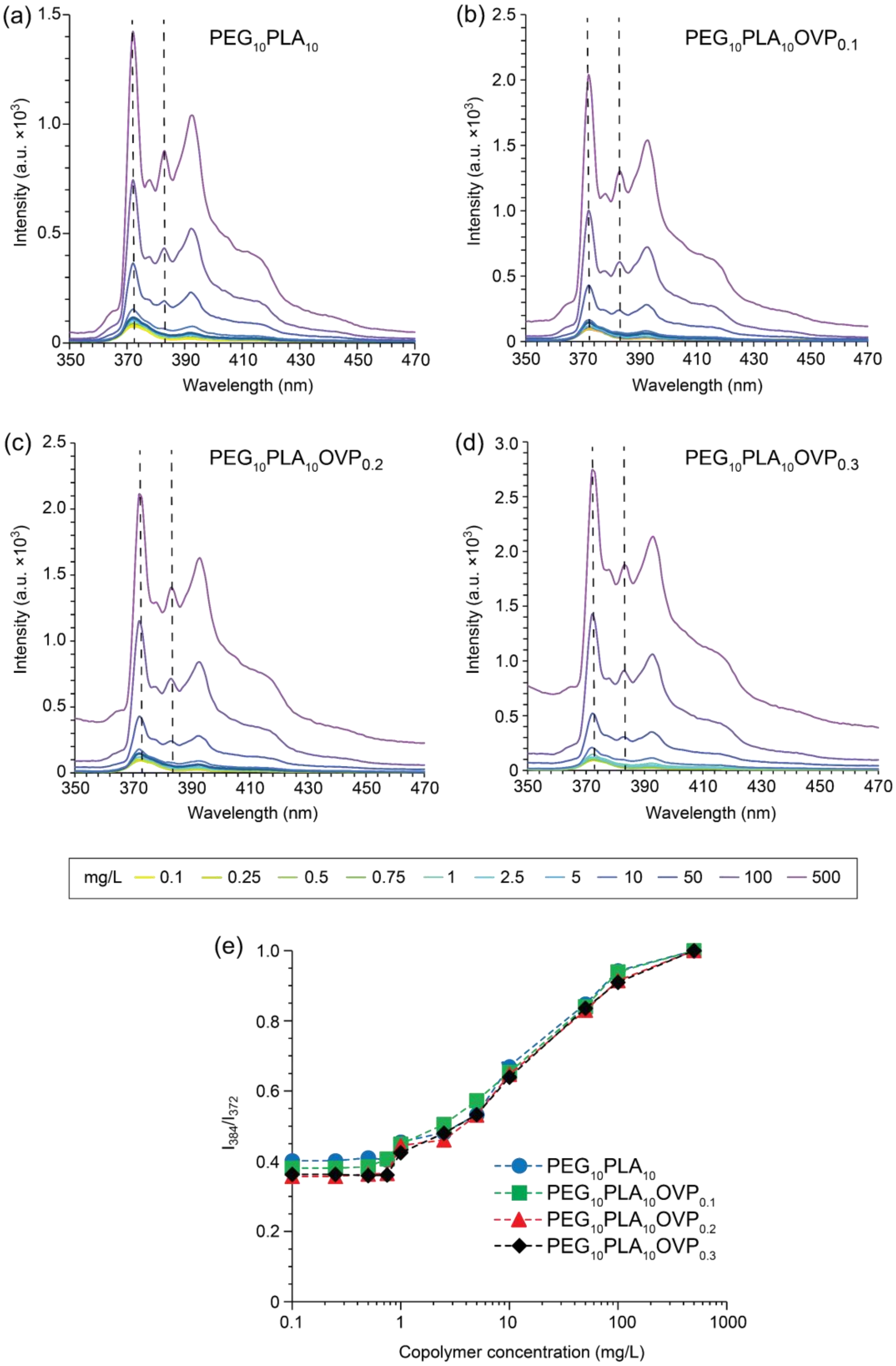

Figure S9: Pyrene (6 x 10^{-7} M) fluorescent emission spectra (λ_{ex} = 334 nm) for **(a)** $PEG_{10}PLA_{10}$, **(b)** $PEG_{10}PLA_{10}OVP_{0.1}$, **(c)** $PEG_{10}PLA_{10}OVP_{0.2}$ and **(d)** $PEG_{10}PLA_{10}OVP_{0.3}$ micelles at various copolymer concentrations. **(e)** Ratiometric intensity of the pyrene emission (I_{384}/I_{372}) as a function of copolymer concentration for blank and OVP-loaded $PEG_{10}PLA_{10}$ micelles in PBS (10 mM, pH 7.4). Only symbols represent the experimental data; the (dashed/dotted/solid) lines are guides to the eyes.

Table S4: pH adjustment of blank and OVP-loaded (0.1, 0.2 and 0.3 mg/mg of copolymer) PEG_5PS_5 micelles initially prepared in PBS-d (10 mM, pH 7.3) at a copolymer concentration of 0.5 mg/mL.

Micelle	Stock solutions	Volume stock solution (μL)[a]				
		pH 7.3	pH 6.5	pH 5.5	pH 4.6	pH 3.7
PEG_5PS_5	PEG_5PS_5 (1 mg/mL) in acetone	1000				
	OVP (1 mg/mL) in acetone	0.0				
	DCl (1 M) in D_2O	0.0	9.8	16.1	19.6	21.9
	Acetone	1000				
	PBS-d (10 mM, pH 7.3)	2000				
$PEG_5PS_5OVP_{0.1}$	PEG_5PS_5 (1 mg/mL) in acetone	1000				
	OVP (1 mg/mL) in acetone	100				
	DCl (1 M) in D_2O	0.0	10.0	16.1	19.7	22.0
	Acetone	900				
	PBS-d (10 mM, pH 7.3)	2000				
$PEG_5PS_5OVP_{0.2}$	PEG_5PS_5 (1 mg/mL) in acetone	1000				
	OVP (1 mg/mL) in acetone	200				
	DCl (1 M) in D_2O	0.0	10.1	16.2	19.7	22.1
	Acetone	800				
	PBS-d (10 mM, pH 7.3)	2000				
$PEG_5PS_5OVP_{0.3}$	PEG_5PS_5 (1 mg/mL) in acetone	1000				
	OVP (1 mg/mL) in acetone	300				
	DCl (1 M) in D_2O	0.0	10.1	16.2	19.8	22.1
	Acetone	700				
	PBS-d (10 mM, pH 7.3)	2000				

Table S5: pH adjustment of blank and OVP-loaded (0.1, 0.2 and 0.3 mg/mg of copolymer) $PEG_{14}PS_{12}$ micelles initially prepared in PBS-d (10 mM, pH 7.3) at a copolymer concentration of 0.5 mg/mL.

**Micelle**	**Stock solutions**	**Volume stock solution (μL)**[a] pH 7.3	pH 6.5	pH 5.5	pH 4.6	pH 3.7
$PEG_{14}PS_{12}$	$PEG_{14}PS_{12}$ (1 mg/mL) in acetone	1000				
	OVP (1 mg/mL) in acetone	0.0				
	DCl (1 M) in D_2O	0.0	10.1	16.1	19.6	22.0
	Acetone	1000				
	PBS-d (10 mM, pH 7.3)	2000				
$PEG_{14}PS_{12}OVP_{0.1}$	$PEG_{14}PS_{12}$ (1 mg/mL) in acetone	1000				
	OVP (1 mg/mL) in acetone	100				
	DCl (1 M) in D_2O	0.0	10.1	16.0	19.6	21.9
	Acetone	900				
	PBS-d (10 mM, pH 7.3)	2000				
$PEG_{14}PS_{12}OVP_{0.2}$	$PEG_{14}PS_{12}$ (1 mg/mL) in acetone	1000				
	OVP (1 mg/mL) in acetone	200				
	DCl (1 M) in D_2O	0.0	10.2	16.1	19.7	22.1
	Acetone	800				
	PBS-d (10 mM, pH 7.3)	2000				
$PEG_{14}PS_{12}OVP_{0.3}$	$PEG_{14}PS_{12}$ (1 mg/mL) in acetone	1000				
	OVP (1 mg/mL) in acetone	300				
	DCl (1 M) in D_2O	0.0	10.2	16.2	19.8	22.2
	Acetone	700				
	PBS-d (10 mM, pH 7.3)	2000				

Table S6: pH adjustment of blank and OVP-loaded (0.1, 0.2 and 0.3 mg/mg of copolymer) $PEG_{10}PDL_{10}$ micelles initially prepared in PBS-d (10 mM, pH 7.3) at a copolymer concentration of 0.5 mg/mL.

Micelle	Stock solutions	Volume stock solution (µL)[a]				
		pH 7.3	pH 6.5	pH 5.5	pH 4.6	pH 3.7
$PEG_{10}PDL_{10}$	PEG-PLA (1 mg/mL) in acetone	1000				
	OVP (1 mg/mL) in acetone	0.0				
	DCl (1 M) in D_2O	0.0	10.0	16.0	19.6	22.1
	Acetone	1000				
	PBS-d (10 mM, pH 7.3)	2000				
$PEG_{10}PDL_{10}OVP_{0.1}$	PEG-PLA (1 mg/mL) in acetone	1000				
	OVP (1 mg/mL) in acetone	100				
	DCl (1 M) in D_2O	0.0	10.1	16.1	19.7	22.2
	Acetone	900				
	PBS-d (10 mM, pH 7.3)	2000				
$PEG_{10}PDL_{10}OVP_{0.2}$	PEG-PLA (1 mg/mL) in acetone	1000				
	OVP (1 mg/mL) in acetone	200				
	DCl (1 M) in D_2O	0.0	10.2	16.2	19.8	22.2
	Acetone	800				
	PBS-d (10 mM, pH 7.3)	2000				
$PEG_{10}PDL_{10}OVP_{0.3}$	PEG-PLA (1 mg/mL) in acetone	1000				
	OVP (1 mg/mL) in acetone	300				
	DCl (1 M) in D_2O	0	10.2	16.2	19.8	22.2
	Acetone	700				
	PBS-d (10 mM, pH 7.3)	2000				

Table S7: pH adjustment of blank and OVP-loaded (0.1, 0.2 and 0.3 mg/mg of copolymer) $PEG_{10}PLA_{10}$ micelles initially prepared in PBS-d (10 mM, pH 7.3) at a copolymer concentration of 0.5 mg/mL.

Micelle	Stock solutions	Volume stock solution (μL)[a]				
		pH 7.3	pH 6.5	pH 5.5	pH 4.6	pH 3.7
$PEG_{10}PLA_{10}$	PEG-PLA (1 mg/mL) in acetone	1000				
	OVP (1 mg/mL) in acetone	0.0				
	DCl (1 M) in D_2O	0.0	10.0	16.0	19.6	22.0
	Acetone	1000				
	PBS-d (10 mM, pH 7.3)	2000				
$PEG_{10}PLA_{10}OVP_{0.1}$	PEG-PLA (1 mg/mL) in acetone	1000				
	OVP (1 mg/mL) in acetone	100				
	DCl (1 M) in D_2O	0.0	10.1	16.1	19.6	22.1
	Acetone	900				
	PBS-d (10 mM, pH 7.3)	2000				
$PEG_{10}PLA_{10}OVP_{0.2}$	PEG-PLA (1 mg/mL) in acetone	1000				
	OVP (1 mg/mL) in acetone	200				
	DCl (1 M) in D_2O	0.0	10.1	16.1	19.7	22.1
	Acetone	800				
	PBS-d (10 mM, pH 7.3)	2000				
$PEG_{10}PLA_{10}OVP_{0.3}$	PEG-PLA (1 mg/mL) in acetone	1000				
	OVP (1 mg/mL) in acetone	300				
	DCl (1 M) in D_2O	0.0	10.2	16.2	19.8	22.1
	Acetone	700				
	PBS-d (10 mM, pH 7.3)	2000				

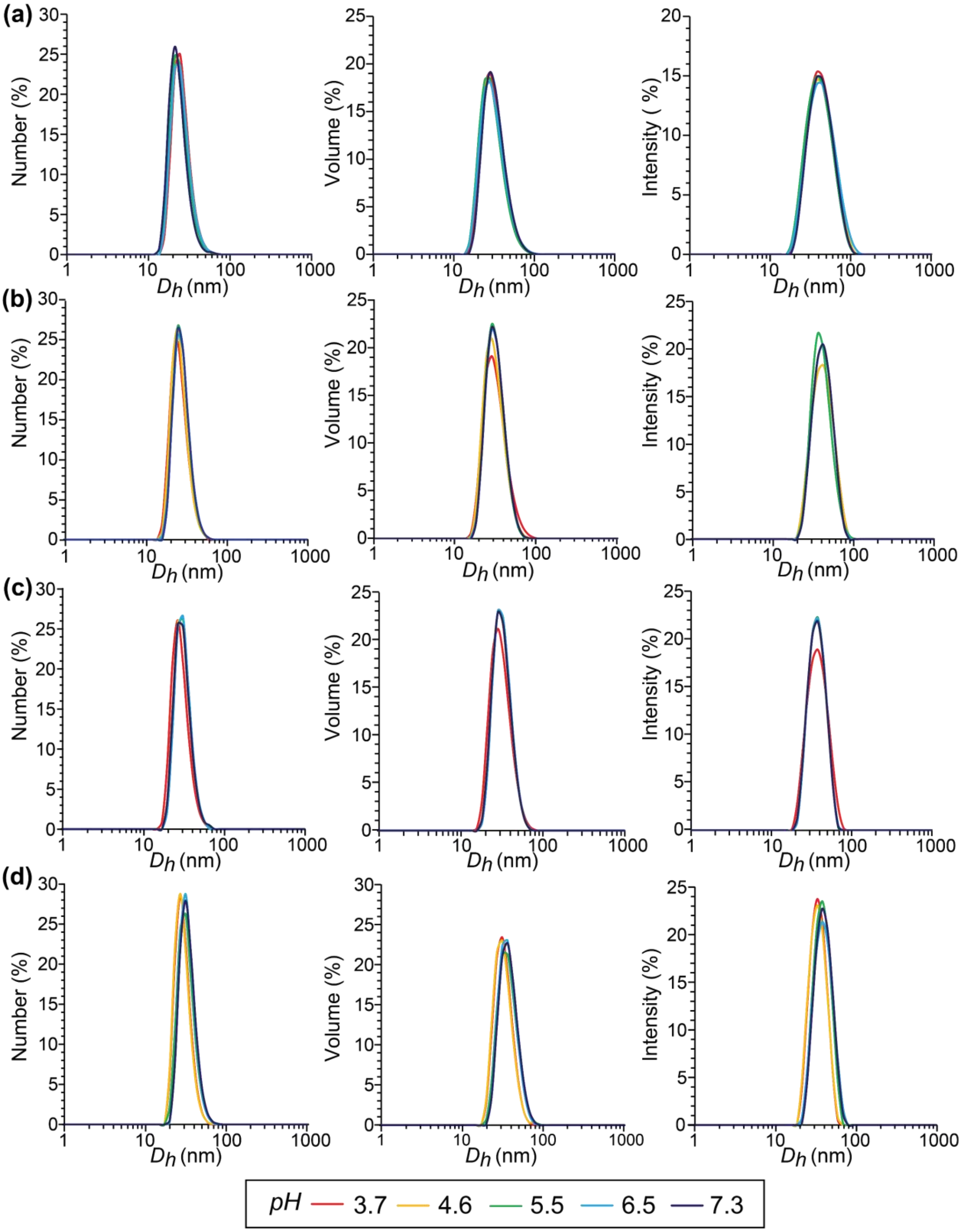

Figure S10: Representative number, volume and intensity PSDs for (**a**) PEG_5PS_5, (**b**) $PEG_5PS_5OVP_{0.1}$, (**c**) $PEG_5PS_5OVP_{0.2}$ and (**d**) $PEG_5PS_5OVP_{0.3}$ micelles as a function of pH, as measured by DLS.

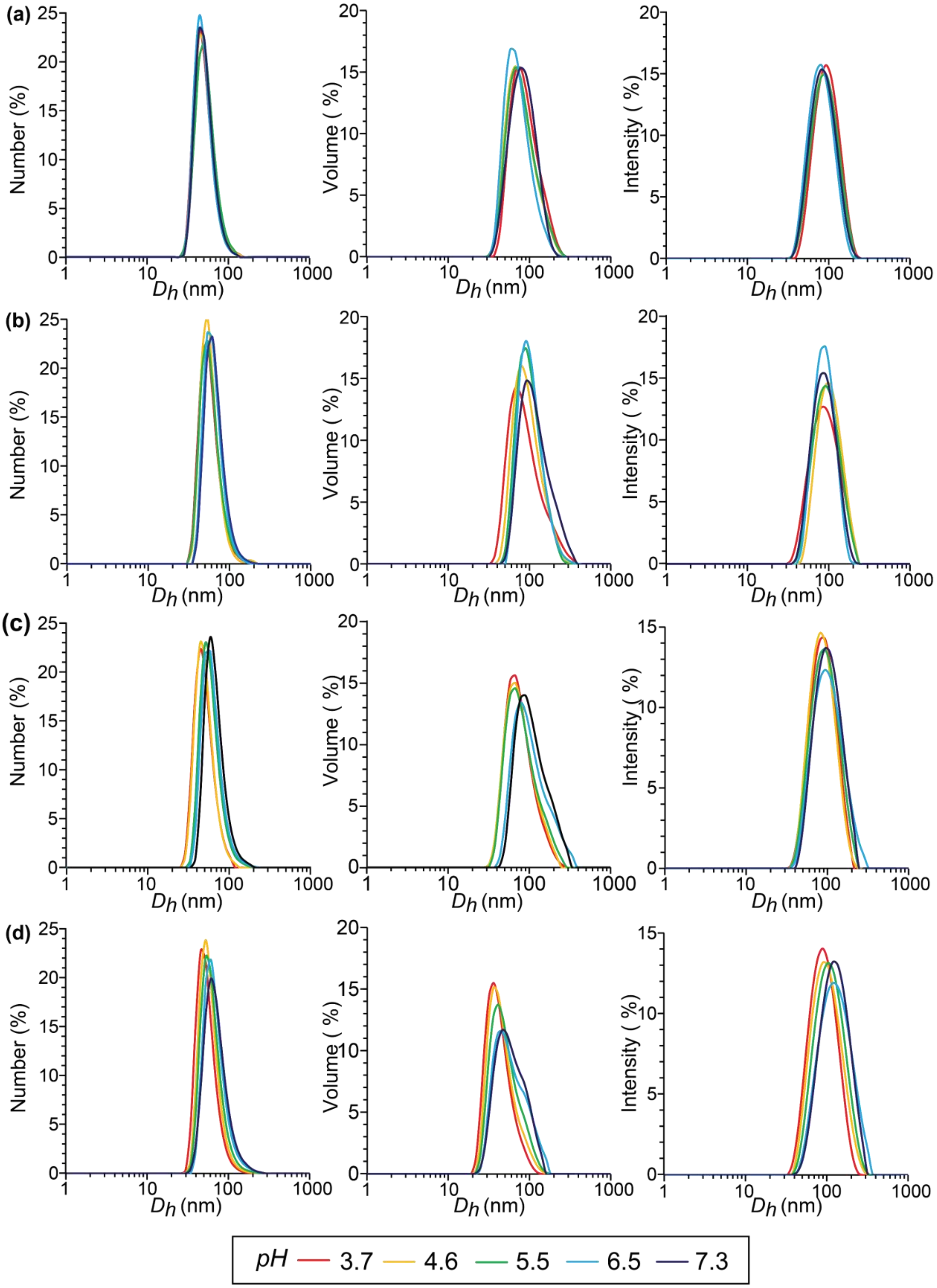

Figure S11: Representative number, volume and intensity PSDs for **(a)** $PEG_{14}PS_{12}$, **(b)** $PEG_{14}PS_{12}OVP_{0.1}$, **(c)** $PEG_{14}PS_{12}OVP_{0.2}$ and **(d)** $PEG_{14}PS_{12}OVP_{0.3}$ micelles as a function of pH, as measured by DLS.

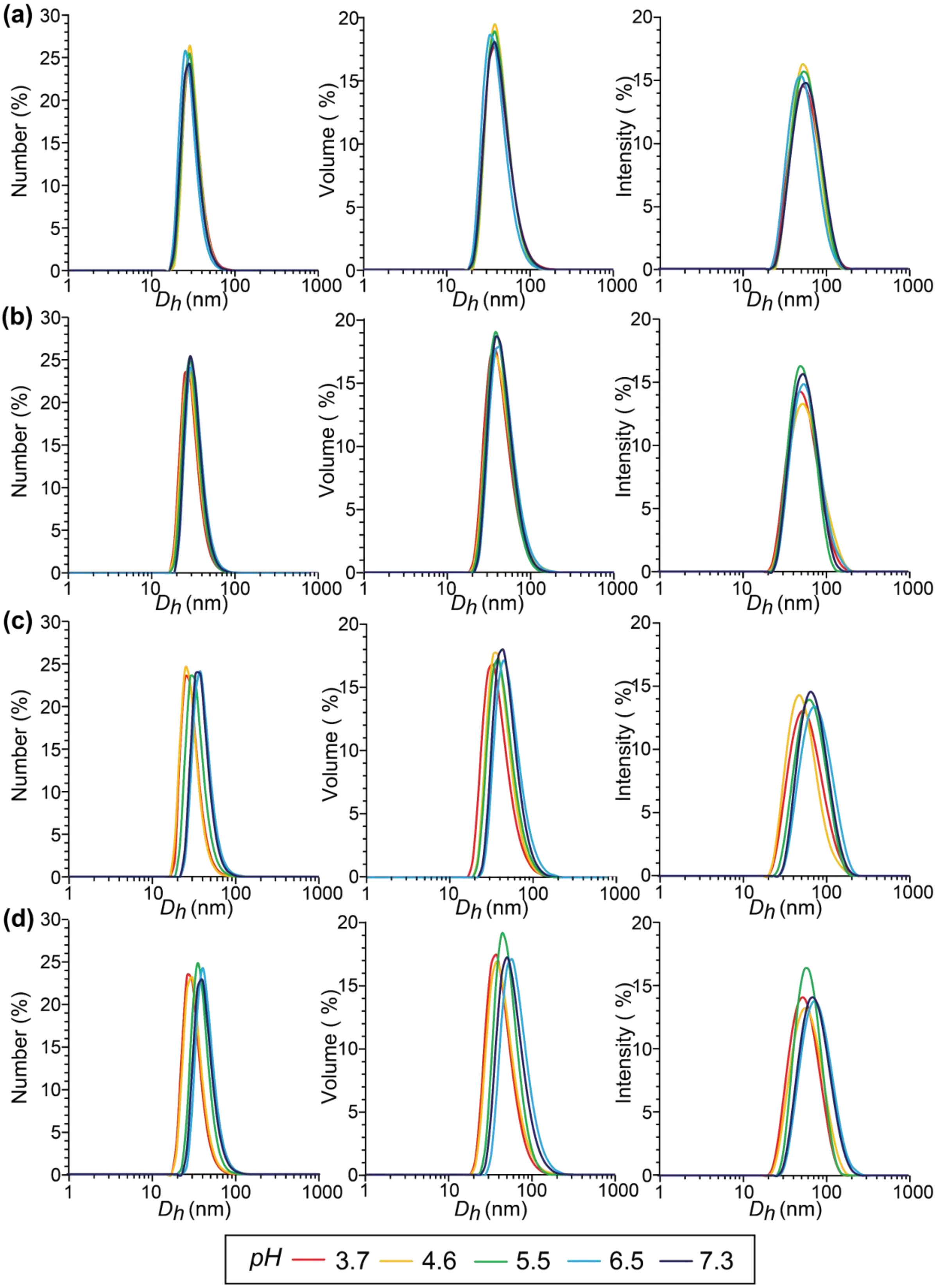

Figure S12: Representative number, volume and intensity PSDs for (**a**) $PEG_{10}PDL_{10}$, (**b**) $PEG_{10}PDL_{10}OVP_{0.1}$, (**c**) $PEG_{10}PDL_{10}OVP_{0.2}$ and (**d**) $PEG_{10}PDL_{10}OVP_{0.3}$ micelles as a function of pH, as measured by DLS.

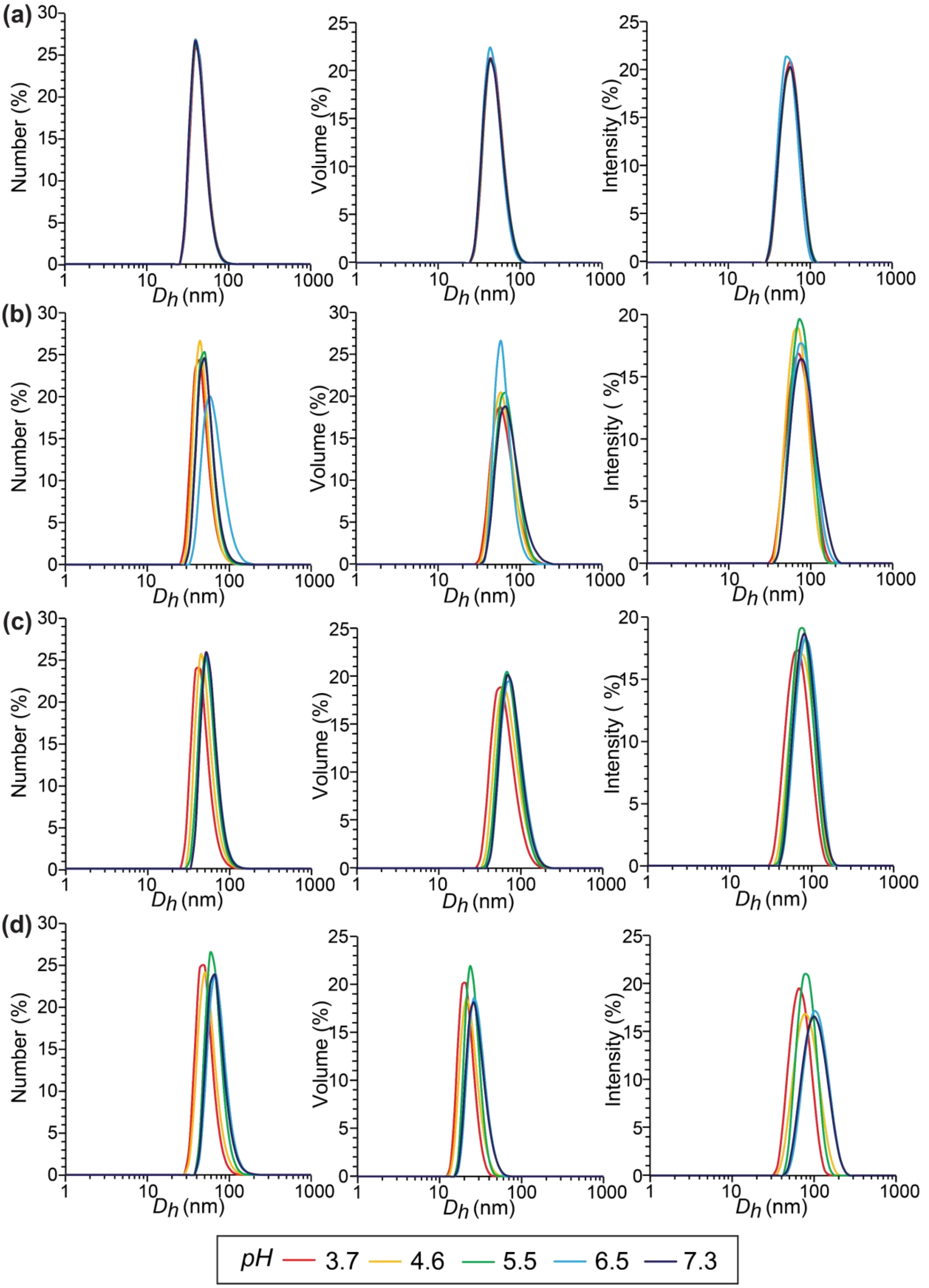

Figure S13: Representative number, volume and intensity PSDs for (**a**) PEG$_{10}$PLA$_{10}$, (**b**) PEG$_{10}$PLA$_{10}$OVP$_{0.1}$, (**c**) PEG$_{10}$PLA$_{10}$OVP$_{0.2}$ and (**d**) PEG$_{10}$PLA$_{10}$OVP$_{0.3}$ micelles as a function of pH, as measured by DLS.

Table S8: PDI values of blank and OVP-loaded (0.1, 0.2 and 0.3 mg/mg of copolymer) PEG_5PS_5, $PEG_{14}PS_{12}$, $PEG_{10}PDL_{10}$ and $PEG_{10}PLA_{10}$ micelles as a function of pH, as determined by DLS. All experiments were conducted in triplicate.

**Micelles**	**pH 7.3 (PDI ± SD)**	**pH 6.5 (PDI ± SD)**	**pH 5.5 (PDI ± SD)**	**pH 4.6 (PDI ± SD)**	**pH 3.7 (PDI ± SD)**
PEG_5PS_5	0.13 ± 0.02	0.13 ± 0.01	0.14 ± 0.02	0.14± 0.01	0.14 ± 0.01
$PEG_5PS_5OVP_{0.1}$	0.15 ± 0.01	0.15 ± 0.02	0.15 ± 0.02	0.13 ± 0.01	0.13 ± 0.04
$PEG_5PS_5OVP_{0.2}$	0.17 ± 0.01	0.17 ± 0.02	0.15 ± 0.02	0.14 ± 0.02	0.13 ± 0.02
$PEG_5PS_5OVP_{0.3}$	0.20 ± 0.01	0.19 ± 0.02	0.19 ± 0.01	0.17 ± 0.01	0.15 ± 0.01
$PEG_{14}PS_{12}$	0.16 ± 0.02	0.17 ± 0.02	0.16 ± 0.02	0.16 ± 0.01	0.16 ± 0.02
$PEG_{14}PS_{12}OVP_{0.1}$	0.18 ± 0.02	0.18 ± 0.01	0.18 ± 0.02	0.15 ± 0.01	0.16 ± 0.01
$PEG_{14}PS_{12}OVP_{0.2}$	0.20 ± 0.01	0.19 ± 0.01	0.16 ± 0.01	0.16 ± 0.01	0.14 ± 0.02
$PEG_{14}PS_{12}OVP_{0.3}$	0.24 ± 0.01	0.26 ± 0.02	0.22 ± 0.02	0.16 ± 0.01	0.15 ± 0.01
$PEG_{10}PDL_{10}$	0.14 ± 0.01	0.14 ± 0.01	0.13 ± 0.01	0.14 ± 0.02	0.14 ± 0.02
$PEG_{10}PDL_{10}OVP_{0.1}$	0.16 ± 0.01	0.15 ± 0.02	0.15 ± 0.01	0.14 ± 0.02	0.13 ± 0.01
$PEG_{10}PDL_{10}OVP_{0.2}$	0.17 ± 0.01	0.18 ± 0.02	0.16 ± 0.02	0.15 ± 0.02	0.14 ± 0.01
$PEG_{10}PDL_{10}OVP_{0.3}$	0.19 ± 0.01	0.18 ± 0.03	0.16 ± 0.02	0.14 ± 0.02	0.13 ± 0.01
$PEG_{10}PLA_{10}$	0.17 ± 0.02	0.17 ± 0.01	0.16 ± 0.01	0.16 ± 0.02	0.16 ± 0.01
$PEG_{10}PLA_{10}OVP_{0.1}$	0.18 ± 0.03	0.19 ± 0.04	0.16 ± 0.01	0.16 ± 0.02	0.15 ± 0.01
$PEG_{10}PLA_{10}OVP_{0.2}$	0.21 ± 0.02	0.20 ± 0.01	0.18 ± 0.02	0.17 ± 0.01	0.16 ± 0.02
$PEG_{10}PLA_{10}OVP_{0.3}$	0.22 ± 0.02	0.22 ± 0.03	0.18 ± 0.01	0.17 ± 0.01	0.16 ± 0.02

Table S9: $D_{h,theo}$ and percentage increase in $D_{h,theo}$ relative to blank micelles at 100% OVP EE as a function of OVP loading (0, 0.1, 0.2 and 0.3 mg/mg of copolymer) for the PEG_xHB_y micelles.

Micelles	PEG_5PS_5		$PEG_{14}PS_{12}$		$PEG_{10}PDL_{10}$		$PEG_{10}PLA_{10}$	
	$D_{h,theo}$[a] (nm)	$D_{h,theo}$[b] (%)	$D_{h,theo}$ (nm)	$D_{h,theo}$ (%)	$D_{h,theo}$ (nm)	$D_{h,theo}$ (%)	$D_{h,theo}$ (nm)	$D_{h,theo}$ (%)
Blank	25	0	62	0	35	0	50	0
$OVP_{0.1}$	29	15	75	21	41	16	63	26
$OVP_{0.2}$	33	32	82	33	47	36	70	39
$OVP_{0.3}$	36	46	88	43	52	49	77	53

[a] $D_{h,theo}$ represents theoretical hydrodynamic diameter of the micelles at an OVP EE% of 100%, calculated relative to the D_h of the blank micelles and OVP-loaded micelles. [b] Percentage increase in $D_{h,theo}$ of the OVP-loaded micelles relative to the D_h of the blank micelles.

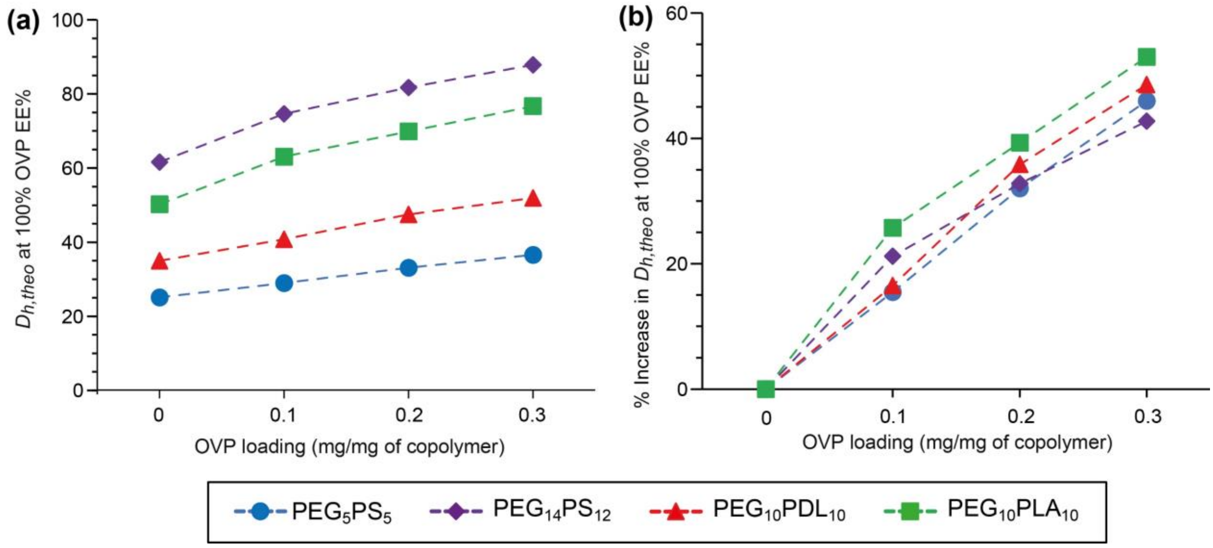

Figure S14: (**a**) $D_{h,theo}$ and (**b**) percentage increase in $D_{h,theo}$ relative to blank micelles at 100% OVP EE as a function of OVP loading (0, 0.1, 0.2 and 0.3 mg/mg of copolymer) for PEG_xHB_y micelles, where assumption of 100% EE serves only as a comparative reference and does not imply complete OVP incorporation.

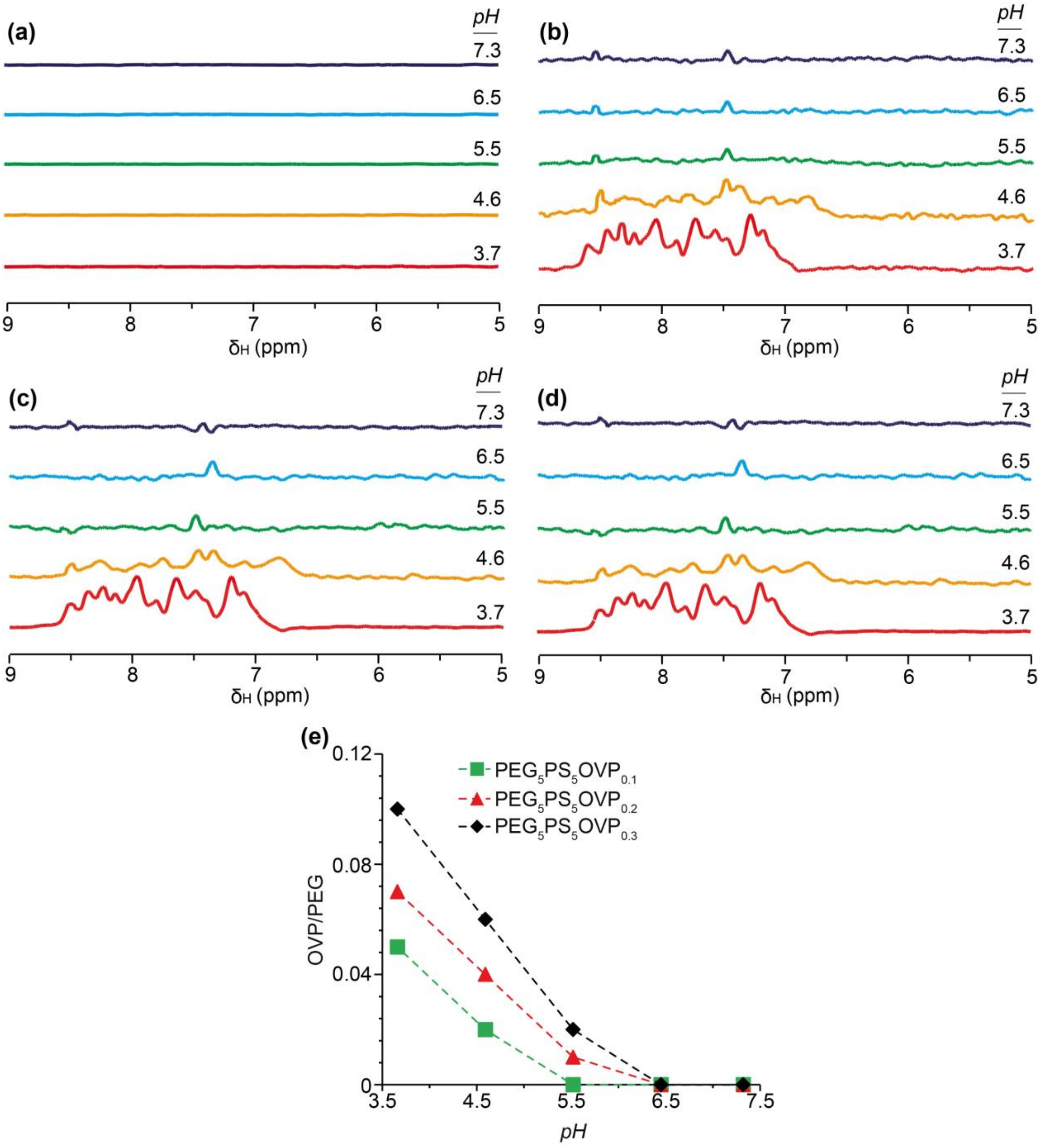

Figure S15: ^{1}H NMR spectra (600 MHz, 25 ± 1 °C, D_2O) of (**a**) blank and (**b-d**) OVP-loaded (0.1, 0.2 and 0.3 mg/mg of copolymer) PEG_5PS_5 micelles as a function of pH. Spectral range (*δH* 5.0–9.0 ppm) limited to show only the region where pyridyl proton resonances from OVP occur. (**e**) pH-dependent change in the intensity of the OVP pyridyl proton resonances with reference to the PEG repeat unit methylene proton resonance (OVP/PEG integral ratio). Only symbols represent the experimental data; the dashed lines are guides to the eyes.

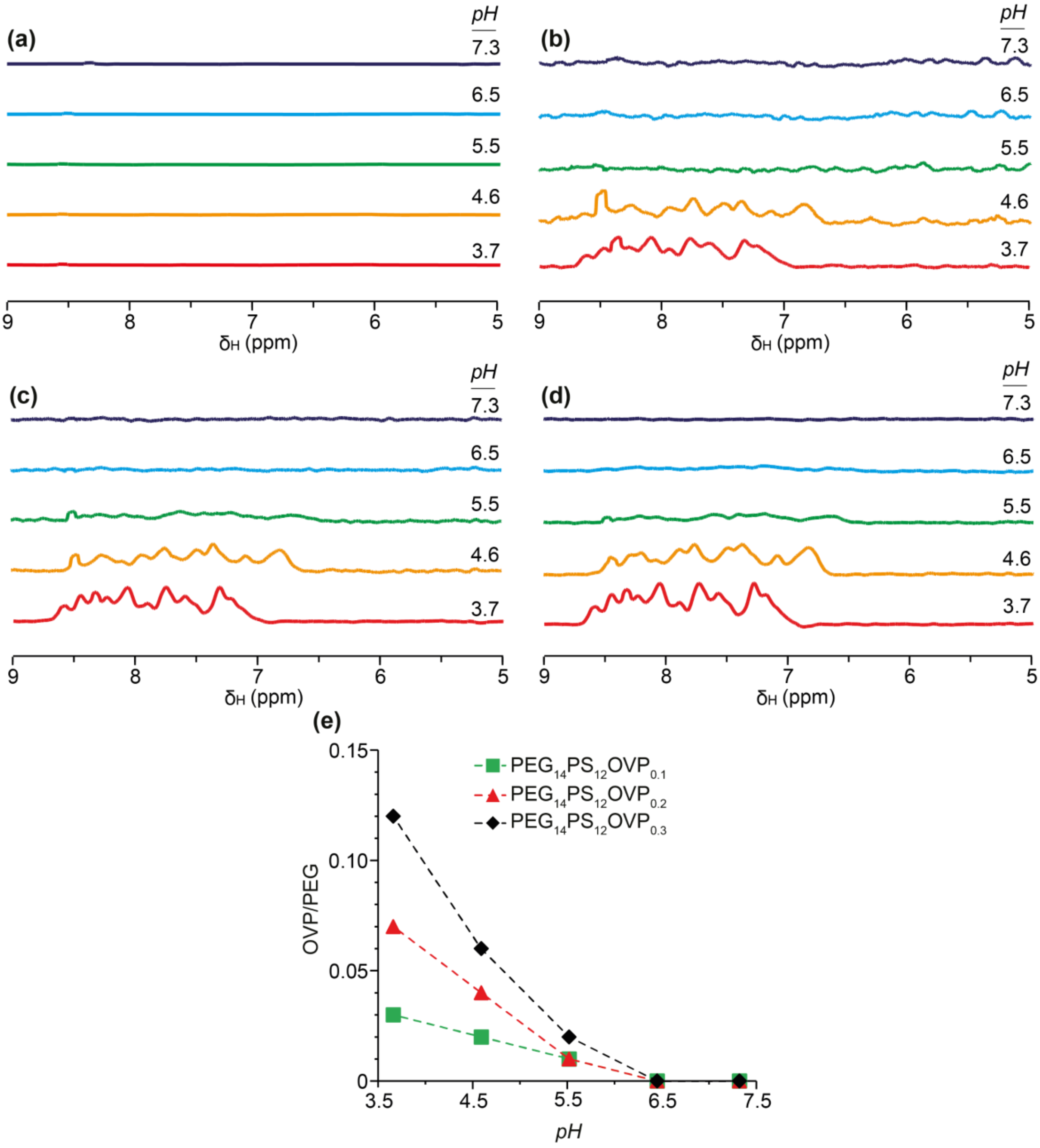

Figure S16: ^{1}H NMR spectra (600 MHz, 25 ± 1 °C, D_2O) of (**a**) blank and (**b-d**) OVP-loaded (0.1, 0.2 and 0.3 mg/mg of copolymer) $PEG_{14}PS_{12}$ micelles as a function of pH. Spectral range (δ_H 5.0–9.0 ppm) limited to show only the region where pyridyl proton resonances from OVP occur. (**e**) pH-dependent change in the intensity of the OVP pyridyl proton resonances with reference to the PEG repeat unit methylene proton resonance (OVP/PEG integral ratio). Only symbols represent the experimental data; the dashed lines are guides to the eyes.

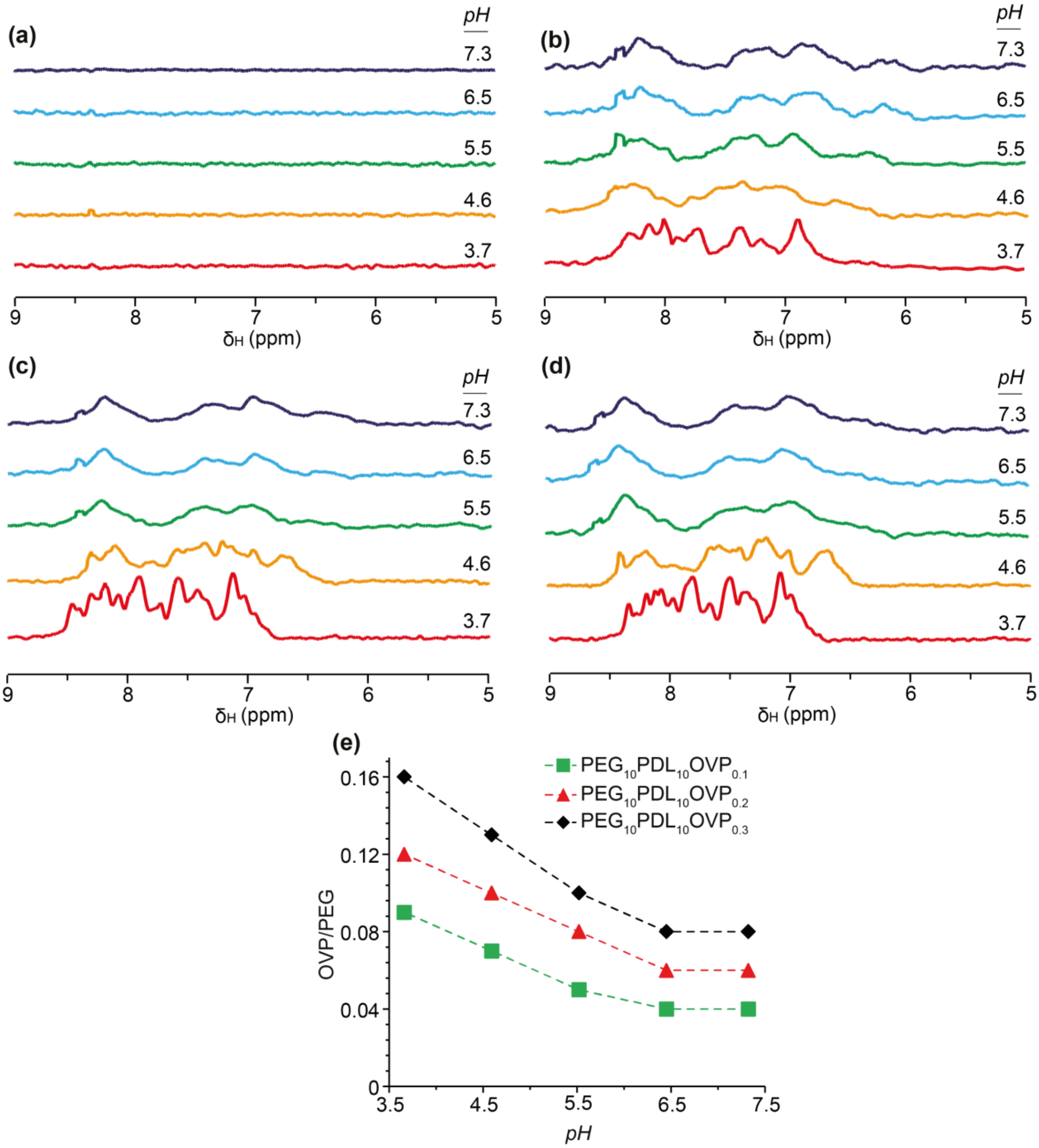

Figure S17: ^{1}H NMR spectra (600 MHz, 25 ± 1 °C, D_2O) of (**a**) blank and (**b-d**) OVP-loaded (0.1, 0.2 and 0.3 mg/mg of copolymer) $PEG_{10}PDL_{10}$ micelles as a function of pH. Spectral range (δ_H 5.0–9.0 ppm) limited to show only the region where pyridyl proton resonances from OVP occur. (**e**) pH-dependent change in the intensity of the OVP pyridyl proton resonances with reference to the PEG repeat unit methylene proton resonance (OVP/PEG integral ratio). Only symbols represent the experimental data; the dashed lines are guides to the eyes.

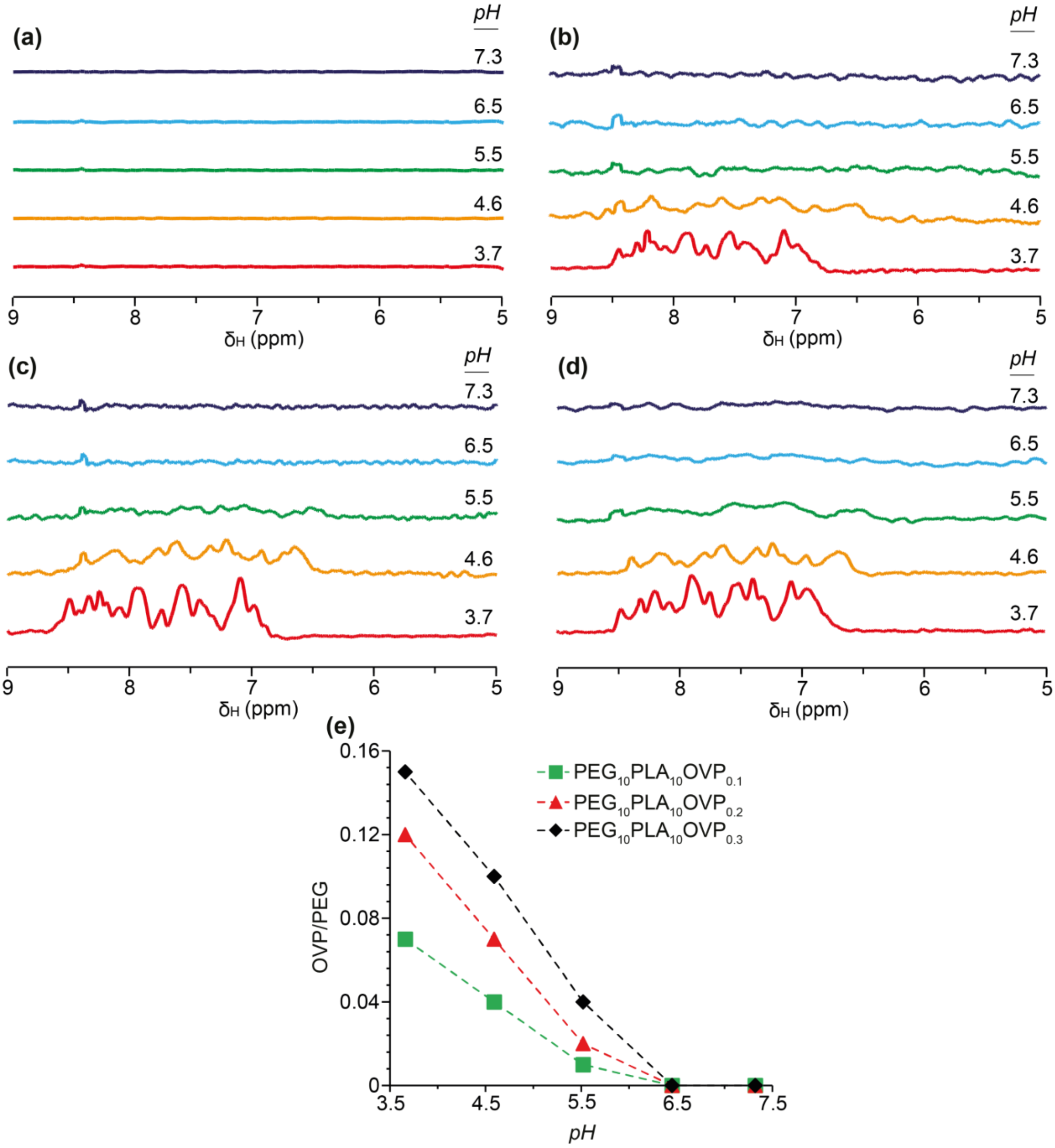

Figure S18: ^{1}H NMR spectra (600 MHz, 25 ± 1 °C, D_2O) of (**a**) blank and (**b-d**) OVP-loaded (0.1, 0.2 and 0.3 mg/mg of copolymer) $PEG_{10}PLA_{10}$ micelles as a function of pH. Spectral range (δ_H 5.0–9.0 ppm) limited to show only the region where pyridyl proton resonances from OVP occur. (**e**) pH-dependent change in the intensity of the OVP pyridyl proton resonances with reference to the PEG repeat unit methylene proton resonance (OVP/PEG integral ratio). Only symbols represent the experimental data; the dashed lines are guides to the eyes.

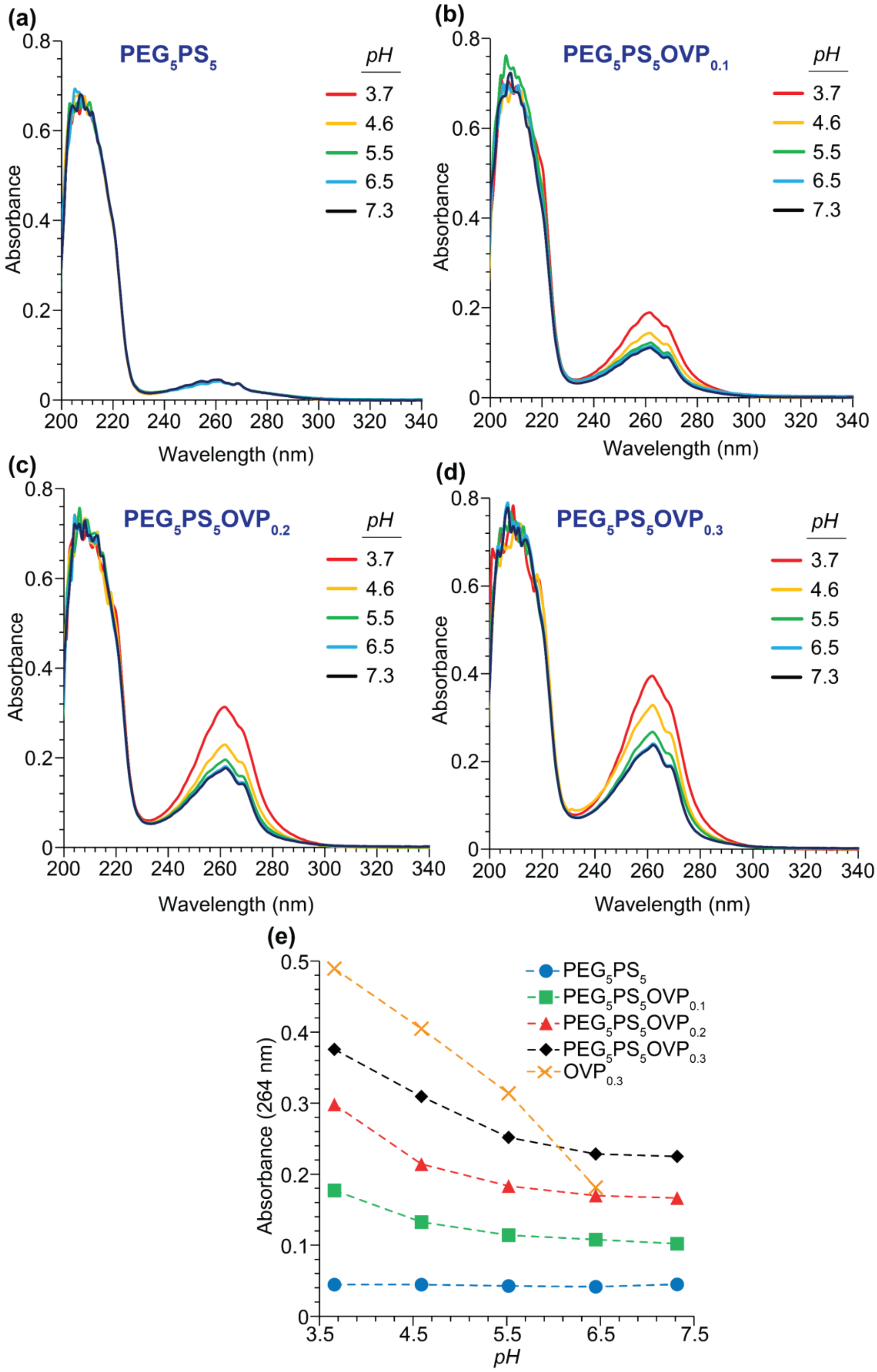

Figure S19: UV-visible spectra of (**a**) blank and (**b–d**) OVP-loaded (0.1, 0.2 and 0.3 mg/mg of copolymer) PEG_5PS_5 micelles and (**e**) UV absorbance (λ_{264}) as a function of pH. Only symbols represent the experimental data; the dashed lines are guides to the eyes.

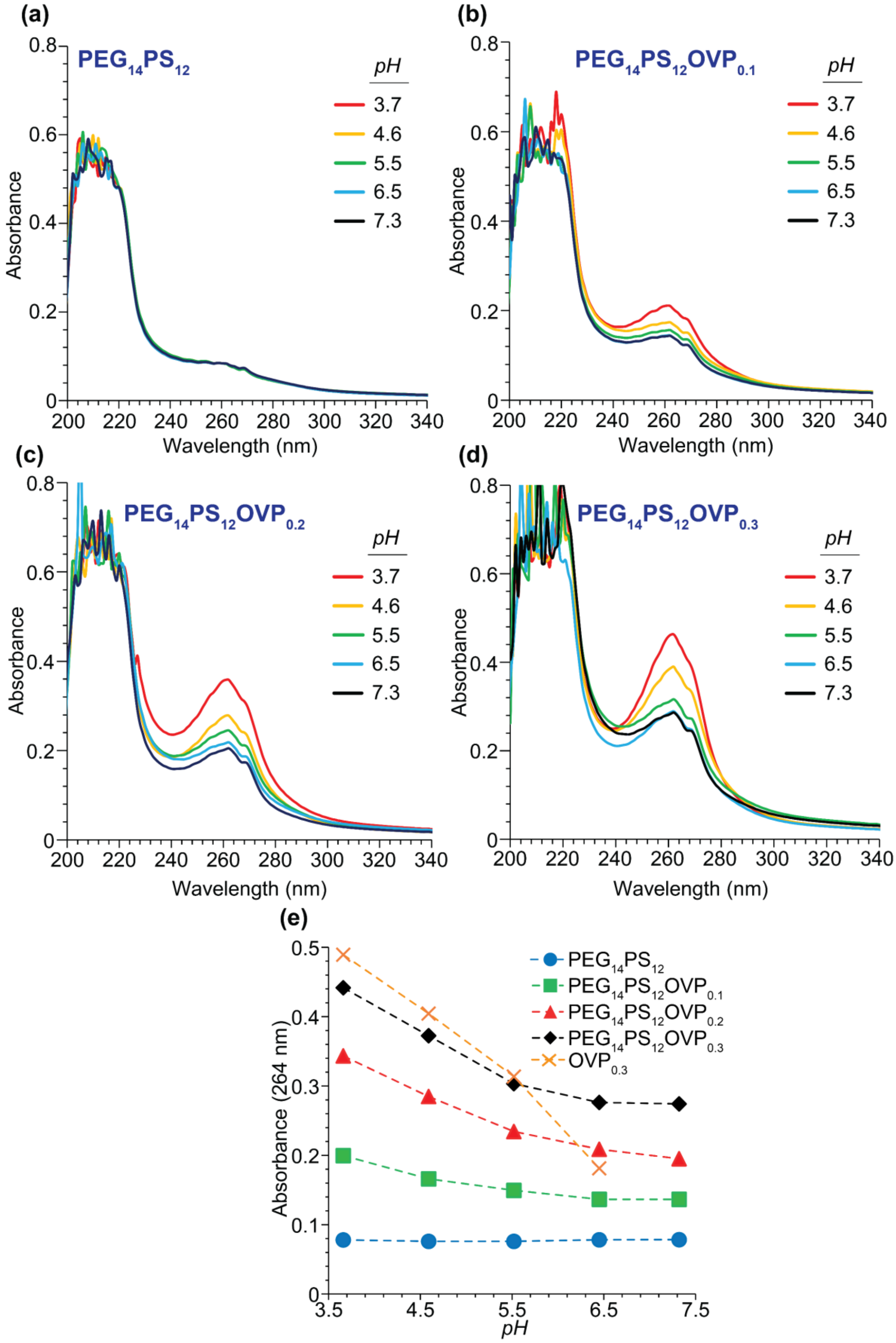

Figure S20: UV-visible spectra of (**a**) blank and (**b**–**d**) OVP-loaded (0.1, 0.2 and 0.3 mg/mg of copolymer) $PEG_{14}PS_{12}$ micelles and (**e**) UV absorbance (λ_{264}) as a function of pH. Only symbols represent the experimental data; the dashed lines are guides to the eyes.

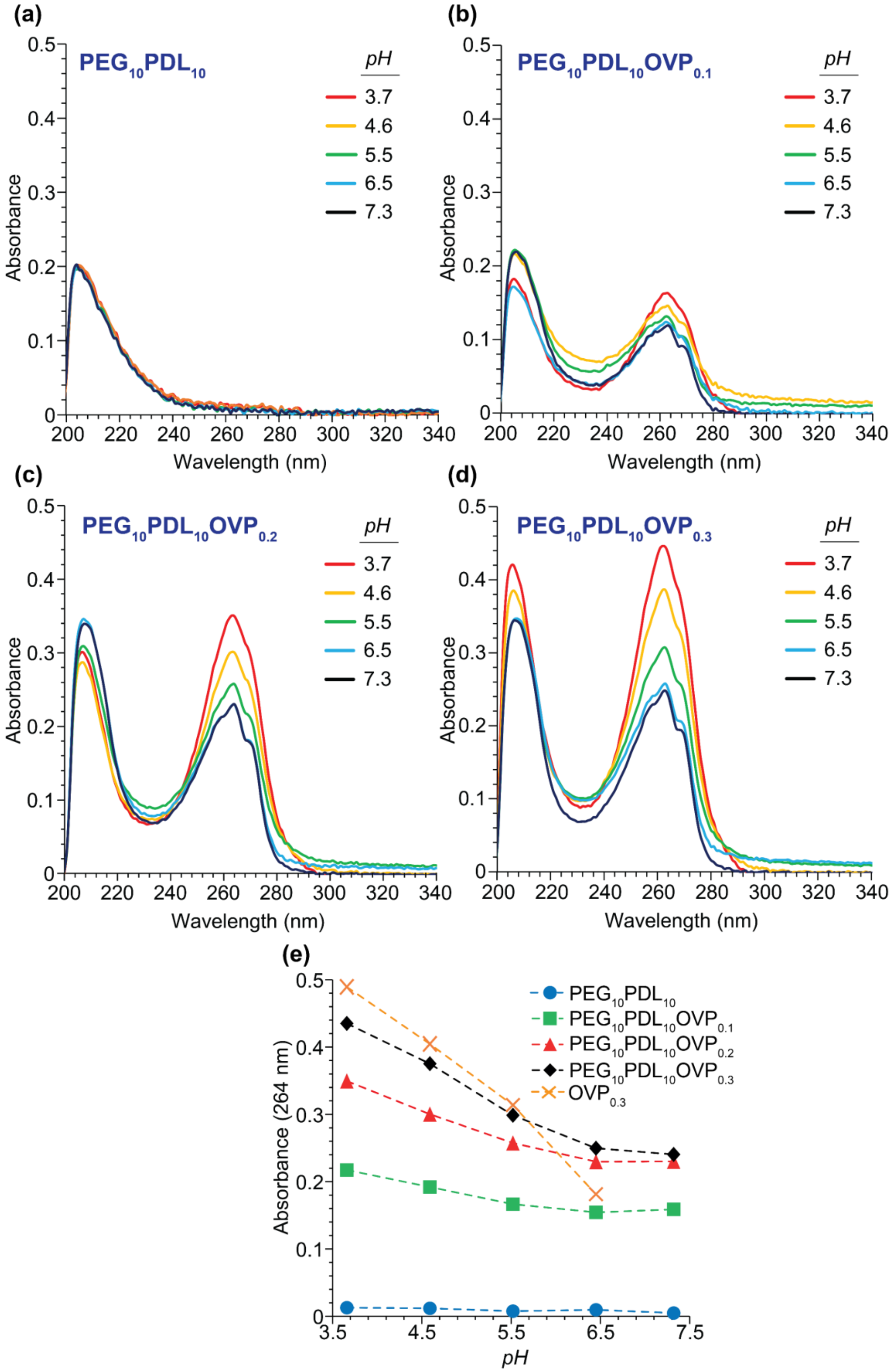

Figure S21: UV-visible spectra of (**a**) blank and (**b–d**) OVP-loaded (0.1, 0.2 and 0.3 mg/mg of copolymer) PEG$_{10}$PDL$_{10}$ micelles and (**e**) UV absorbance (λ_{264}) as a function of pH. Only symbols represent the experimental data; the dashed lines are guides to the eyes.

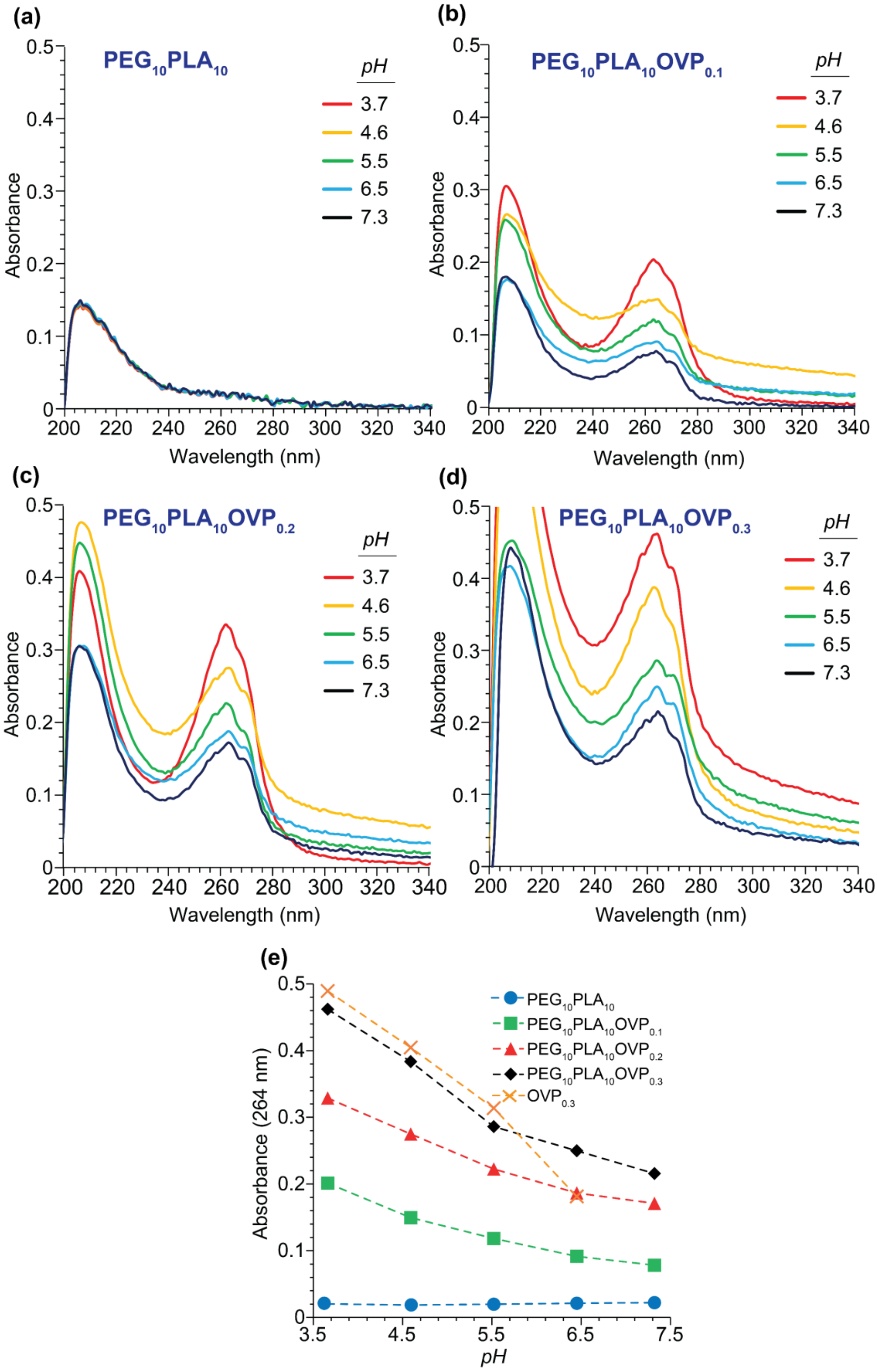

Figure S22: UV-visible spectra of (**a**) blank and (**b**–**d**) OVP-loaded (0.1, 0.2 and 0.3 mg/mg of copolymer) $PEG_{10}PLA_{10}$ and (**e**) UV absorbance (λ_{264}) as a function of pH. Only symbols represent the experimental data; the dashed lines are guides to the eyes.

Table S10: Concentration and volume of stock solutions combined to prepare OVP-loaded (0.1, 0.2 and 0.3 mg/mg of copolymer) PEG_xHB_y micelles in 10 mM PBS (pH 7.4) for OVP release studies.

[a] Micelle composition	Solvent used for copolymer, OVP and drug stock solutions	Conc. of copolymer stock solution (mg/L)	Volume of copolymer stock solution (μL)	OVP mass fraction (mg/mg of copolymer)	Conc. of OVP stock solution (mg/L)	Volume of OVP stock solution (μL)	Volume of PBS added (10 mM, pH 7.4) (μL)
$PEG_xHB_yOVP_{0.1}$	Acetone	1000	6000	0.1	1000	600	6000
$PEG_xHB_yOVP_{0.2}$	Acetone	1000	6000	0.2	1000	1200	6000
$PEG_xHB_yOVP_{0.3}$	Acetone	1000	6000	0.3	1000	1800	6000

[a] Micellar solutions were prepared in PBS via the solvent evaporation approach. Stock solutions of all polymers. HB stands for hydrophobic block and represents either PS, PDL and PLA.

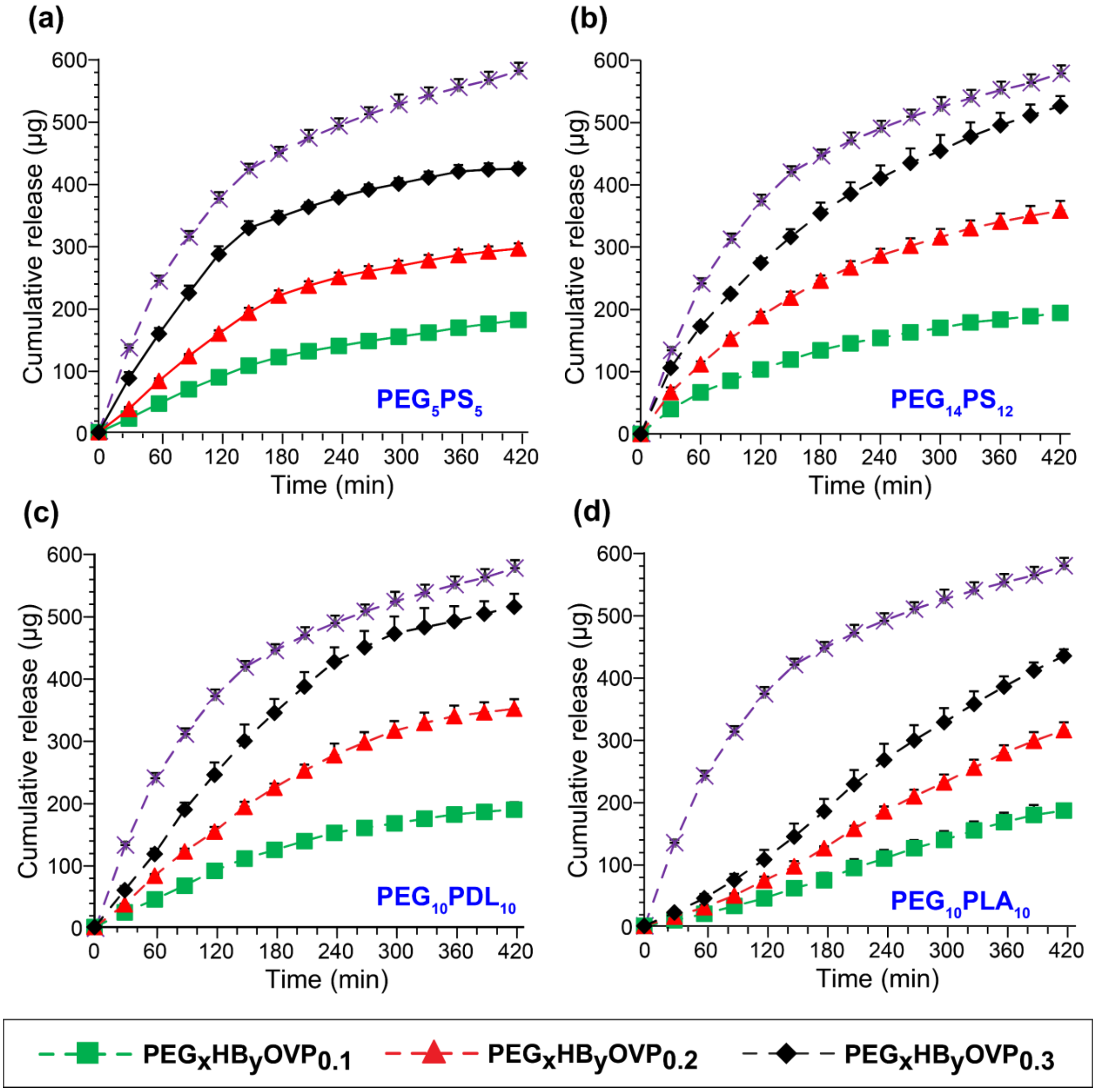

Figure S23: *In vitro* OVP release from OVP-loaded (0.1, 0.2 and 0.3 mg/mg of copolymer) **(a)** PEG_5PS_5, **(b)** $PEG_{14}PS_{12}$, **(c)** $PEG_{10}PDL_{10}$, and **(d)** $PEG_{10}PLA_{10}$ micelles in acetate buffer (10 mM, pH 4.5) at ambient temperature. Purple line represents the release of free OVP (equivalent to 0.3 mg/mg of copolymer loading) dissolved in acetate buffer (10 mM, pH 4.5). Data are shown as the average cumulative release (μg) + SD (n = 3). Only symbols represent the experimental data; the dashed lines are guides to the eyes.

Table S11: Model fitting for the prediction of release behaviour for the release of OVP from OVP-loaded PEG_5PS_5, $PEG_{14}PS_{12}$, $PEG_{10}PDL_{10}$, and $PEG_{10}PLA_{10}$ micelles and the diffusion of free OVP across the dialysis membrane (MWCO = 100 kDa) in acetate buffer (10 mM, pH 4.5).

Sample	**Release models, exponents and goodness-of-fit[a]**									
	First-order			Higuchi			Korsmeyer-Peppas			
	R^2	MSE	MSC	R^2	MSE	MSC	n	R^2	MSE	MSC
$OVP_{0.1}$	1.00	2.38	**5.15**	0.94	50.83	2.09	**0.39**	0.97	28.59	2.60
$OVP_{0.2}$	1.00	2.14	**5.32**	0.97	21.66	3.01	**0.44**	0.98	18.04	3.13
$OVP_{0.3}$	0.99	6.83	**4.05**	0.97	24.54	2.77	**0.43**	0.98	17.65	3.04
$PEG_5PS_5OVP_{0.1}$	1.00	2.37	**5.37**	0.97	26.01	2.97	**0.62**	0.98	13.37	3.58
$PEG_5PS_5OVP_{0.2}$	0.98	11.40	**3.44**	0.96	22.38	2.77	**0.57**	0.97	20.21	2.81
$PEG_5PS_5OVP_{0.3}$	0.91	42.09	1.84	0.95	23.31	**2.43**	**0.44**	0.96	21.93	**2.41**
$PEG_{14}PS_{12}OVP_{0.1}$	1.00	3.63	**4.92**	0.99	7.26	4.22	**0.53**	0.99	6.32	4.30
$PEG_{14}PS_{12}OVP_{0.2}$	1.00	0.45	**6.92**	0.99	10.66	3.75	**0.56**	0.99	7.81	4.00
$PEG_{14}PS_{12}OVP_{0.3}$	1.00	2.48	**5.10**	0.99	5.95	4.23	**0.55**	0.99	3.75	4.63
$PEG_{10}PDL_{10}OVP_{0.1}$	0.99	12.35	**3.91**	0.96	41.11	2.71	**0.64**	0.98	19.35	3.40
$PEG_{10}PDL_{10}OVP_{0.2}$	0.99	8.76	**4.17**	0.95	45.45	2.52	**0.68**	0.98	16.53	3.47
$PEG_{10}PDL_{10}OVP_{0.3}$	0.99	6.04	**4.47**	0.95	40.89	2.56	**0.64**	0.97	23.55	3.05
$PEG_{10}PLA_{10}OVP_{0.1}$	0.91	94.14	2.08	0.82	185.78	1.40	**1.10**	1.00	4.48	**5.07**
$PEG_{10}PLA_{10}OVP_{0.2}$	0.93	49.29	2.39	0.81	136.96	1.37	**1.12**	1.00	3.35	**5.02**
$PEG_{10}PLA_{10}OVP_{0.3}$	0.95	28.77	2.75	0.83	104.16	1.46	**1.06**	0.99	3.92	**4.68**

[a] All modelling was conducted using the DDSolver plugin for excel [5]. For each model, the coefficient of determination (R^2), mean square error (MSE) and model selection criterion (MSC) were calculated to determine the goodness-of-fit, and therefore, the most appropriate model for describing the release behaviour. An R^2 value closer to 1 indicates a closer correlation between the response values and the predicted response values. A MSE value closer to 0 indicates a closer correlation between the response values and the predicted response values. The MSC is a modified reciprocal form of the Akaike information criterion (AIC), which is normalized to be independent of the scaling of the data points. A larger MSC indicates a closer correlation between the response values and the predicted response values. The MSC was used to determine the best model fit to the release curves for each sample, as shown by the green color coding; alternative models that gave a MSC value within 0.1 unit of the best fit are shown with light green color coding. Light blue, dark blue and purple color coding for the release exponent corresponds to Fickian diffusion ($n < 0.45$), anomalous non-Fickian diffusion ($0.45 < n < 0.85$) and case I/super case II transport ($n > 0.85$) from a polymer matrix between cylindrical and spherical morphologies [6,7].

Table S12: Concentration and volume of stock solutions combined to prepare copolymer solutions in 10 mM PBS (pH 7.4) for the drug EE% analysis for blank and OVP-loaded (0.1, 0.2 and 0.3 mg/mg of copolymer) PEG_5PS_5, $PEG_{14}PS_{12}$, $PEG_{10}PDL_{10}$ and $PEG_{10}PLA_{10}$ copolymer micelles. Samples were prepared in triplicate.

Micelle composition[a]	Solvent used for copolymer, OVP and drug stock solutions	Conc. of copolymer stock solution (mg/L)	Volume of copolymer stock solution (μL)	OVP mass fraction (mg/mg of copolymer)	Conc. of OVP stock solution (mg/L)	Volume of OVP stock solution (μL)	Drug mass fraction (mg/mg of copolymer)	Conc. of drug stock solution (mg/L)	Volume of drug stock solution added (μL)	Volume of PBS added (10 mM, pH 7.4) (μL)
$PEG_xHB_yD_{0.1}$	Acetone	1000	1000	-	-	-	0.1	1000	100	1000
$PEG_xHB_yD_{0.2}$	Acetone	1000	1000	-	-	-	0.1	1000	200	1000
$PEG_xHB_ySN38_{0.1}$	THF	1000	1000	-	-	-	0.1	1000	100	1000
$PEG_xHB_ySN38_{0.2}$	THF	1000	1000	-	-	-	0.1	1000	200	1000
$PEG_xHB_yOVP_{0.1}D_{0.1}$	Acetone	1000	1000	0.1	1000	100	0.1	1000	100	1000
$PEG_xHB_yOVP_{0.1}D_{0.2}$	Acetone	1000	1000	0.1	1000	100	0.1	1000	200	1000
$PEG_xHB_yOVP_{0.2}D_{0.1}$	Acetone	1000	1000	0.2	1000	200	0.1	1000	100	1000
$PEG_xHB_yOVP_{0.2}D_{0.2}$	Acetone	1000	1000	0.2	1000	200	0.1	1000	200	1000
$PEG_xHB_yOVP_{0.2}D_{0.1}$	Acetone	1000	1000	0.3	1000	300	0.1	1000	100	1000
$PEG_xHB_yOVP_{0.3}D_{0.2}$	Acetone	1000	1000	0.3	1000	300	0.1	1000	200	1000
$PEG_xHB_yOVP_{0.1}SN38_{0.1}$	THF	1000	1000	0.1	1000	100	0.1	1000	100	1000
$PEG_xHB_yOVP_{0.1}SN38_{0.2}$	THF	1000	1000	0.1	1000	100	0.1	1000	200	1000
$PEG_xHB_yOVP_{0.2}SN38_{0.1}$	THF	1000	1000	0.2	1000	200	0.1	1000	100	1000
$PEG_xHB_yOVP_{0.2}SN38_{0.2}$	THF	1000	1000	0.2	1000	200	0.1	1000	200	1000
$PEG_xHB_yOVP_{0.2}SN38_{0.1}$	THF	1000	1000	0.3	1000	300	0.1	1000	100	1000
$PEG_xHB_yOVP_{0.3}SN38_{0.2}$	THF	1000	1000	0.3	1000	300	0.1	1000	200	1000

[a]Micellar solutions were prepared in PBS via the solvent evaporation approach. Stock solutions of all polymers and drugs (D; DOX, GP and PX) were prepared in acetone, with the exception of SN38, for which stocks solutions of the polymers and SN38 was prepared in THF. HB stands for hydrophobic block and represents either PS, PDL or PLA.

Table S13: EE% of drugs (DOX, GP, PX and SN38) and OVP (refer to values provided in brackets) for OVP- and drug-loaded PEG_XHB_Y micelles. All values are reported as the mean ± S.D (n = 3). Colour coding is for quick referencing of the EE% values: green ≥ 90.0%; light green = 80.0 – 89.9%; orange = 70.0 – 79.9%; yellow = 60.0 – 69.9%; red < 60.0%.

Polymers	DOX (OVP) loading (%)		GP (OVP) loading (%)		PX (OVP) loading %		SN38 (OVP) loading %	
	$DOX_{0.1}$	$DOX_{0.2}$	$GP_{0.1}$	$GP_{0.2}$	$PX_{0.1}$	$PX_{0.2}$	$SN38_{0.1}$	$SN38_{0.2}$
**PEG_5PS_5**	94.3 ± 0.4	91.6 ± 0.4	96.4 ± 1.7	93.8 ± 0.5	97.4 ± 0.7	94.1 ± 1.2	16.9 ± 1.9	11.7 ± 2.5
**$PEG_5PS_5OVP_{0.1}$**	92.2 ± 1.6 (87.9 ± 2.8)	82.8 ± 3.2 (83.3 ± 0.5)	94.0 ± 0.7 (87.9 ± 0.6)	91.7 ± 1.4 (81.6 ± 2.6)	93.6 ± 0.7 (92.2 ± 1.6)	91.4 ± 1.4 (84.3 ± 2.2)	–[a]	–[a]
**$PEG_5PS_5OVP_{0.2}$**	80.2 ± 2.4 (81.1 ± 0.9)	71.4 ± 1.5 (75.3 ± 2.1)	82.6 ± 4.6 (83.1 ± 1.5)	75.4 ± 1.7 (77.4 ± 1.4)	80.3 ± 0.9 (85.1 ± 2.2)	72.3 ± 3.3 (76.3 ± 1.9)	–[a]	–[a]
**$PEG_5PS_5OVP_{0.3}$**	70.2 ± 1.8 (74.9 ± 2.9)	65.9 ± 2.2 (65.7 ± 3.4)	75.5 ± 2.1 (70.3 ± 1.9)	67.4 ± 1.7 (59.8 ± 2.4)	72.1 ± 2.1 (71.2 ± 3.1)	62.9 ± 4.4 (54.6 ± 2.5)	–[a]	–[a]
**$PEG_{14}PS_{12}$**	98.1 ± 0.1	97.1 ± 0.5	98.5 ± 0.3	97.7± 0.8	96.9 ± 0.1	95.5 ± 0.2	93.2 ± 0.5	90.5 ± 0.8
**$PEG_{14}PS_{12}OVP_{0.1}$**	96.4 ± 1.6 (93.2 ± 1.8)	94.3 ± 0.2 (87.3 ± 1.1)	97.1 ± 0.5 (94.8 ± 0.9)	96.4 ± 0.4 (89.1 ± 1.9)	96.8 ± 0.2 (94.3 ± 0.8)	91.2 ± 2.4 (91.5 ± 1.3)	92.2 ± 0.6 (93.7 ± 1.1)	89.1 ± 1.3 (92.2 ± 1.5)
**$PEG_{14}PS_{12}OVP_{0.2}$**	91.4 ± 0.6 (93.5 ± 1.6)	89.7 ± 0.9 (86.1 ± 1.8)	92.4 ± 1.1 (93.8 ± 0.4)	90.1 ± 0.6 (90.2 ± 0.9)	91.9 ± 0.9 (87.7 ± 1.5)	82.7 ± 0.9 (86.5 ± 1.7)	82.1 ± 1.2 (85.6 ± 1)	78.1 ± 1.5 (82.6 ± 1.2)
**$PEG_{14}PS_{12}OVP_{0.3}$**	89.3 ± 2.1 (83.1 ± 1.4)	83.3 ± 1.8 (80.6 ± 1.9)	89.9 ± 1.1 (91.2 ± 0.4)	85.5 ± 0.8 (83.4 ± 1.2)	82.6 ± 1.2 (84.1 ± 1.6)	80.4 ± 1.3 (80.3 ± 1.8)	75.5 ± 1.4 (77.1 ± 2.4)	70.9 ± 4.4 (74.3 ± 2.8)
**$PEG_{10}PDL_{10}$**	96.9 ± 0.5	93.3 ± 0.2	98.4 ± 1.3	95.9 ± 0.6	95.4 ± 0.8	93.3± 1.2	93.9 ± 0.9	90.8 ± 1.2
**$PEG_{10}PDL_{10}OVP_{0.1}$**	95.9 ± 0.6 (88.5 ± 0.5)	92.1 ± 0.3 (81.1 ± 1.2)	97.6 ± 1.3 (90.1 ± 1.1)	93.7 ± 0.9 (86.7 ± 0.8)	93.5 ± 0.8 (89.2 ± 4.4)	87.9 ± 0.8 (82.8 ± 4.5)	88.2 ± 1.2 (87.7 ± 3.2)	84.2 ± 0.5 (80.9 ± 2.1)
**$PEG_{10}PDL_{10}OVP_{0.2}$**	93.1 ± 0.8 (82.6 ± 1.3)	83.6 ± 1.9 (80.2 ± 1.1)	94.8 ± 0.6 (85.4 ± 0.6)	86.4 ± 0.8 (80.9 ± 1.5)	88.7 ± 3.3 (88.8 ± 1.3)	83.1 ± 1.1 (82.7 ± 0.5)	82.1 ± 1.2 (81.2 ± 1)	76.2 ± 1.1 (75.7 ± 2.7)
**$PEG_{10}PDL_{10}OVP_{0.3}$**	82.6 ± 0.8 (79.9 ± 1.5)	76.9 ± 1.2 (73.6 ± 2.7)	84.2 ± 0.7 (82.6 ± 1.4)	78.3 ± 1.1 (74.9 ± 2.3)	83.7 ± 0.7 (80.6 ± 1.3)	76.5 ± 2.8 (73.6 ± 1.2)	71.5 ± 1.5 (70.3 ± 2.9)	64.7 ± 2.1 (64.5 ± 1.6)
**$PEG_{10}PLA_{10}$**	95.8 ± 0.8	91.8 ± 0.6	96.3 ± 0.6	94.8 ± 1.1	96.1 ± 0.7	91.9 ± 1.6	90.1 ± 1.8	89.9 ± 0.5
**$PEG_{10}PLA_{10}OVP_{0.1}$**	93.2 ± 0.4 (89.6 ± 0.8)	88.2 ± 1.1 (82.5 ± 0.9)	95.8 ± 3.4 (94.6 ± 1.5)	93.9 ± 1.2 (86.9 ± 3.7)	95.2 ± 1.6 (91.1 ± 2.4)	91.3 ± 1.1 (85.7 ± 2.7)	88.8 ± 1.9 (87.7 ± 2.2)	87.7 ± 2.2 (82.9 ± 1.7)
**$PEG_{10}PLA_{10}OVP_{0.2}$**	89.5 ± 1.7 (82.3 ± 1.6)	81.9 ± 0.6 (79.3 ± 0.7)	90.3 ± 0.9 (87.1 ± 3.6)	82.7 ± 0.7 (83.1 ± 0.7)	87.6 ± 1.4 (87.7 ± 1.5)	82.9 ± 1.7 (80.8 ± 2.2)	82.8 ± 1.1 (80.6 ± 0.6)	76.6 ± 0.9 (71.6 ± 1.7)
**$PEG_{10}PLA_{10}OVP_{0.3}$**	80.7 ± 1.6 (78.9 ± 2.1)	75.3± 2.3 (72.7 ± 2.6)	83.1 ± 3.5 (81.1 ± 1.3)	75.7 ± 2.5 (72.2 ± 1.6)	80.2 ± 2.1 (79.3 ± 0.5)	72.2 ± 1.6 (69.1 ± 1.1)	70.3 ± 0.9 (70.9 ± 3.1)	62.5 ± 1.9 (61.9 ± 3.4)

[a] Due to the very low SN38 EE% for the PEG_5PS_5 micelles in the absence of OVP, no attempts were made to co-encapsulate SN38 and OVP.

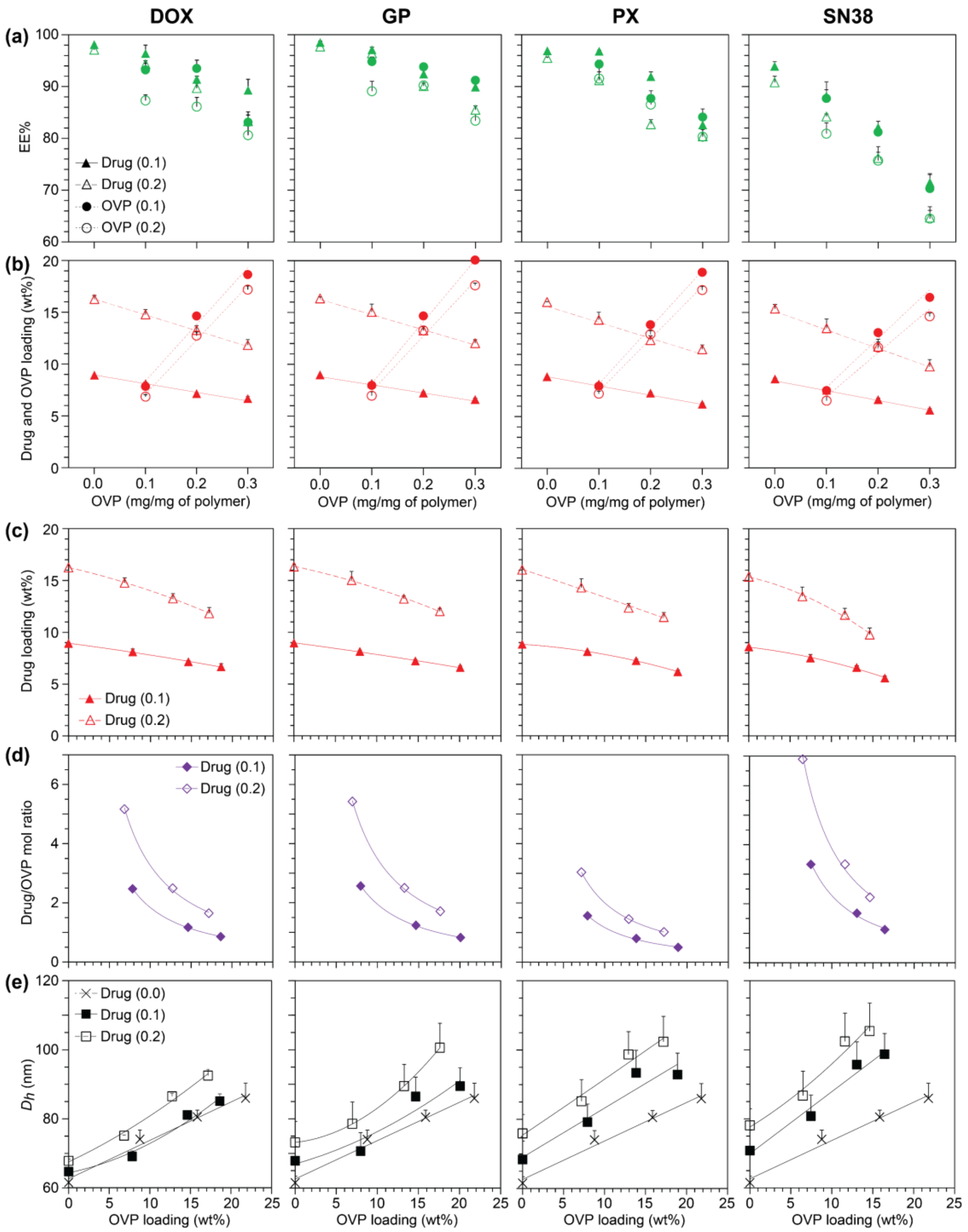

Figure S24: Characterisation of drug- and OVP-loaded $PEG_{14}PS_{12}$ micelles. Columns correspond to the respective drugs indicated at the top. Drug and OVP **(a)** EE% and **(b)** loading (wt%) relative to the initial OVP loading (mg/mg of copolymer) used to prepare micelles at drug loadings of 0.1 and 0.2 mg/mg of copolymer (as indicated by numbers in brackets in legends). Symbols represent the experimental data; the (dashed /solid) lines are guides to the eyes. **(c)** Drug loading (wt%), **(d)** drug/OVP mole ratio and **(e)** micelle D_h (D_h of blank micelles also shown for comparison) relative to the experimentally determined OVP loading (wt%) at drug loadings of 0.1 and 0.2 mg/mg of copolymer (as indicated by numbers in brackets in legends). All data are shown as the mean + SD (n = 3). Symbols represent the experimental data; dashed/dotted/solid lines represent power law fits to the data.

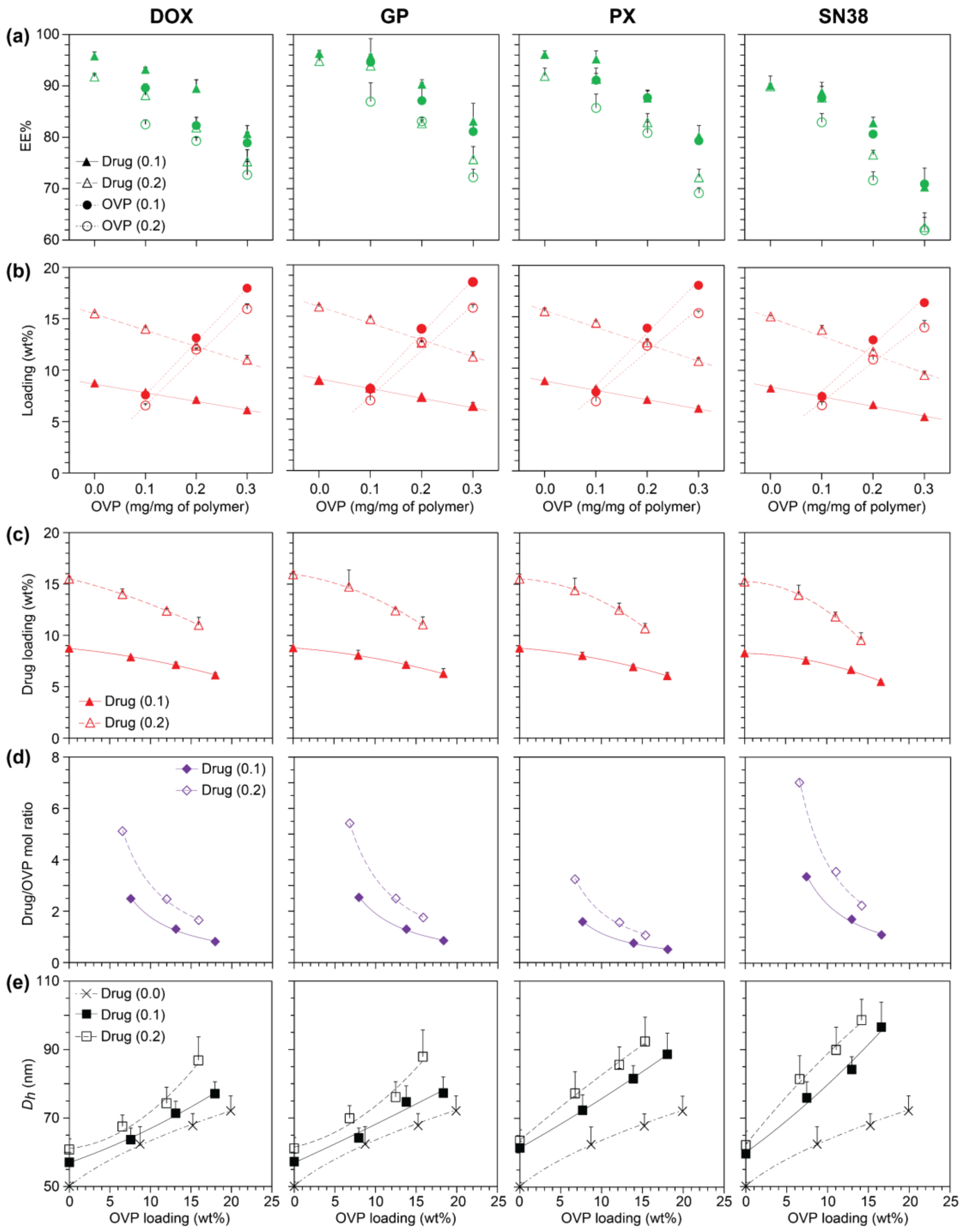

Figure S25: Characterisation of drug- and OVP-loaded $PEG_{10}PLA_{10}$ micelles. Columns correspond to the respective drugs indicated at the top. Drug and OVP **(a)** EE% and **(b)** loading (wt%) relative to the initial OVP loading (mg/mg of copolymer) used to prepare micelles at drug loadings of 0.1 and 0.2 mg/mg of copolymer (as indicated by numbers in brackets in legends). Symbols represent the experimental data; the (dashed /solid) lines are guides to the eyes. **(c)** Drug loading (wt%), **(d)** drug/OVP mole ratio and **(e)** micelle D_h (D_h of blank micelles also shown for comparison) relative to the experimentally determined OVP loading (wt%) at drug loadings of 0.1 and 0.2 mg/mg of copolymer (as indicated by numbers in brackets in legends). All data are shown as the mean + SD (n = 3). Symbols represent the experimental data; dashed/dotted/solid lines represent power law fits to the data.

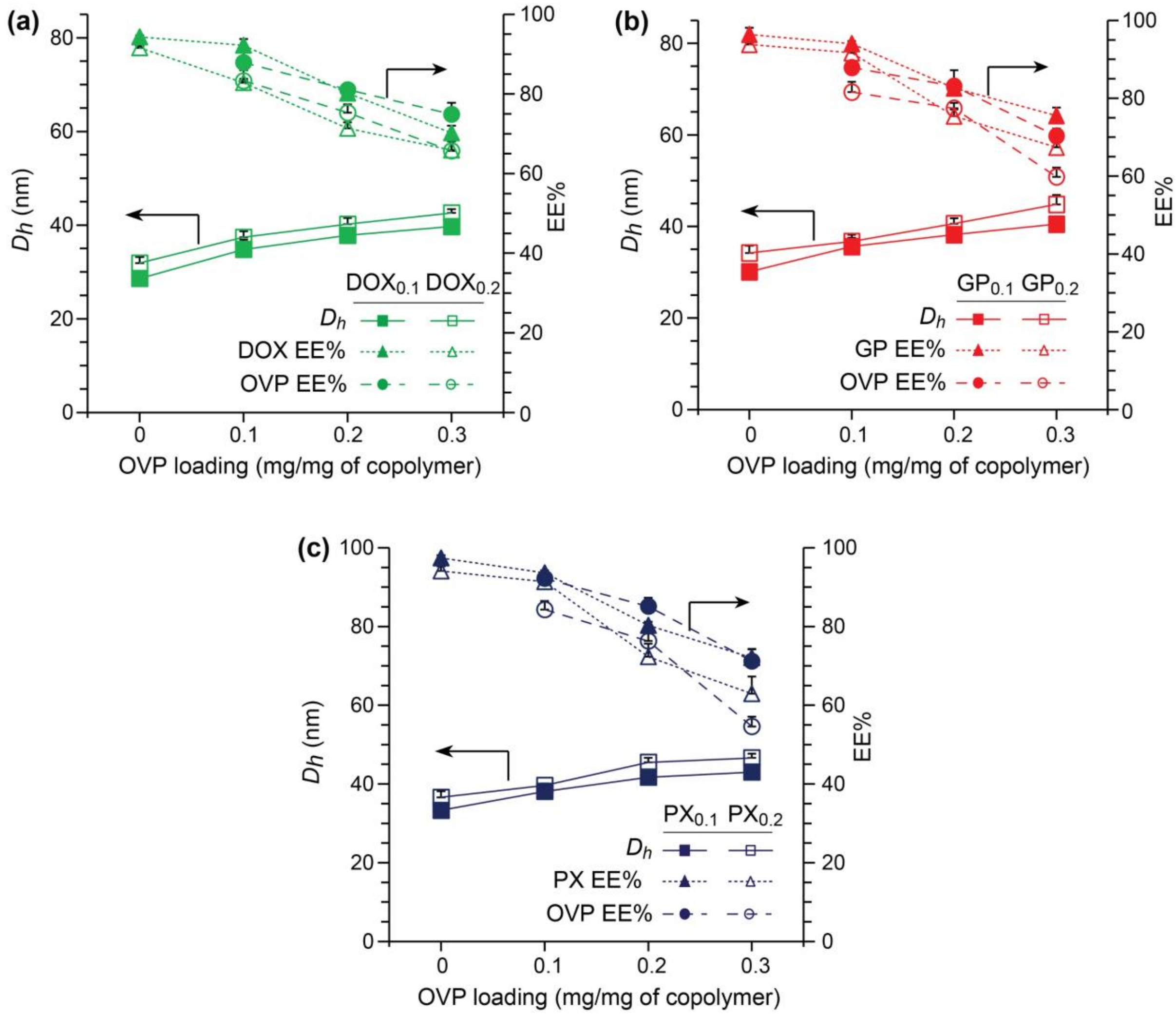

Figure S26: D_h (full symbols; primary y-axis) determined from DLS number PSDs and EE% of (**a**) DOX-, (**b**) GP- and (**c**) PX-loaded (dotted lines; secondary y-axis) versus OVP loading for PEG_5PS_5 micelles. The EE% of OVP is also provided on the secondary y-axis (empty symbols; secondary y-axis). Data are shown as the mean + SD (n = 3). Only symbols represent the experimental data; the (dashed/dotted/solid) lines are guides to the eyes.

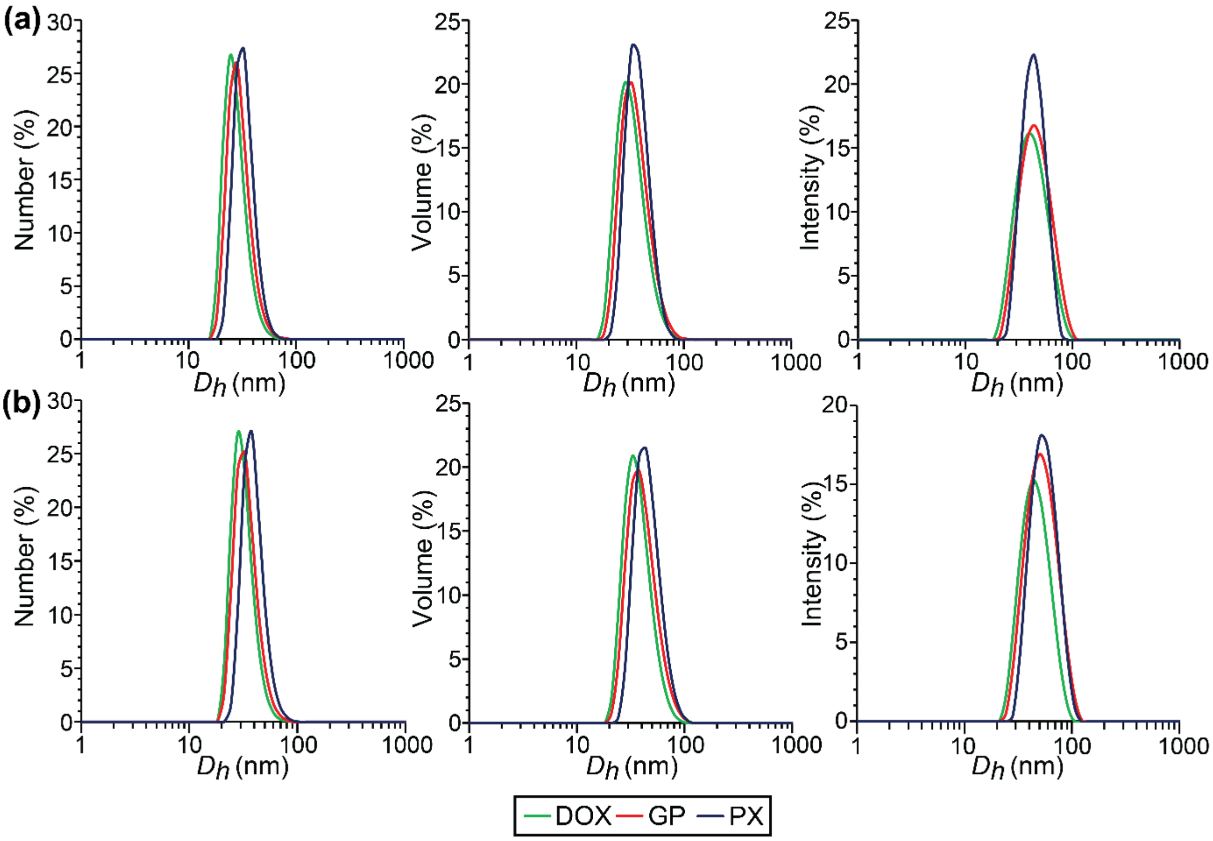

Figure S27: Number, volume and intensity PSDs for **(a)** $PEG_5PS_5D_{0.1}$ and **(b)** $PEG_5PS_5D_{0.2}$ micelles, as measured by DLS.

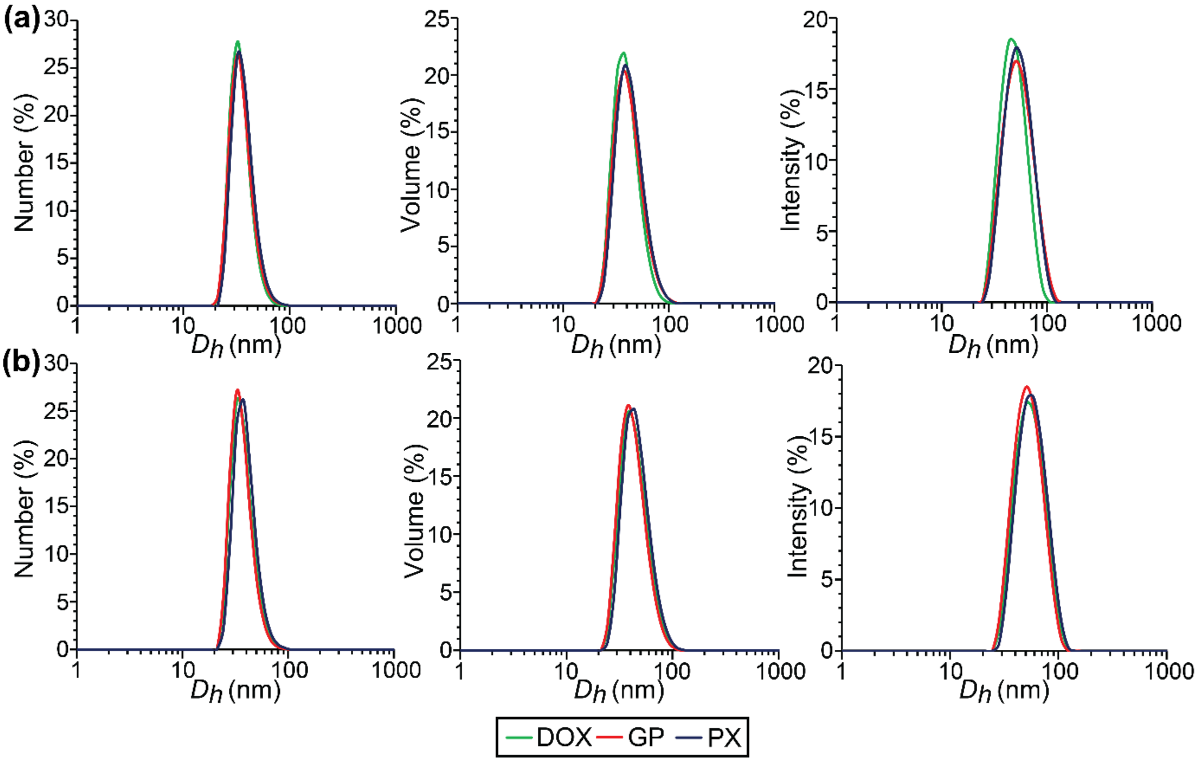

Figure S28: Number, volume and intensity PSDs for (a) $PEG_5PS_5OVP_{0.1}D_{0.1}$ and (b) $PEG_5PS_5OVP_{0.1}D_{0.2}$ micelles, as measured by DLS.

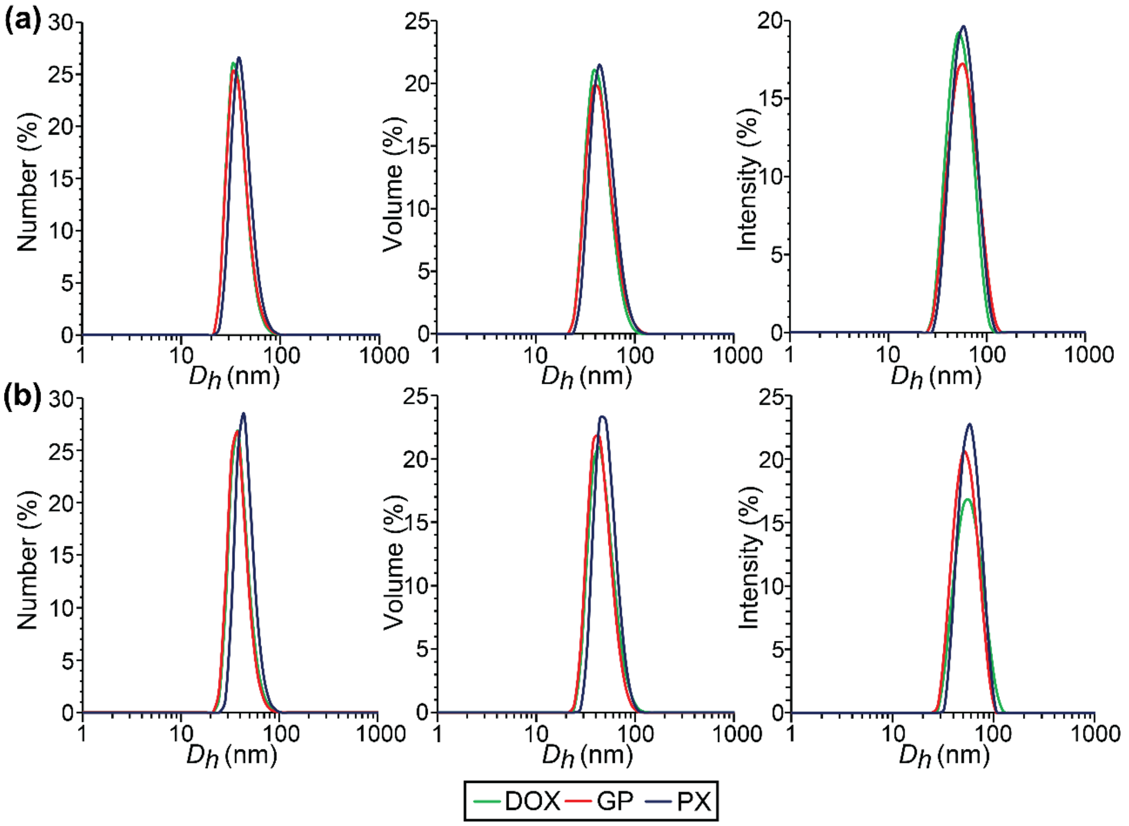

Figure S29: Number, volume and intensity PSDs for (a) PEG$_5$PS$_5$OVP$_{0.2}$D$_{0.1}$ and (b) PEG$_5$PS$_5$OVP$_{0.2}$D$_{0.2}$ micelles, as measured by DLS.

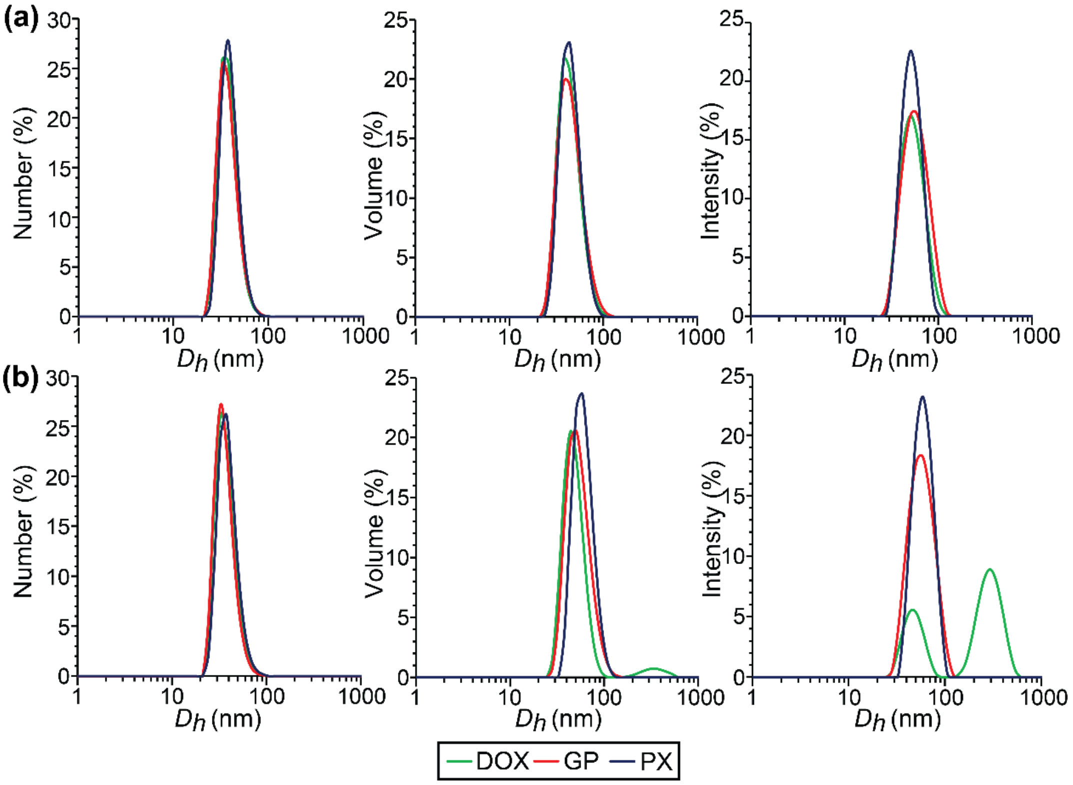

Figure S30: Number, volume and intensity PSDs for (a) PEG$_5$PS$_5$OVP$_{0.3}$D$_{0.1}$ and (b) PEG$_5$PS$_5$OVP$_{0.3}$D$_{0.2}$ micelles, as measured by DLS.

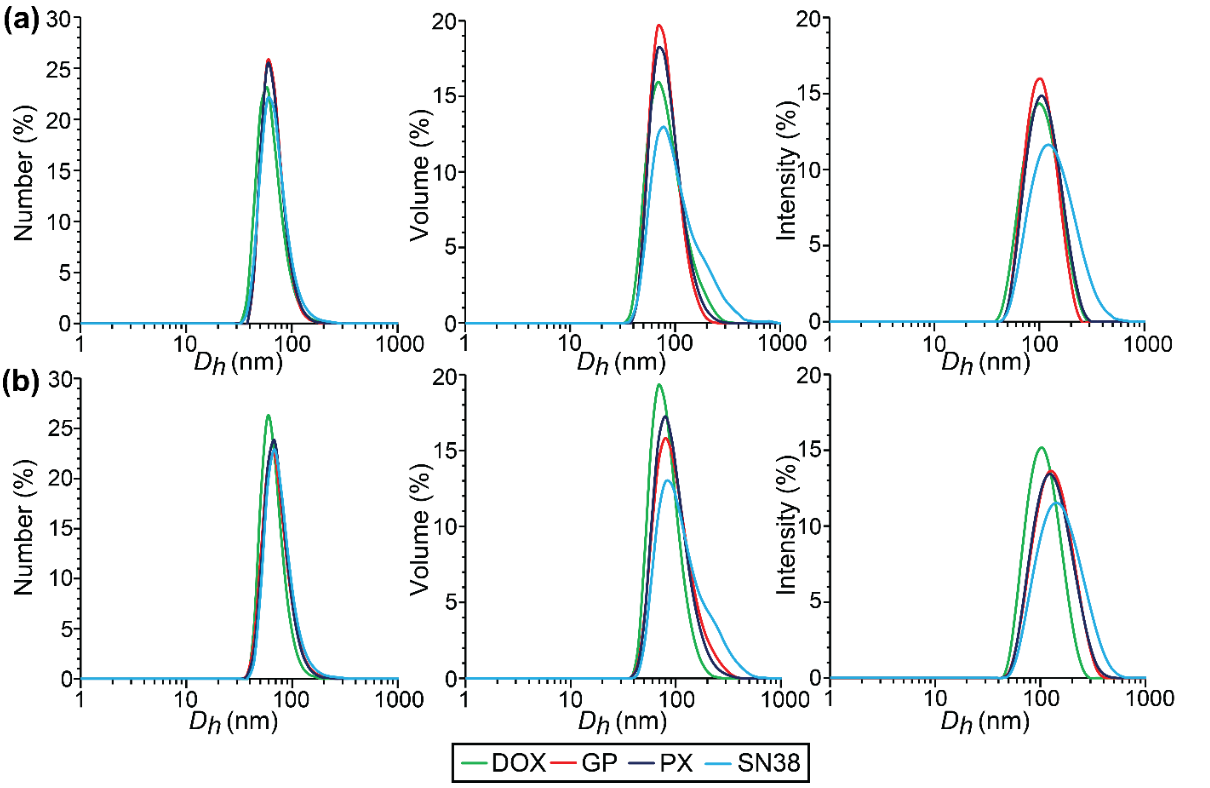

Figure S31: Number, volume and intensity PSDs for (a) $PEG_{14}PS_{12}D_{0.1}$ and (b) $PEG_{14}PS_{12}D_{0.2}$ micelles, as measured by DLS.

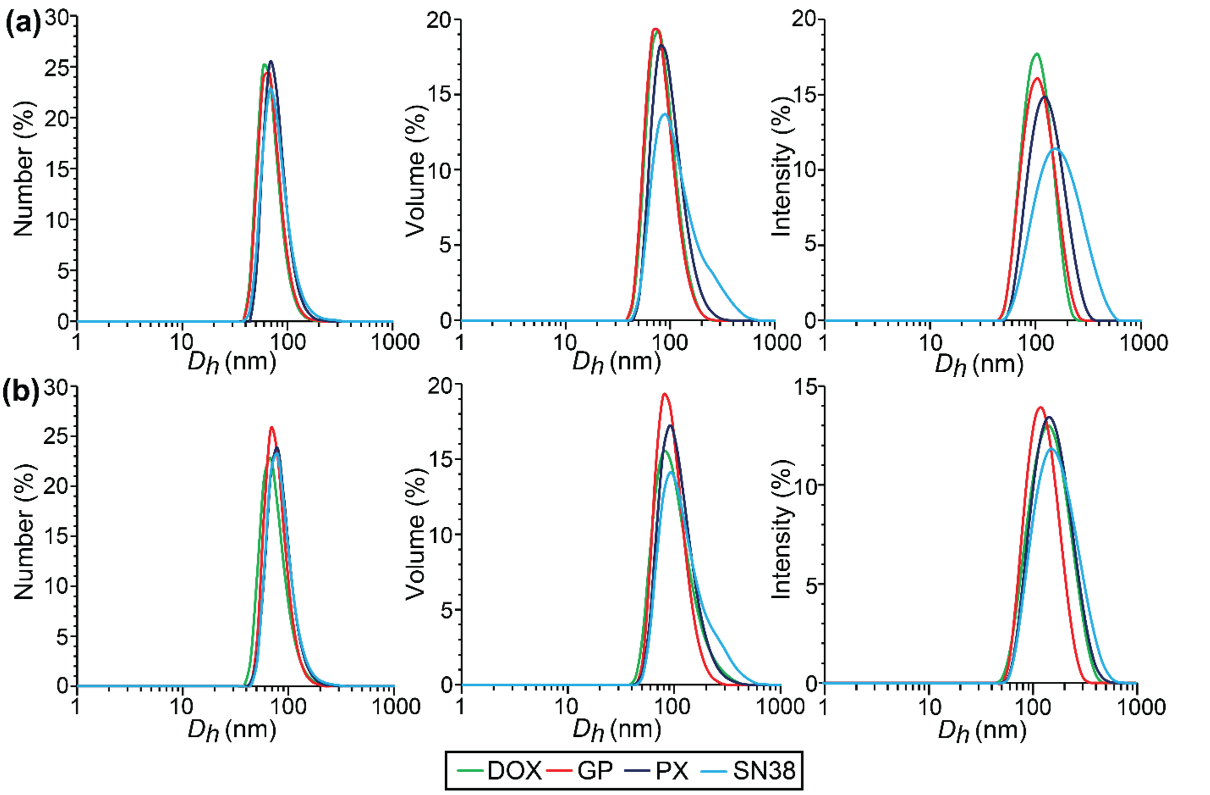

Figure S32: Number, volume and intensity PSDs for (a) $PEG_{14}PS_{12}OVP_{0.1}D_{0.1}$ and (b) $PEG_{14}PS_{12}OVP_{0.1}D_{0.2}$ micelles, as measured by DLS.

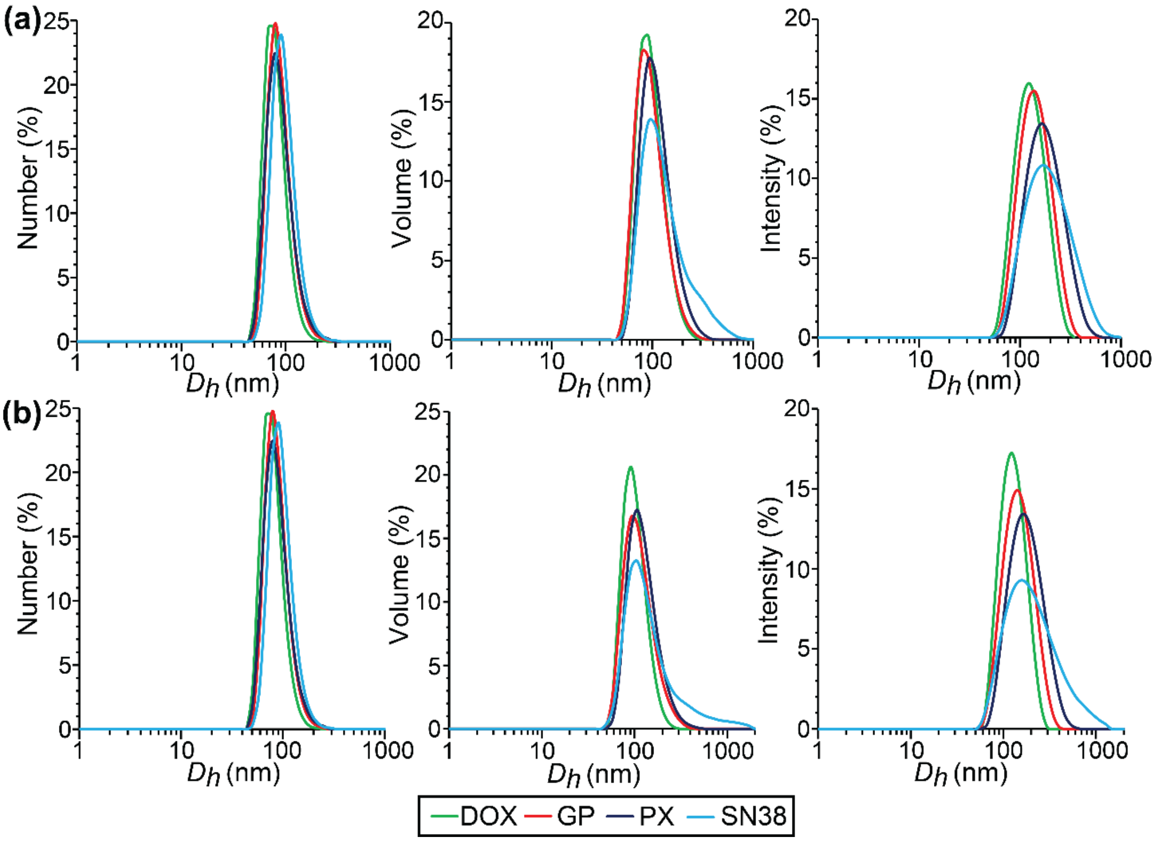

Figure S33: Number, volume and intensity PSDs for (a) $PEG_{14}PS_{12}OVP_{0.2}D_{0.1}$ and (b) $PEG_{14}PS_{12}OVP_{0.2}D_{0.2}$ micelles, as measured by DLS.

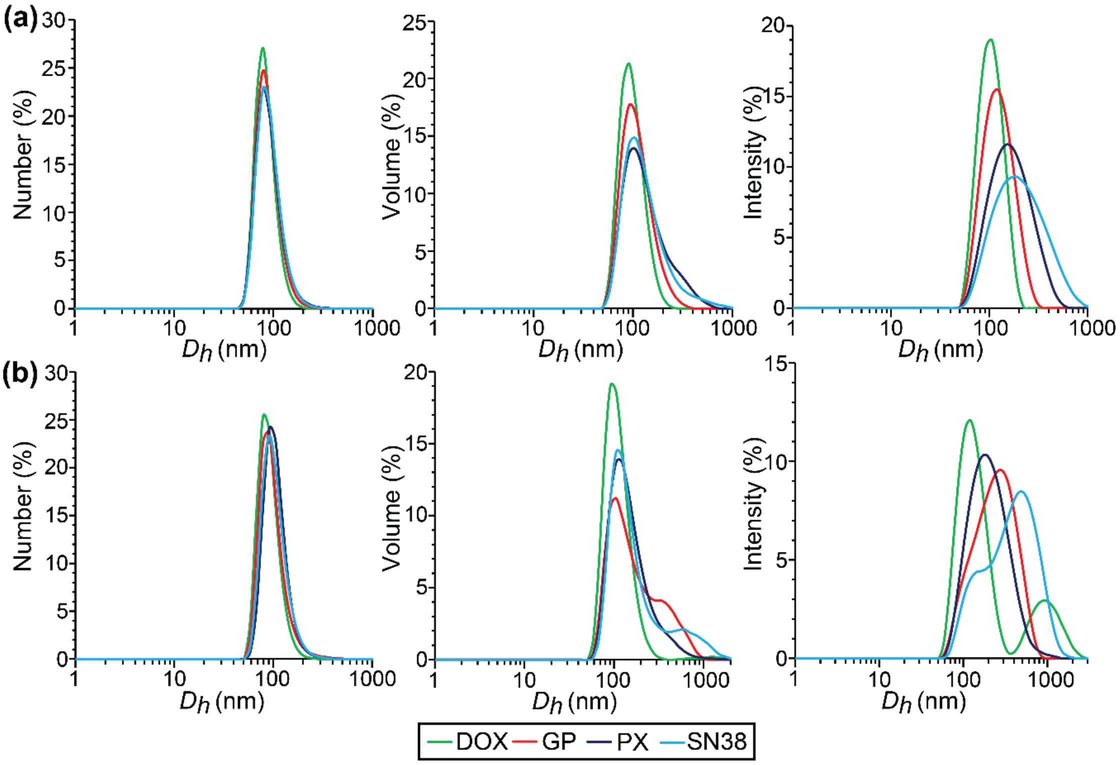

Figure S34: Number, volume and intensity PSDs for (a) $PEG_{14}PS_{12}OVP_{0.3}D_{0.1}$ and (b) $PEG_{14}PS_{12}OVP_{0.3}D_{0.2}$ micelles, as measured by DLS.

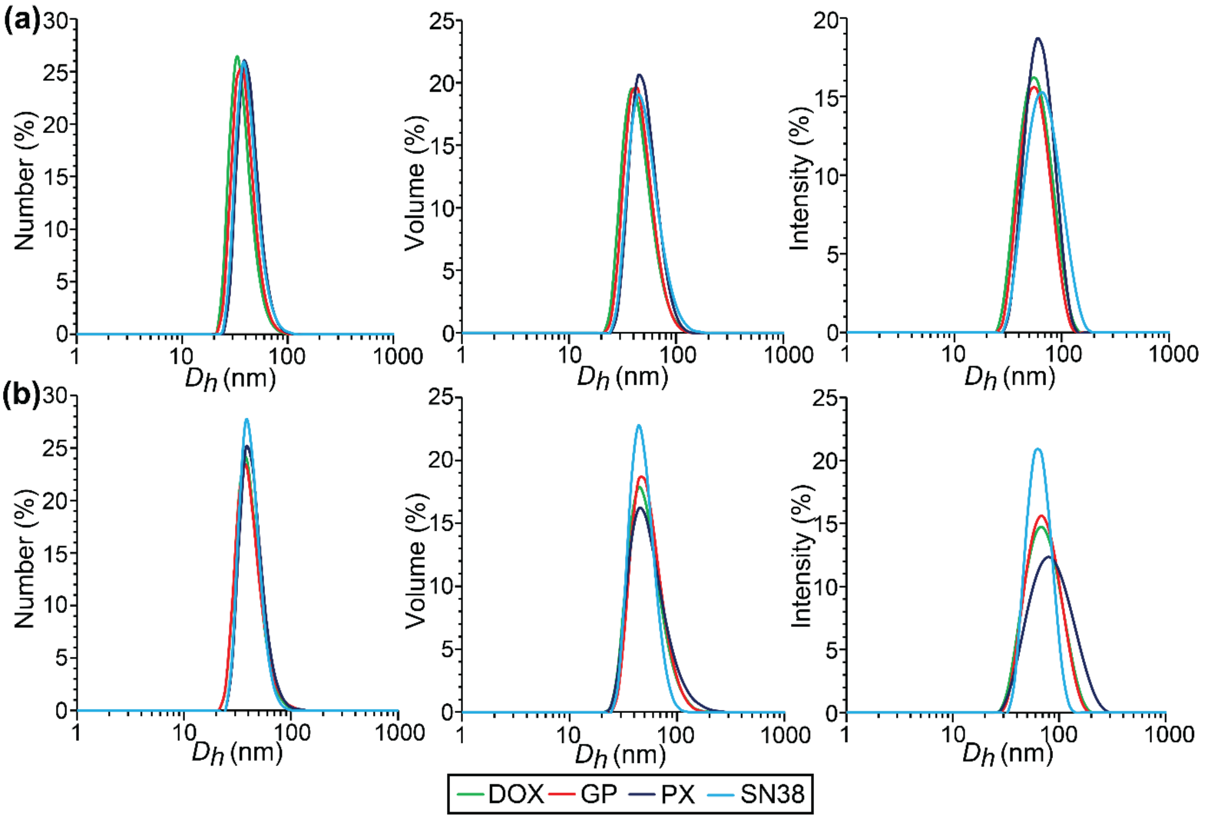

Figure S35: Number, volume and intensity PSDs for (a) $PEG_{10}PDL_{10}D_{0.1}$ and (b) $PEG_{10}PDL_{10}D_{0.2}$ micelles, as measured by DLS.

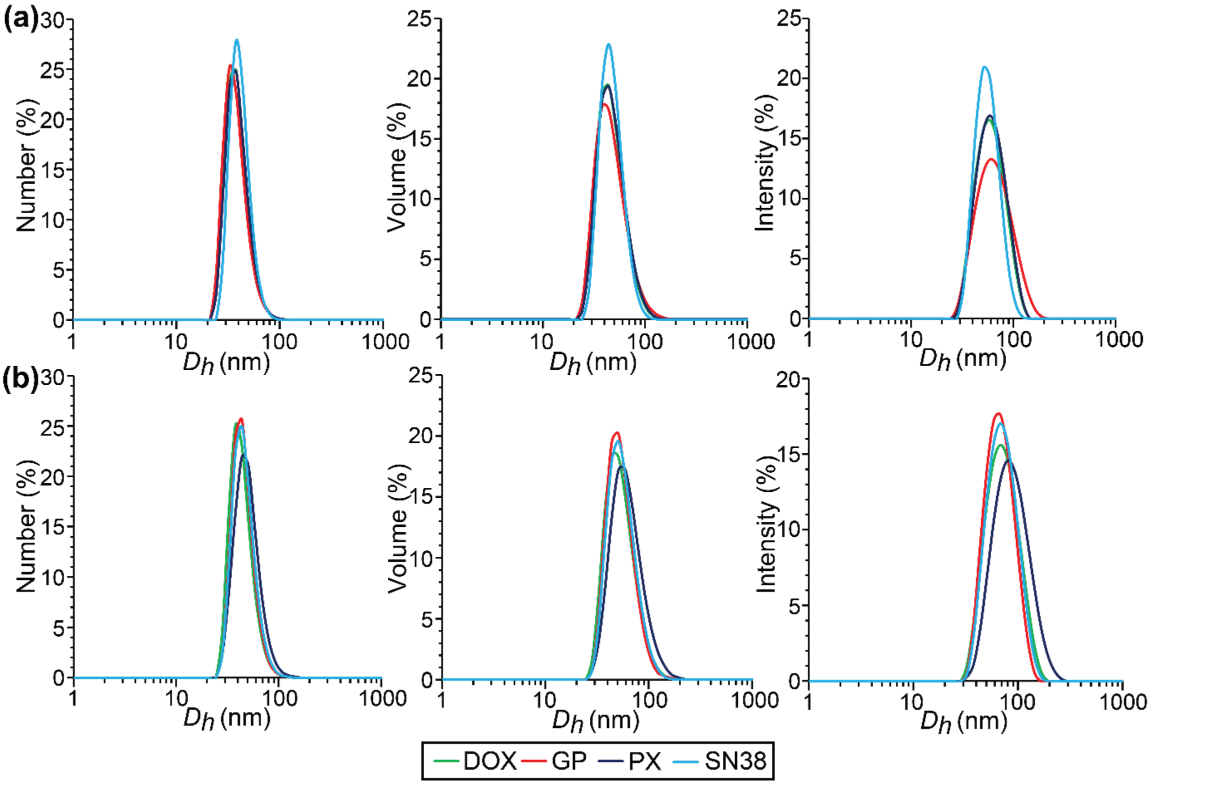

Figure S36: Number, volume and intensity PSDs for (a) $PEG_{10}PDL_{10}OVP_{0.1}D_{0.1}$ and (b) $PEG_{10}PDL_{10}OVP_{0.1}D_{0.2}$ micelles, as measured by DLS.

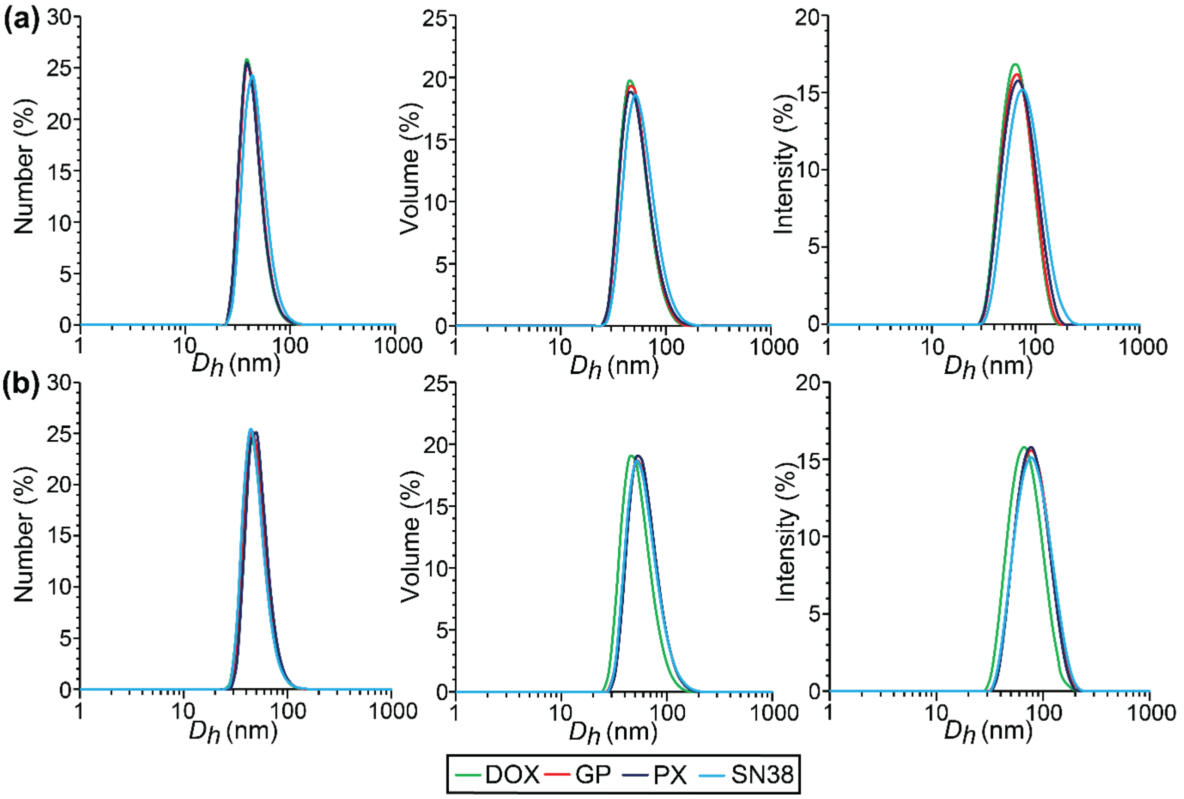

Figure S37: Number, volume and intensity PSDs for (a) $PEG_{10}PDL_{10}OVP_{0.2}D_{0.1}$ and (b) $PEG_{10}PDL_{10}OVP_{0.2}D_{0.2}$ micelles, as measured by DLS.

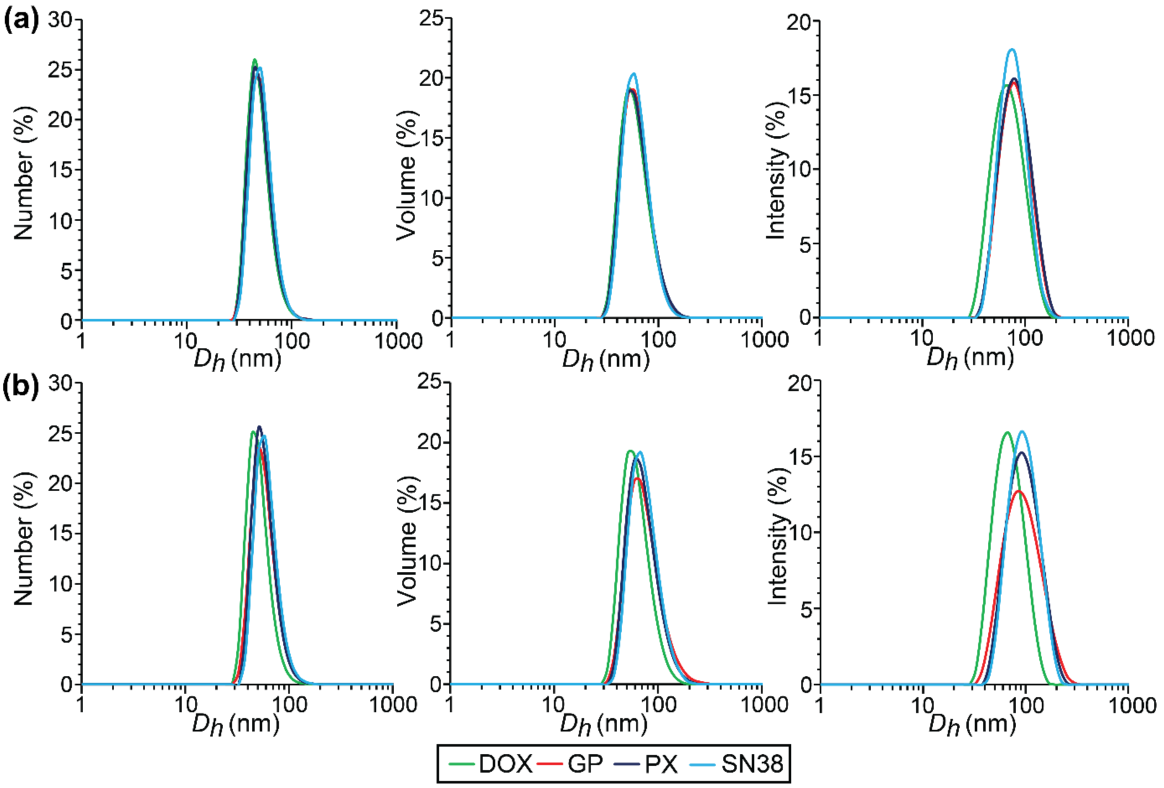

Figure S38: Number, volume and intensity PSDs for (a) $PEG_{10}PDL_{10}OVP_{0.3}D_{0.1}$ and (b) $PEG_{10}PDL_{10}OVP_{0.3}D_{0.2}$ micelles, as measured by DLS.

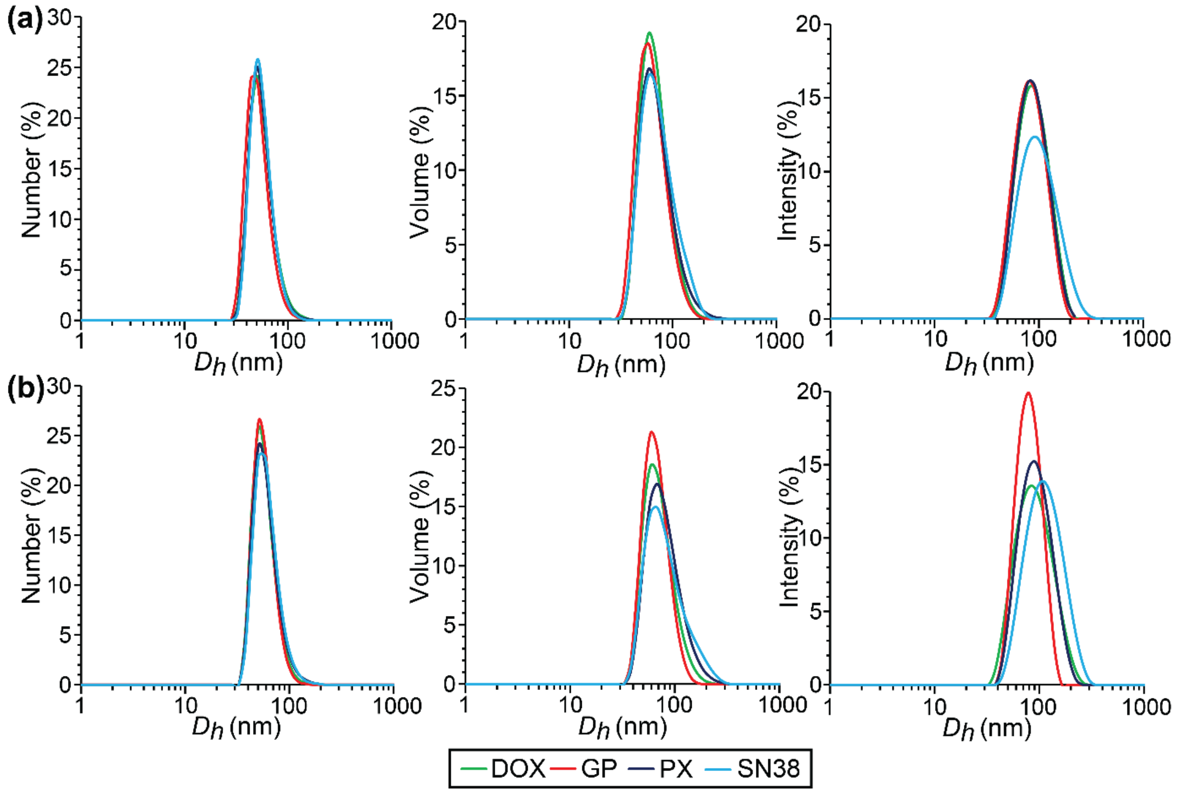

Figure S39: Number, volume and intensity PSDs for (a) $PEG_{10}PLA_{10}D_{0.1}$ and (b) $PEG_{10}PLA_{10}D_{0.2}$ micelles, as measured by DLS.

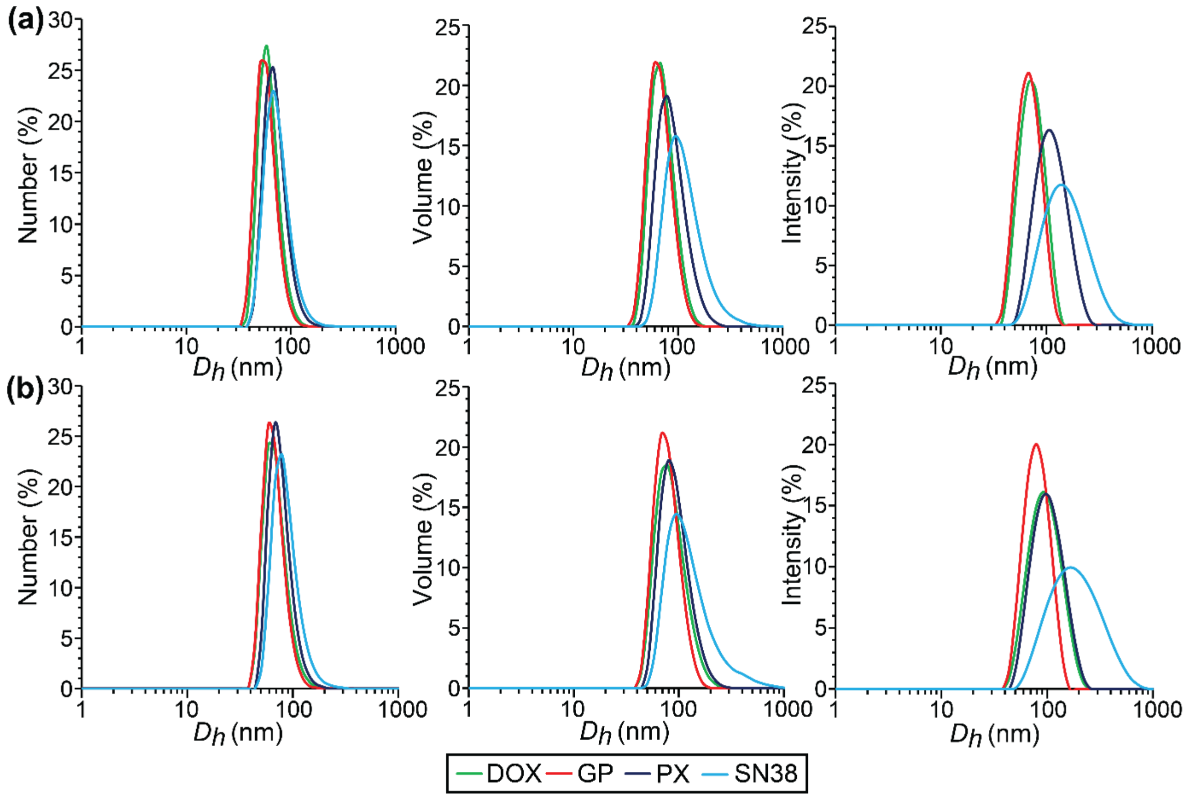

Figure S40: Number, volume and intensity PSDs for (a) $PEG_{10}PLA_{10}OVP_{0.1}D_{0.1}$ and (b) $PEG_{10}PLA_{10}OVP_{0.1}D_{0.2}$ micelles, as measured by DLS.

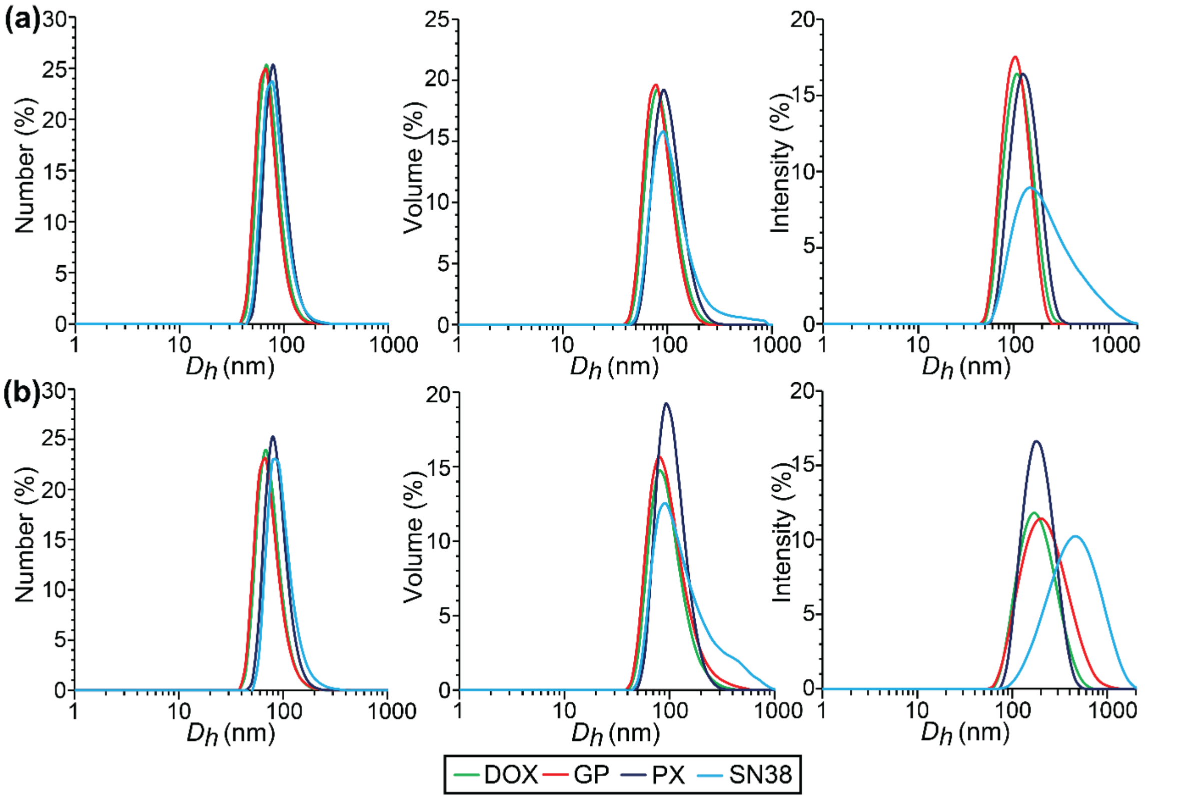

Figure S41: Number, volume and intensity PSDs for (a) PEG$_{10}$PLA$_{10}$OVP$_{0.2}$D$_{0.1}$ and (b) PEG$_{10}$PLA$_{10}$OVP$_{0.2}$D$_{0.2}$ micelles, as measured by DLS.

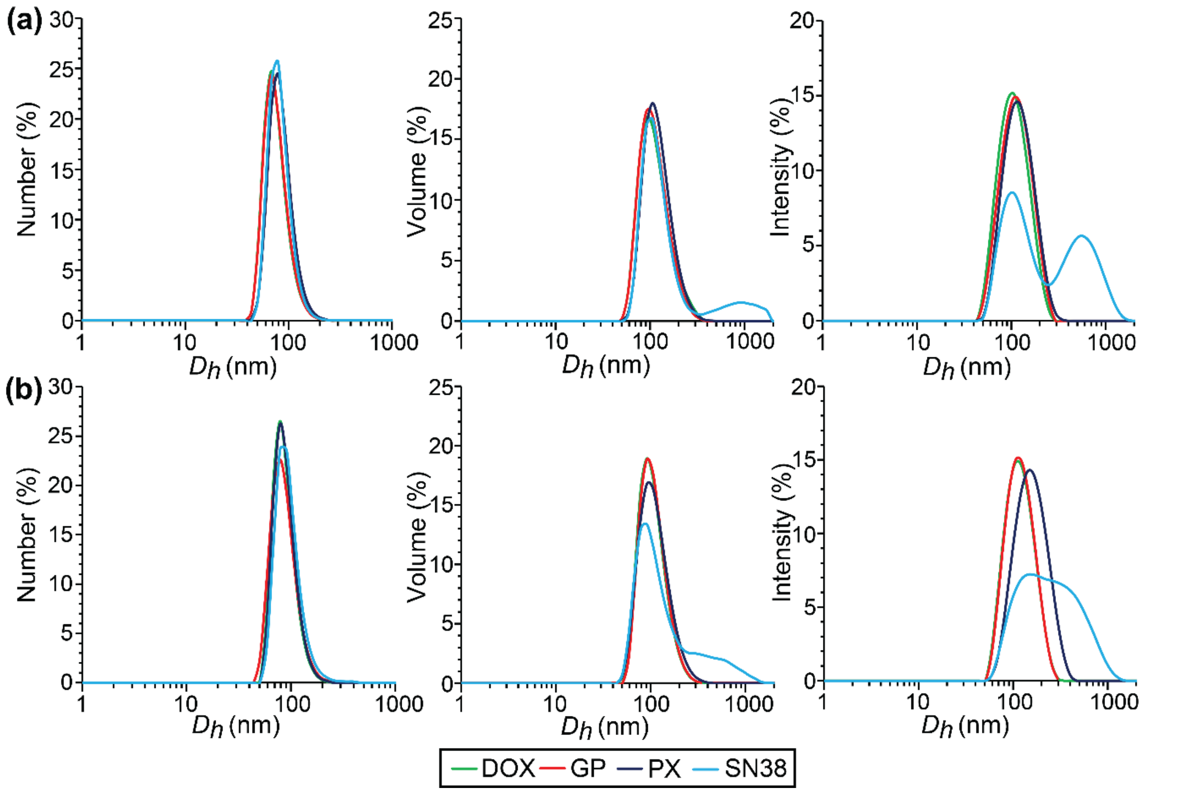

Figure S42: Number, volume and intensity PSDs for (a) PEG$_{10}$PLA$_{10}$OVP$_{0.3}$D$_{0.1}$ and (b) PEG$_{10}$PLA$_{10}$OVP$_{0.3}$D$_{0.2}$ micelles, as measured by DLS.

Table S14: PDI values of OVP- and drug-loaded (DOX, GP, PX and SN38) PEG_xHB_y micelles. All experiments were conducted in triplicate.

Polymers	DOX (PDI ± SD)		GP (PDI ± SD)		PX (PDI ± SD)		SN38 (PDI ± SD)	
	**$DOX_{0.1}$**	**$DOX_{0.2}$**	**$GP_{0.1}$**	**$GP_{0.1}$**	**$PX_{0.1}$**	**$PX_{0.2}$**	**$SN38_{0.1}$**	**$SN38_{0.2}$**
PEG_5PS_5	0.14 ± 0.01	0.15 ± 0.02	0.15 ± 0.01	0.17 ± 0.01	0.16 ± 0.01	0.18 ± 0.02	-	-
$PEG_5PS_5OVP_{0.1}$	0.15 ± 0.02	0.17 ± 0.01	0.16 ± 0.02	0.17 ± 0.01	0.17 ± 0.02	0.18 ± 0.01	-	-
$PEG_5PS_5OVP_{0.2}$	0.17 ± 0.01	0.18 ± 0.02	0.17 ± 0.01	0.18 ± 0.02	0.18 ± 0.02	0.19 ± 0.02	-	-
$PEG_5PS_5OVP_{0.3}$	0.20 ± 0.03	0.28 ± 0.04	0.20 ± 0.02	0.22 ± 0.02	0.21 ± 0.02	0.23 ± 0.01	-	-
$PEG_{14}PS_{12}$	0.17 ± 0.01	0.18 ± 0.01	0.16 ± 0.02	0.17 ± 0.02	0.15 ± 0.01	0.17 ± 0.02	0.21 ± 0.01	0.22 ± 0.01
$PEG_{14}PS_{12}OVP_{0.1}$	0.17 ± 0.01	0.18 ± 0.01	0.18 ± 0.01	0.20 ± 0.02	0.17 ± 0.01	0.19 ± 0.02	0.22 ± 0.01	0.24 ± 0.02
$PEG_{14}PS_{12}OVP_{0.2}$	0.18 ± 0.01	0.19 ± 0.02	0.19 ± 0.01	0.20 ± 0.01	0.18 ± 0.01	0.20 ± 0.02	0.23 ± 0.02	0.27 ± 0.03
$PEG_{14}PS_{12}OVP_{0.3}$	0.20 ± 0.01	0.25 ± 0.04	0.21 ± 0.02	0.29 ± 0.03	0.22 ± 0.01	0.26 ± 0.03	0.28 ± 0.02	0.31 ± 0.04
$PEG_{10}PDL_{10}$	0.13 ± 0.01	0.14 ± 0.02	0.15 ± 0.02	0.16 ± 0.01	0.16 ± 0.01	0.18 ± 0.01	0.15 ± 0.02	0.17 ± 0.01
$PEG_{10}PDL_{10}OVP_{0.1}$	0.15 ± 0.01	0.16 ± 0.01	0.18 ± 0.02	0.20 ± 0.01	0.17 ± 0.01	0.20 ± 0.01	0.17 ± 0.02	0.18 ± 0.02
$PEG_{10}PDL_{10}OVP_{0.2}$	0.17 ± 0.01	0.19 ± 0.01	0.19 ± 0.04	0.22 ± 0.02	0.19 ± 0.02	0.22 ± 0.02	0.19 ± 0.01	0.21 ± 0.03
$PEG_{10}PDL_{10}OVP_{0.3}$	0.19 ± 0.01	0.21 ± 0.02	0.22 ± 0.02	0.24 ± 0.02	0.21 ± 0.02	0.23 ± 0.02	0.21 ± 0.01	0.23 ± 0.02
$PEG_{10}PLA_{10}$	0.14 ± 0.01	0.17 ± 0.02	0.13 ± 0.01	0.15 ± 0.01	0.14 ± 0.02	0.17 ± 0.02	0.17 ± 0.02	0.19 ± 0.03
$PEG_{10}PLA_{10}OVP_{0.1}$	0.15 ± 0.01	0.17 ± 0.01	0.14 ± 0.01	0.15 ± 0.01	0.18 ± 0.01	0.21 ± 0.01	0.20 ± 0.02	0.23 ± 0.02
$PEG_{10}PLA_{10}OVP_{0.2}$	0.18 ± 0.02	0.19 ± 0.01	0.16 ± 0.01	0.18 ± 0.01	0.20 ± 0.04	0.23 ± 0.03	0.24 ± 0.01	0.27 ± 0.01
$PEG_{10}PLA_{10}OVP_{0.3}$	0.19 ± 0.02	0.22 ± 0.04	0.19 ± 0.01	0.21 ± 0.01	0.22 ± 0.01	0.25 ± 0.02	0.26 ± 0.01	0.33 ± 0.02

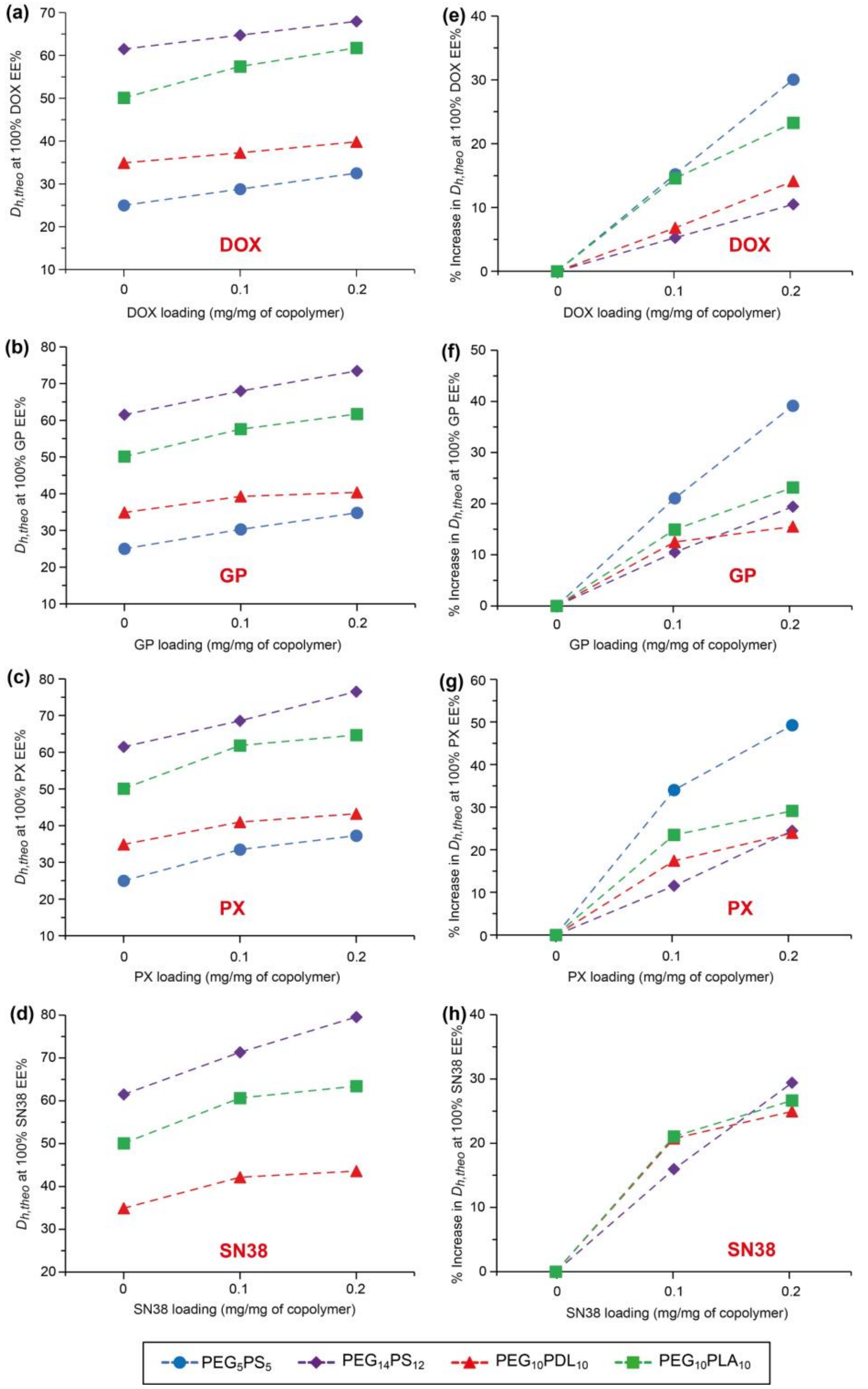

Figure S43: (**a–d**) $D_{h,theo}$ and (**e–h**) percentage increase in $D_{h,theo}$ relative to blank micelles at 100% drug (DOX, GP, SN38 and PX) EE% as a function of drug loading (0, 0.1 and 0.2 mg/mg of copolymer) for PEG_xHB_y micelles. Only symbols represent the experimental data; the dashed lines are guides to the eyes. $D_{h,theo}$ were analysed by assuming micelles are 100% loaded by drugs (DOX, GP, SN38 and PX) with or without OVP that extensively used for modelling purposes to visualize loading-dependent trends with an assumption of 100% EE that serves only as a comparative reference and does not imply complete OVP incorporation.

Table S15: $D_{h,theo}$ and percentage increase in $D_{h,theo}$ relative to blank micelles at 100% drug (DOX, GP, SN38 and PX) EE% as a function drug loading (0, 0.1 and 0.2 mg/mg of copolymer) for PEG_xHB_y micelles.

Drug and loading	PEG_5PS_5		$PEG_{14}PS_{12}$		$PEG_{10}PDL_{10}$		$PEG_{10}PLA_{10}$	
	$D_{h,theo}$[a] (nm)	$D_{h,theo}$[b] (%)	$D_{h,theo}$ (nm)	$D_{h,theo}$ (%)	$D_{h,theo}$ (nm)	$D_{h,theo}$ (%)	$D_{h,theo}$ (nm)	$D_{h,theo}$ (%)
Blank	25	0	62	0	35	0	50	0
$DOX_{0.1}$	29	15	65	5	37	7	57	15
$DOX_{0.2}$	33	30	68	10	40	14	62	23
$GP_{0.1}$	30	21	68	11	39	13	58	15
$GP_{0.2}$	35	39	73	19	40	16	62	23
$PX_{0.1}$	34	34	69	12	41	17	62	23
$PX_{0.2}$	37	49	77	24	43	24	65	29
$SN38_{0.1}$	-	-	71	16	42	21	61	21
$SN38_{0.2}$	-	-	80	29	44	25	63	27

[a] $D_{h,theo}$ represents theoretical hydrodynamic diameter of the micelles at a drug EE% of 100%, calculated relative to the experimentally determined D_h of the blank micelles and drug-loaded micelles determined from DLS number PSDs. [b] Percentage increase in $D_{h,theo}$ of the drug-loaded micelles relative to the D_h of the blank micelles.

Table S16: Concentration and volume of stock solutions combined to prepare micelle solutions in PBS for the drug (DOX, GP, PX and SN38) release studies for blank and OVP-loaded (0.1, 0.2 and 0.3 mg/mg of copolymer) PEG_xHB_y micelles.

Micelle composition[a]	Solvent used for copolymer, OVP and drug stock solutions	Conc. of copolymer stock solution (mg/L)	Volume of copolymer stock solution (μL)	OVP mass fraction (mg/mg of copolymer)	Conc. of OVP stock solution (mg/L)	Volume of OVP stock solution (μL)	Drug mass fraction (mg/mg of copolymer)	Conc. of drug stock solution (mg/L)	Volume of drug stock solution added (μL)	Volume of PBS added (10 mM, pH 7.4) (μL)
$PEG_xHB_yD_{0.1}$	Acetone	1000	6000	-	-	-	0.1	1000	600	6000
$PEG_xHB_ySN38_{0.1}$	THF	1000	6000	-	-	-	0.1	1000	600	6000
$PEG_xHB_yOVP_{0.1}D_{0.1}$	Acetone	1000	6000	0.1	1000	600	0.1	1000	600	6000
$PEG_xHB_yOVP_{0.2}D_{0.1}$	Acetone	1000	6000	0.2	1000	1200	0.1	1000	600	6000
$PEG_xHB_yOVP_{0.3}D_{0.1}$	Acetone	1000	6000	0.3	1000	1800	0.1	1000	600	6000
$PEG_xHB_yOVP_{0.1}SN38_{0.1}$	THF	1000	6000	0.1	1000	600	0.1	1000	600	6000
$PEG_xHB_yOVP_{0.2}SN38_{0.1}$	THF	1000	6000	0.2	1000	1200	0.1	1000	600	6000
$PEG_xHB_yOVP_{0.3}SN38_{0.1}$	THF	1000	6000	0.3	1000	1800	0.1	1000	600	6000

[a] Micellar solutions were prepared in PBS via the solvent evaporation approach. Stock solutions of all polymers and drugs (D; DOX, GP and PX) were prepared in acetone, however, for SN38, stocks of polymers and SN38 was prepared in THF. HB stands for hydrophobic block and represents PS, PDL and PLA.

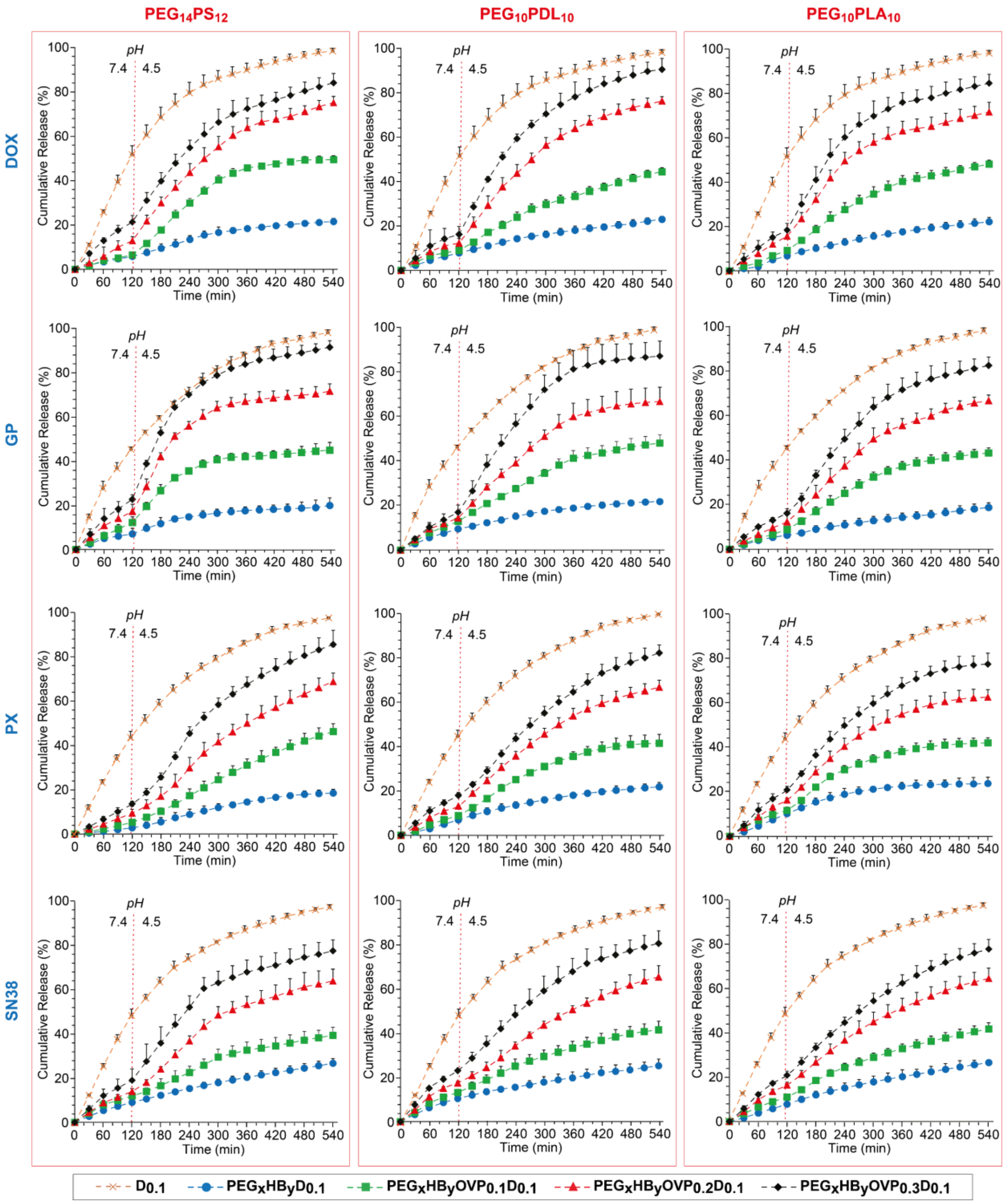

Figure S44: Summary of drug release profiles for different copolymer micelles with and without OVP. *In vitro* dialysis assay showing the cumulative release of DOX, GP, PX and SN38 (all 0.1 mg/mg of copolymer) from free drug solutions and PEG_xHB_y micelles with various OVP loadings (0, 0.1, 0.2 and 0.3 mg/mg of copolymer) against PBS (pH 7.4) for the first 2 h, followed by acetate buffer (pH 4.5) for 7 h; experiments were conducted at 22 ± 1 °C and the drug release was measured *via* UV-vis spectrophotometry (DOX, GP and SN38) or HPLC (PX). Data are shown as the average cumulative release (%) + SD (n = 3). Only symbols represent the experimental data; the dashed lines are guides to the eyes.

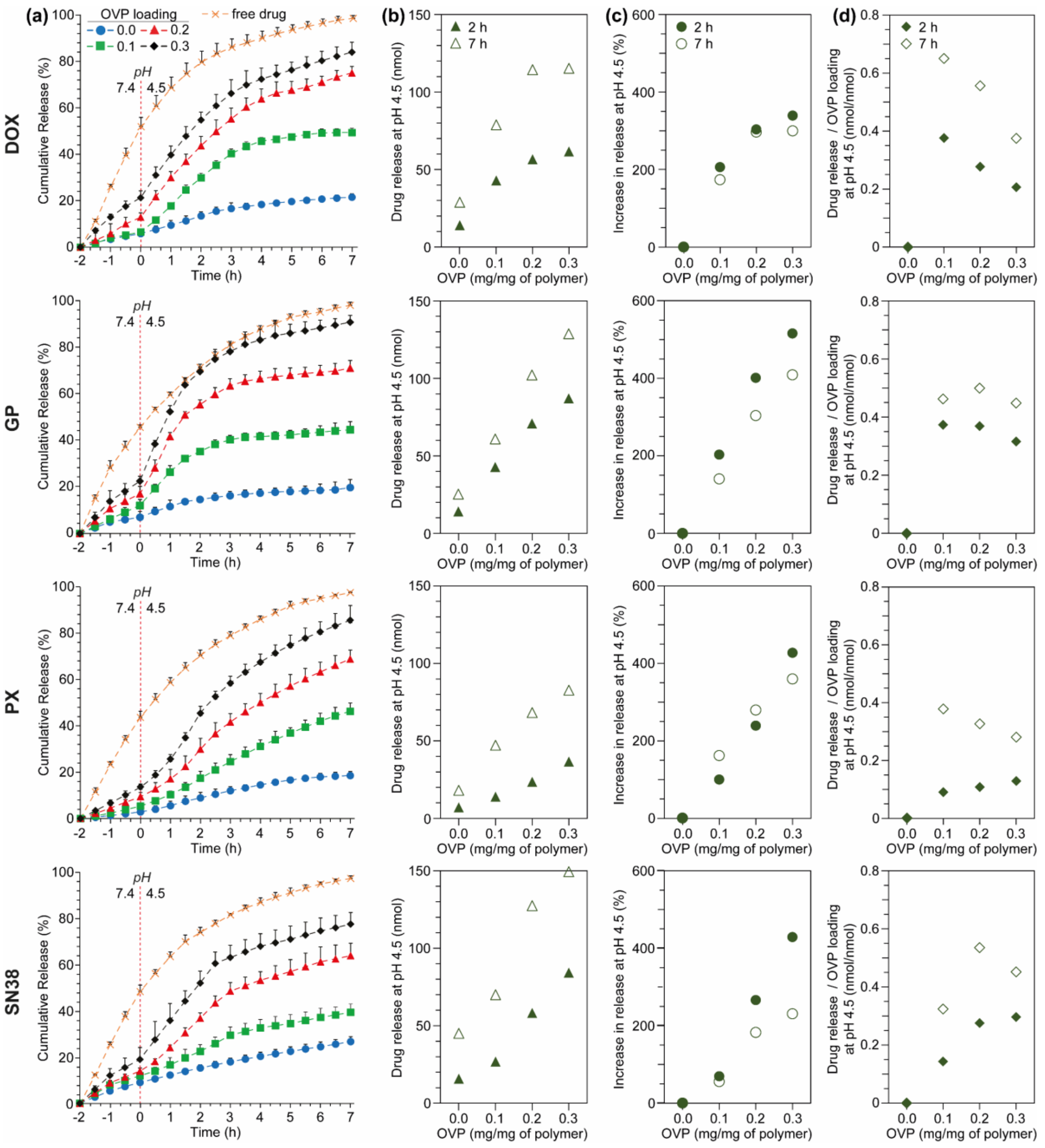

Figure S45: pH-dependent drug release from $PEG_{14}PS_{12}$ micelles. Rows correspond to the respective drugs indicated on the left-hand side. **(a)** *In vitro* dialysis assay showing the cumulative release of drugs (all 0.1 mg/mg of copolymer) from free drug solutions and micelles with various OVP loadings (0, 0.1, 0.2 and 0.3 mg/mg of copolymer) against PBS (pH 7.4) for several hours (-2 to 0 h), followed by acetate buffer (pH 4.5) for 7 h (0 to 7 h) at 22 ± 1 °C. Data are shown as the average cumulative release (%) + SD (n = 3). Only symbols represent the experimental data; the dashed lines are guides to the eyes. Release of the drugs at pH 4.5 after 2 and 7 h was analysed with respect to the initial OVP loading (0, 0.1, 0.2 and 0.3 mg/mg of copolymer) as **(b)** the mole amount of drug released, **(c)** the percentage increase in drug release relative to micelles without OVP, and **(c)** the amount of drug released relative to the amount of OVP loaded (nmol/nmol).

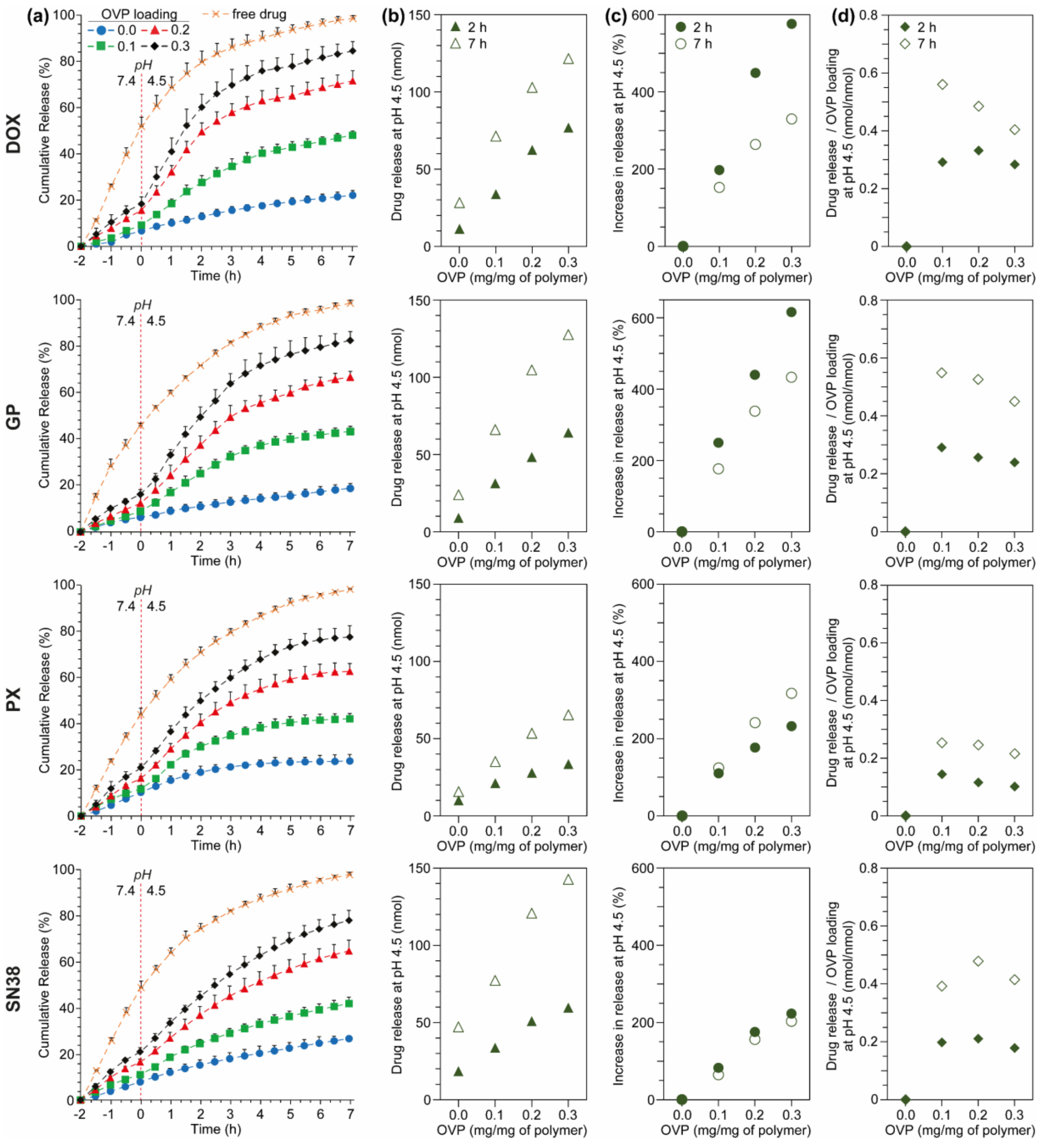

Figure S46: pH-dependent drug release from $PEG_{10}PLA_{10}$ micelles. Rows correspond to the respective drugs indicated on the left-hand side. **(a)** *In vitro* dialysis assay showing the cumulative release of drugs (all 0.1 mg/mg of copolymer) from free drug solutions and micelles with various OVP loadings (0, 0.1, 0.2 and 0.3 mg/mg of copolymer) against PBS (pH 7.4) for several hours (-2 to 0 h), followed by acetate buffer (pH 4.5) for 7 h (0 to 7 h) at 22 ± 1 °C. Data are shown as the average cumulative release (%) + SD (n = 3). Only symbols represent the experimental data; the dashed lines are guides to the eyes. Release of the drugs at pH 4.5 after 2 and 7 h was analysed with respect to the OVP loading (0, 0.1, 0.2 and 0.3 mg/mg of copolymer) as **(b)** the mole amount of drug released, **(c)** the percentage increase in drug release relative to micelles without OVP, and **(c)** the amount of drug released relative to the amount of OVP loaded (nmol/nmol).

Table S17: Model fitting for the prediction of release behaviour for the release of free drug (DOX, GP, PX and SN38: equivalent to 0.1 mg/mg of copolymer) across the dialysis membrane (MWCO = 100 kDa).

**Drug**	**Release models, exponents and goodness-of-fit**[a]									
	First order			Higuchi			Korsmeyer-Peppas			
	R^2	MSE	MSC	R^2	MSE	MSC	n	R^2	MSE	MSC
$DOX_{0.1}$	0.99	5.65	**4.66**	0.95	43.04	2.63	**0.49**	0.95	45.26	2.53
$GP_{0.1}$	1.00	3.98	**4.96**	0.98	15.47	3.60	**0.53**	0.98	14.92	3.59
$PX_{0.1}$	0.99	6.73	**4.52**	0.97	24.02	3.25	**0.57**	0.98	19.28	3.42
$SN38_{0.1}$	1.00	1.79	**5.75**	0.97	24.54	3.14	**0.51**	0.97	25.80	3.04

[a] All modelling was conducted using the DDSolver plugin for excel [5]. For each model, the coefficient of determination (R^2), mean square error (MSE) and model selection criterion (MSC) were calculated to determine the goodness-of-fit, and therefore, the most appropriate model for describing the release behaviour. An R^2 value closer to 1 indicates a closer correlation between the response values and the predicted response values. A MSE value closer to 0 indicates a closer correlation between the response values and the predicted response values. The MSC is a modified reciprocal form of the Akaike information criterion (AIC), which is normalized to be independent of the scaling of the data points. A larger MSC indicates a closer correlation between the response values and the predicted response values. The MSC was used to determine the best model fit to the release curves for each sample, as shown by the green color coding. Dark blue color coding for the release exponent corresponds to anomalous non-Fickian diffusion ($0.45<n<0.85$) [6,7].

Table S18: Model fitting for the prediction of release behaviour for the release of drugs (DOX, GP, PX and SN38: 0.1 mg/mg of copolymer) from blank and OVP-loaded (0.1, 0.2 and 0.3 mg/mg of copolymer) PEG_xHB_y micelles in PBS (pH 7.4) and acetate buffer (pH 4.5).

Release models, exponents and goodness-of-fit[a]																				
Doxorubicin (DOX)																				
	First order						Higuchi						Korsmeyer-Peppas							
	R^2		MSE		MSC		R^2		MSE		MSC		n		R^2		MSE		MSC	
Sample pH	7.4	4.5	7.4	4.5	7.4	4.5	7.4	4.5	7.4	4.5	7.4	4.5	7.4	4.5	7.4	4.5	7.4	4.5	7.4	4.5
PEG_5PS_5	0.98	0.95	0.49	1.39	3.33	2.52	0.84	0.96	4.99	1.08	1.02	2.78	**1.18**	**0.69**	1.00	1.00	0.01	0.12	**6.84**	**4.93**
PEG_5PS_5[b]	0.89		9.65		1.83		0.97		2.83		3.06		**0.57**		0.98		2.03		**3.44**	
$PEG_5PS_5OVP_{0.3}$	0.99	0.94	1.29	10.02	3.53	2.32	0.86	0.98	14.19	3.73	1.13	3.31	**1.11**	**0.60**	1.00	0.99	0.32	1.66	**4.8**	**4.06**
$PEG_{14}PS_{12}$	0.99	0.90	3.20	2.76	0.07	1.82	0.96	0.95	0.23	1.21	2.06	2.64	**0.79**	**0.97**	0.63	0.97	0.00	0.72	**7.32**	**3.11**
$PEG_{14}PS_{12}$[b]	0.96		1.97		2.98		0.91		4.73		2.11		**0.77**		0.97		1.38		**3.29**	
$PEG_{14}PS_{12}OVP_{0.1}$	0.99	0.89	3.33	23.26	0.10	1.76	0.92	0.94	0.55	12.82	1.58	2.32	**0.88**	**0.94**	0.56	0.94	0.10	12.50	**3.22**	**2.36**
$PEG_{14}PS_{12}OVP_{0.2}$	0.99	0.97	4.00	11.56	0.21	3.05	0.87	0.97	3.52	12.57	1.18	2.96	**1.09**	**0.98**	0.59	0.98	0.11	8.69	**4.50**	**3.27**
$PEG_{14}PS_{12}OVP_{0.3}$	0.99	0.96	3.39	17.11	0.83	2.60	0.96	0.98	2.74	9.57	2.19	3.18	**0.76**	**0.98**	0.55	0.98	0.08	8.56	**5.58**	**3.23**
$PEG_{10}PDL_{10}$	0.99	0.97	0.08	0.89	4.03	2.95	0.94	0.95	0.73	1.37	1.78	2.52	**0.85**	**0.73**	1.00	1.00	0.02	0.10	**5.14**	**5.08**
$PEG_{10}PDL_{10}$[b]	0.96		2.57		2.77		0.96		2.51		2.79		**0.69**		0.99		0.34		**4.75**	
$PEG_{10}PDL_{10}OVP_{0.1}$	0.92	0.98	1.21	2.74	1.39	3.57	0.98	0.95	0.34	7.42	2.65	2.57	**0.61**	**0.72**	0.98	0.99	0.28	0.75	**2.75**	**4.81**
$PEG_{10}PDL_{10}OVP_{0.2}$	0.96	0.99	1.36	6.17	1.99	**3.94**	0.97	0.97	0.93	16.87	2.36	2.94	**0.68**	**0.61**	0.99	0.98	0.43	10.21	**3.01**	3.38
$PEG_{10}PDL_{10}OVP_{0.3}$	0.97	0.99	1.74	8.03	2.31	**3.84**	0.96	0.98	1.84	13.54	2.25	3.32	**0.70**	**0.52**	0.99	0.98	0.69	14.15	**3.12**	3.21
$PEG_{10}PLA_{10}$	0.93	0.96	0.58	0.84	1.91	2.93	0.74	0.95	2.08	1.30	0.64	2.50	**1.52**	**0.73**	0.97	1.00	0.21	0.11	**2.81**	**4.92**
$PEG_{10}PLA_{10}$[b]	0.98		1.25		3.55		0.91		4.58		2.15		**0.79**		0.99		0.80		**3.85**	
$PEG_{10}PLA_{10}OVP_{0.1}$	0.98	0.95	0.27	8.38	3.10	2.48	0.84	0.96	2.24	6.27	0.99	2.77	**1.21**	**0.64**	0.99	0.98	0.11	3.09	**3.93**	**3.42**
$PEG_{10}PLA_{10}OVP_{0.2}$	1.00	0.90	0.08	30.86	**5.25**	1.77	0.92	0.96	3.06	11.66	1.54	**2.75**	**0.93**	**0.51**	1.00	0.96	0.08	12.47	5.08	2.62
$PEG_{10}PLA_{10}OVP_{0.3}$	1.00	0.91	0.22	37.83	4.54	1.82	0.93	0.96	3.60	15.66	1.73	**2.70**	**0.86**	**0.47**	1.00	0.96	0.18	16.21	**4.60**	2.61

Table continued on next page

Gossypol (GP)

	First order						Higuchi						Korsmeyer-Peppas							
	R^2		MSE		MSC		R^2		MSE		MSC		n		R^2		MSE		MSC	
Sample *pH*	7.4	4.5	7.4	4.5	7.4	4.5	7.4	4.5	7.4	4.5	7.4	4.5	7.4	4.5	7.4	4.5	7.4	4.5	7.4	4.5
PEG_5PS_5	0.99	0.91	0.18	2.85	4.34	1.94	0.93	0.96	2.43	1.18	1.74	2.82	**0.86**	**0.63**	0.99	0.98	0.12	0.56	**4.63**	**3.50**
$PEG_5PS_5{}^b$	0.86		13.45		1.56		0.98		1.90		**3.51**		**0.54**		0.98		1.73		**3.56**	
$PEG_5PS_5OVP_{0.3}$	0.99	0.88	0.42	15.15	4.32	1.56	0.89	0.98	8.54	2.64	1.31	**3.85**	**0.99**	**0.54**	0.99	0.98	0.43	2.39	**4.18**	3.35
$PEG_{14}PS_{12}$	0.94	0.67	0.43	4.17	1.72	0.49	0.97	0.97	0.21	0.33	2.43	1.02	**0.66**	**0.46**	0.98	0.98	0.11	0.31	**2.94**	**3.02**
$PEG_{14}PS_{12}{}^b$	0.84		5.56		1.45		0.96		1.58		2.72		**0.58**		0.96		1.36		**2.81**	
$PEG_{14}PS_{12}OVP_{0.1}$	1.00	0.56	0.00	39.21	8.50	0.12	0.90	0.91	2.01	7.80	1.43	1.73	**0.98**	**0.38**	1.00	0.94	0.00	5.30	**9.24**	**2.06**
$PEG_{14}PS_{12}OVP_{0.2}$	0.98	0.71	0.66	73.04	3.12	0.53	0.96	0.91	1.90	21.39	2.06	1.76	**0.77**	**0.38**	0.99	0.94	0.23	14.93	**4.08**	**2.06**
$PEG_{14}PS_{12}OVP_{0.3}$	0.99	0.83	0.72	65.84	3.59	1.05	0.95	0.94	3.57	22.90	1.99	2.11	**0.79**	**0.39**	1.00	0.96	0.32	14.71	**4.30**	**2.49**
$PEG_{10}PDL_{10}$	0.99	0.93	0.12	1.16	3.75	2.21	0.94	0.95	0.82	0.83	1.84	2.54	**0.83**	**0.68**	1.00	0.98	0.03	0.29	**4.89**	**3.54**
$PEG_{10}PDL_{10}{}^b$	0.88		5.49		1.68		0.99		0.95		3.44		**0.58**		0.99		0.57		**3.91**	
$PEG_{10}PDL_{10}OVP_{0.1}$	0.99	0.96	0.28	4.89	3.50	2.90	0.95	0.94	1.36	7.92	1.92	2.42	**0.81**	**0.70**	1.00	0.98	0.09	2.77	**4.50**	**3.41**
$PEG_{10}PDL_{10}OVP_{0.2}$	0.97	0.96	0.86	11.16	2.57	**2.81**	0.96	0.95	1.34	15.61	2.13	2.53	**0.74**	**0.62**	0.99	0.96	0.34	13.80	**3.39**	2.66
$PEG_{10}PDL_{10}OVP_{0.3}$	0.99	0.96	0.44	22.03	3.60	**2.71**	0.95	0.95	2.33	27.48	1.93	2.49	**0.81**	**0.54**	1.00	0.95	0.18	28.44	**4.40**	2.39
$PEG_{10}PLA_{10}$	0.97	0.98	0.16	0.30	2.64	3.49	0.95	0.93	0.31	1.04	2.01	2.26	**0.77**	**0.79**	0.99	1.00	0.06	0.04	**3.49**	**5.53**
$PEG_{10}PLA_{10}{}^b$	0.95		1.42		2.74		0.96		1.33		2.80		**0.70**		1.00		0.07		**5.65**	
$PEG_{10}PLA_{10}OVP_{0.1}$	1.00	0.94	0.02	8.61	5.44	2.29	0.92	0.95	0.94	6.67	1.59	2.55	**0.92**	**0.64**	1.00	0.97	0.01	3.93	**6.15**	**3.02**
$PEG_{10}PLA_{10}OVP_{0.2}$	1.00	0.97	0.08	8.41	4.62	**3.19**	0.94	0.95	1.44	16.35	1.77	2.56	**0.86**	**0.65**	1.00	0.97	0.00	8.66	**7.85**	**3.16**
$PEG_{10}PLA_{10}OVP_{0.3}$	0.98	0.98	0.79	11.76	2.85	**3.26**	0.97	0.95	1.34	22.87	2.31	2.60	**0.74**	**0.61**	1.00	0.96	0.08	17.19	**5.08**	2.83

Table continued on next page

Paclitaxel (PX)

Sample	First order						Higuchi						Korsmeyer-Peppas							
	R^2		MSE		MSC		R^2		MSE		MSC		n		R^2		MSE		MSC	
pH	7.4	4.5	7.4	4.5	7.4	4.5	7.4	4.5	7.4	4.5	7.4	4.5	7.4	4.5	7.4	4.5	7.4	4.5	7.4	4.5
PEG$_{14}$PS$_{12}$	0.99	0.97	0.01	0.86	4.13	3.12	0.86	0.91	0.18	2.43	1.10	2.08	1.15	0.78	1.00	0.98	0.00	0.50	6.50	3.59
PEG$_{14}$PS$_{12}$[b]	0.98		1.06		3.48		0.81		8.28		1.42		1.06		0.98		0.97		3.52	
PEG$_{14}$PS$_{12}$OVP$_{0.1}$	0.99	0.99	0.04	1.30	3.82	4.62	0.85	0.86	0.66	25.35	1.06	1.65	1.18	0.97	1.00	1.00	0.00	0.91	7.10	4.92
PEG$_{14}$PS$_{12}$OVP$_{0.2}$	1.00	0.99	0.03	3.44	5.24	4.38	0.88	0.89	1.60	42.13	1.26	1.87	1.06	0.86	1.00	0.99	0.00	5.23	9.96	3.90
PEG$_{14}$PS$_{12}$OVP$_{0.3}$	1.00	0.99	0.03	4.36	5.93	4.46	0.90	0.93	2.91	39.52	1.35	2.26	1.01	0.73	1.00	0.98	0.00	12.77	8.44	3.33
PEG$_{10}$PDL$_{10}$	0.99	0.94	0.07	1.39	3.83	2.30	0.85	0.96	1.08	0.80	1.08	2.85	1.16	0.67	1.00	0.99	0.01	0.14	5.91	4.55
PEG$_{10}$PDL$_{10}$[b]	0.96		1.86		2.97		0.93		3.46		2.35		0.74		0.98		0.80		3.77	
PEG$_{10}$PDL$_{10}$OVP$_{0.1}$	1.00	0.93	0.05	8.02	4.58	2.24	0.89	0.95	1.40	5.54	1.32	2.61	1.00	0.64	0.99	0.97	0.07	3.20	4.17	3.10
PEG$_{10}$PDL$_{10}$OVP$_{0.2}$	0.99	0.99	0.27	3.12	3.64	4.16	0.94	0.94	1.72	16.88	1.77	2.47	0.84	0.71	0.99	0.99	0.18	3.96	3.90	3.86
PEG$_{10}$PDL$_{10}$OVP$_{0.3}$	0.99	1.00	0.54	1.97	3.50	5.05	0.95	0.92	2.46	35.00	1.97	2.17	0.80	0.77	1.00	0.99	0.21	6.41	4.34	3.81
PEG$_{10}$PLA$_{10}$	0.99	0.65	0.15	6.29	3.88	0.45	0.85	0.96	2.39	0.79	1.09	2.51	1.15	0.45	1.00	0.96	0.00	0.80	7.90	2.47
PEG$_{10}$PLA$_{10}$[b]	0.83		10.57		1.41		0.94		3.94		2.39		0.57		0.94		3.73		2.40	
PEG$_{10}$PLA$_{10}$OVP$_{0.1}$	0.98	0.81	0.44	17.84	2.79	1.11	0.96	0.97	0.78	3.08	2.23	2.87	0.75	0.51	1.00	0.96	0.06	3.31	4.71	2.73
PEG$_{10}$PLA$_{10}$OVP$_{0.2}$	1.00	0.94	0.14	14.25	4.82	2.31	0.90	0.96	4.20	8.64	1.42	2.81	0.96	0.60	0.99	0.97	0.23	6.40	4.23	3.05
PEG$_{10}$PLA$_{10}$OVP$_{0.3}$	1.00	0.97	0.33	11.80	4.45	2.90	0.91	0.96	6.42	12.21	1.48	2.86	0.93	0.60	0.99	0.98	0.56	8.08	3.81	3.22

Table continued on next page

7-Ethyl-10-hydroxycamptothecin (SN38)

	First order						Higuchi						Korsmeyer-Peppas							
	R^2		MSE		MSC		R^2		MSE		MSC		n		R^2		MSE		MSC	
Sample pH	7.4	4.5	7.4	4.5	7.4	4.5	7.4	4.5	7.4	4.5	7.4	4.5	7.4	4.5	7.4	4.5	7.4	4.5	7.4	4.5
PEG$_{14}$PS$_{12}$	0.99	0.99	0.13	0.22	3.56	4.58	0.95	0.91	0.71	2.70	1.89	2.07	**0.82**	**0.83**	1.00	1.00	0.03	0.03	**4.80**	**6.50**
PEG$_{14}$PS$_{12}$ [b]	0.97		1.74		3.27		0.95		3.17		2.67		**0.72**		1.00		0.06		**6.52**	
PEG$_{14}$PS$_{12}$OVP$_{0.1}$	0.97	0.96	0.73	3.05	2.35	2.93	0.96	0.92	0.80	6.55	2.26	2.17	**0.72**	**0.74**	0.99	0.98	0.21	2.08	**3.48**	**3.26**
PEG$_{14}$PS$_{12}$OVP$_{0.2}$	0.98	0.97	0.59	9.58	2.91	2.95	0.96	0.94	1.32	16.45	2.10	2.41	**0.76**	**0.66**	0.99	0.97	0.18	8.79	**3.96**	**2.98**
PEG$_{14}$PS$_{12}$OVP$_{0.3}$	0.98	0.92	0.87	26.81	3.14	2.04	0.96	0.96	2.51	13.17	2.08	**2.75**	**0.77**	**0.53**	0.99	0.96	0.32	13.64	**4.01**	**2.64**
PEG$_{10}$PDL$_{10}$	0.99	0.99	0.23	0.22	3.30	4.21	0.95	0.92	0.89	1.79	1.95	2.11	**0.80**	**0.82**	1.00	1.00	0.07	0.03	**4.39**	**6.32**
PEG$_{10}$PDL$_{10}$ [b]	0.91		4.79		2.02		0.98		0.93		3.66		**0.61**		1.00		0.14		**5.52**	
PEG$_{10}$PDL$_{10}$OVP$_{0.1}$	0.98	0.98	0.56	1.72	2.87	3.50	0.95	0.94	1.29	5.29	2.04	2.38	**0.77**	**0.75**	0.99	0.99	0.22	0.59	**3.69**	**4.52**
PEG$_{10}$PDL$_{10}$OVP$_{0.2}$	0.98	0.99	0.97	1.34	2.95	**4.92**	0.95	0.90	2.76	26.64	1.90	1.93	**0.78**	**0.85**	0.99	0.99	0.69	2.91	**3.18**	4.08
PEG$_{10}$PDL$_{10}$OVP$_{0.3}$	0.98	0.99	1.74	4.41	2.81	**4.01**	0.96	0.94	3.06	21.19	2.25	2.44	**0.73**	**0.70**	0.99	0.98	0.64	7.28	**3.70**	3.45
PEG$_{10}$PLA$_{10}$	1.00	0.98	0.03	0.70	4.96	3.45	0.88	0.94	1.17	1.96	1.22	2.41	**1.07**	**0.76**	1.00	1.00	0.01	0.02	**6.46**	**7.07**
PEG$_{10}$PLA$_{10}$ [b]	0.99		1.00		3.92		0.92		5.19		2.27		**0.78**		0.99		0.40		**4.79**	
PEG$_{10}$PLA$_{10}$OVP$_{0.1}$	0.99	0.97	0.21	2.89	3.43	3.01	0.95	0.96	0.88	3.85	2.00	2.73	**0.80**	**0.69**	1.00	1.00	0.02	0.38	**5.50**	**4.99**
PEG$_{10}$PLA$_{10}$OVP$_{0.2}$	0.99	0.99	0.27	2.43	**4.09**	4.16	0.93	0.94	3.05	13.00	1.68	2.49	**0.87**	**0.72**	0.99	0.99	0.28	1.89	3.94	**4.36**
PEG$_{10}$PLA$_{10}$OVP$_{0.3}$	1.00	1.00	0.32	1.50	**4.39**	**4.99**	0.93	0.94	4.66	18.72	1.72	2.47	**0.86**	**0.72**	0.99	0.99	0.36	2.79	4.18	**4.31**

[a] All modelling was conducted using the DDSolver plugin for excel [5]. For each model, the coefficient of determination (R^2), mean square error (MSE) and model selection criterion (MSC) were calculated to determine the goodness-of-fit, and therefore, the most appropriate model for describing the release behaviour. An R^2 value closer to 1 indicates a closer correlation between the response values and the predicted response values. A MSE value closer to 0 indicates a closer correlation between the response values and the predicted response values. The MSC is a modified reciprocal form of the Akaike information criterion (AIC), which is normalized to be independent of the scaling of the data points. A larger MSC indicates a closer correlation between the response values and the predicted response values. The MSC was used to determine the best model fit to the release curves for each sample, as shown by the green color coding; alternative models that gave a MSC value within 0.1 unit of the best fit are shown with light green color coding. Light blue, dark blue and purple color coding for the release exponent corresponds to Fickian diffusion ($n < 0.45$), anomalous non-Fickian diffusion ($0.45<n<0.85$) and Case I/super case II transport ($n>0.85$) from a polymer matrix between cylindrical and spherical morphologies [6,7]. [b] Modelling was conducted across release curve regardless of pH.

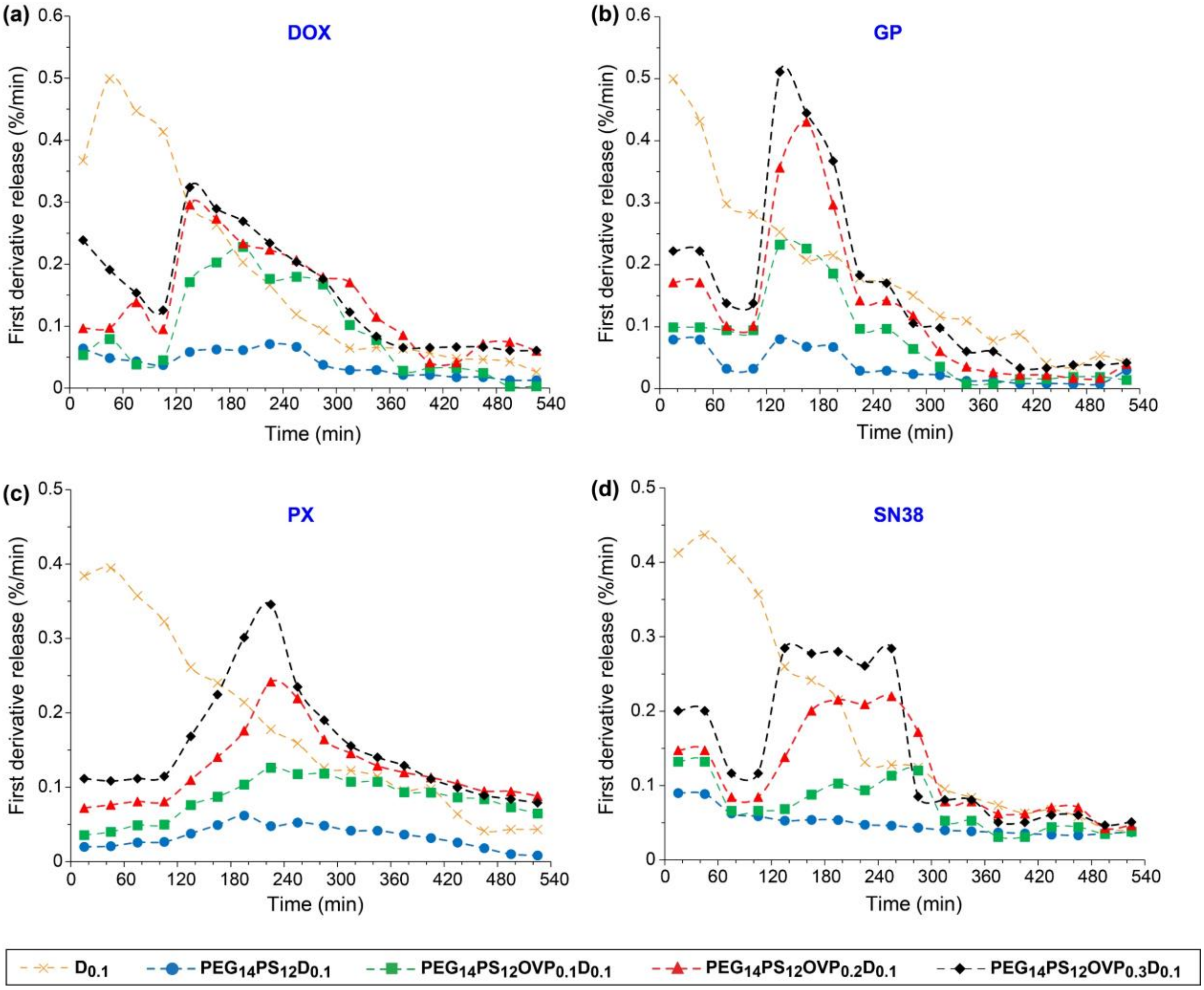

Figure S47: First derivative release curves for **(a)** DOX, **(b)** GP, **(c)** PX and **(d)** SN38 (all 0.1 mg/mg of copolymer) for free drug solutions and $PEG_{14}PS_{12}$ micelles with various OVP loadings (0, 0.1, 0.2 and 0.3 mg/mg of copolymer), calculated from *in vitro* release studies against PBS (10 mM, pH 7.4) for the first 2 h, followed by acetate buffer (10 mM, pH 4.5) for 7 h. Only symbols represent the experimental data; the dashed lines are guides to the eyes.

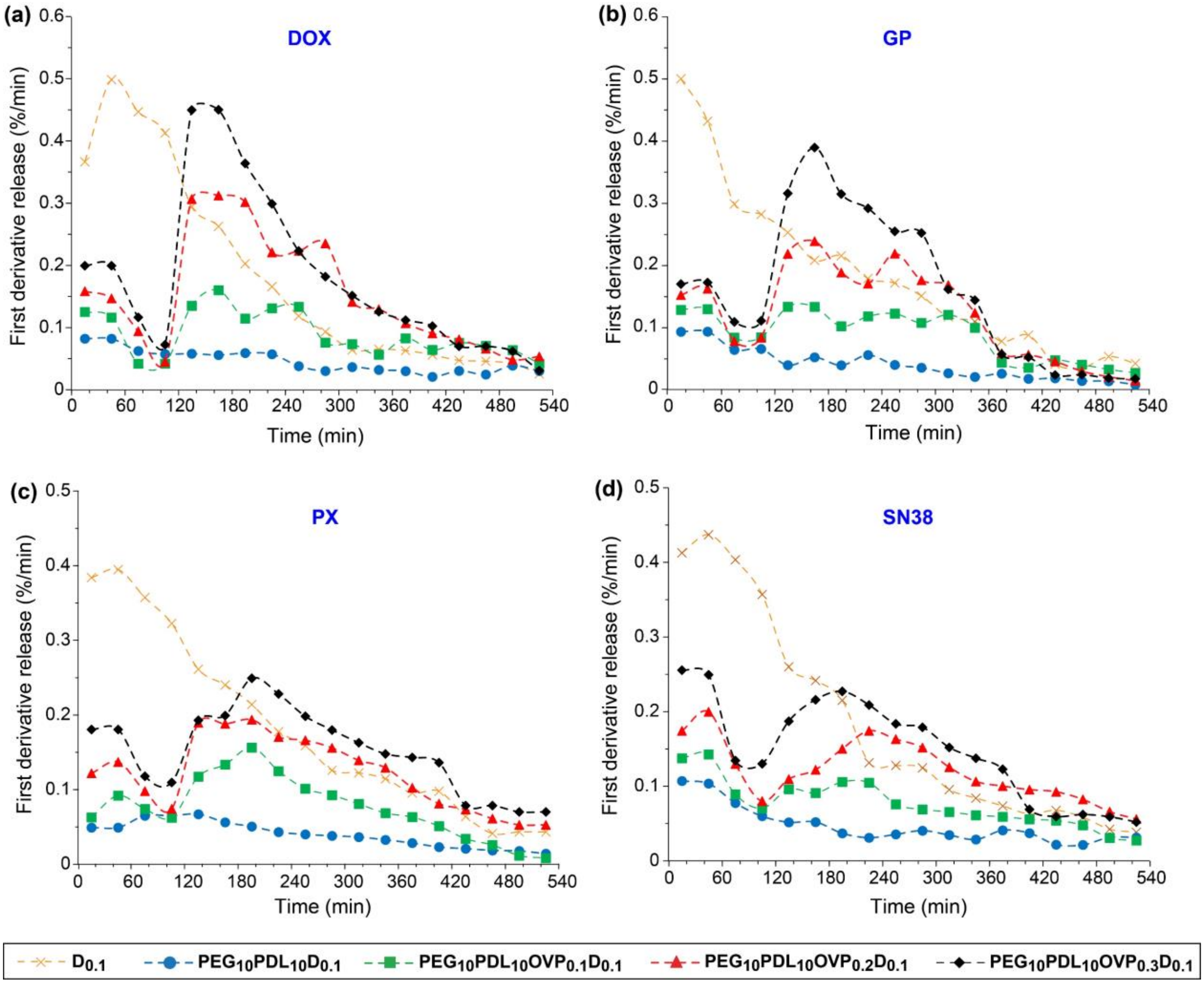

Figure S48: First derivative release curves for **(a)** DOX, **(b)** GP, **(c)** PX and **(d)** SN38 (all 0.1 mg/mg of copolymer) for free drug solutions and $PEG_{10}PDL_{10}$ micelles with various OVP loadings (0, 0.1, 0.2 and 0.3 mg/mg of copolymer), calculated from *in vitro* release studies against PBS (10 mM, pH 7.4) for the first 2 h, followed by acetate buffer (10 mM, pH 4.5) for 7 h. Only symbols represent the experimental data; the dashed lines are guides to the eyes.

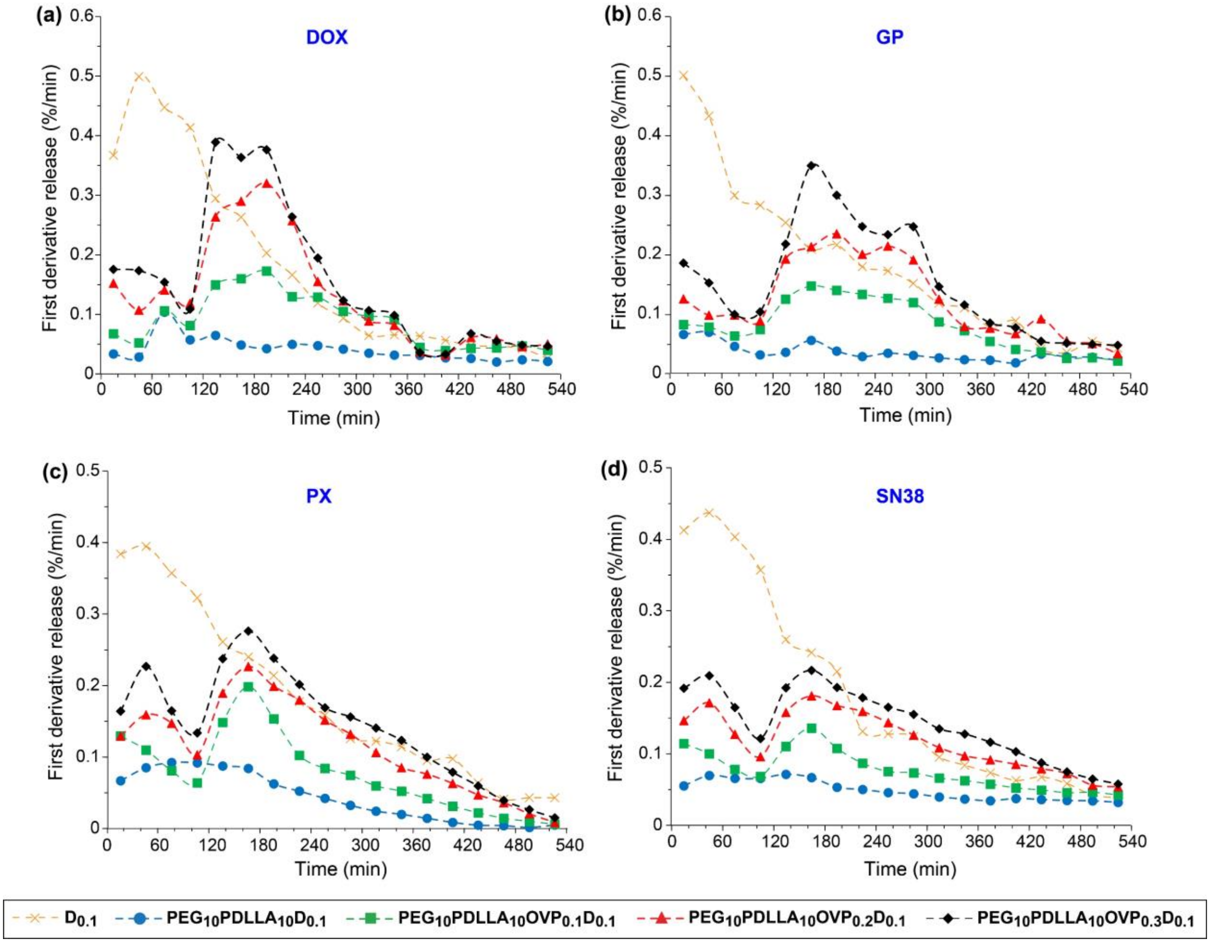

Figure S49: First derivative release curves for **(a)** DOX, **(b)** GP, **(c)** PX and **(d)** SN38 (all 0.1 mg/mg of copolymer) for free drug solutions and $PEG_{10}PLA_{10}$ micelles with various OVP loadings (0, 0.1, 0.2 and 0.3 mg/mg of copolymer), calculated from *in vitro* release studies against PBS (10 mM, pH 7.4) for the first 2 h, followed by acetate buffer (10 mM, pH 4.5) for 7 h. Only symbols represent the experimental data; the dashed lines are guides to the eyes.

Table S19: Drug loading and release from drug- and OVP-loaded micelles, for different drugs with a constant drug loading of 0.1 mg/mg of polymer.

Copolymer	Drug Loading (wt%)[a]				Total drug release at pH 4.5 (%)[b]			
	DOX	GP	PX	SN38	DOX	GP	PX	SN38
PEG_5PS_5	8.6 ± 0.1	8.8 ± 0.2	8.9 ± 0.1	1.7 ± 0.2	19.2 ± 1.2	16.9 ± 1.6	---	---
$PEG_5PS_5OVP_{0.1}$	8.4 ± 0.2	8.6 ± 0.1	8.6 ± 0.1	---	---	---	---	---
$PEG_5PS_5OVP_{0.2}$	7.4 ± 0.2	7.6 ± 0.5	7.4 ± 0.1	---	---	---	---	---
$PEG_5PS_5OVP_{0.3}$	6.6 ± 0.2	7.0 ± 0.2	6.7 ± 0.2	---	38.4 ± 2.9	35.2 ± 2.1	---	---
$PEG_{14}PS_{12}$	8.9 ± 0.1	9.0 ± 0.1	8.8 ± 0.1	8.5 ± 0.1	10.1 ± 2.3	13.3 ± 4.2	13.1 ± 2.2	19.2 ± 2.4
$PEG_{14}PS_{12}OVP_{0.1}$	8.8 ± 0.2	8.9 ± 0.1	8.8 ± 0.1	8.4 ± 0.1	37.7 ± 2.2	32.5 ± 4.4	36.1 ± 3.8	29.8 ± 2.4
$PEG_{14}PS_{12}OVP_{0.2}$	8.4 ± 0.1	8.5 ± 0.1	8.4 ± 0.1	7.6 ± 0.1	54.1 ± 3.7	57.4 ± 3.9	53.3 ± 4.5	61.1 ± 6.8
$PEG_{14}PS_{12}OVP_{0.3}$	8.2 ± 0.2	8.2 ± 0.1	7.6 ± 0.1	7.0 ± 0.1	66.4 ± 4.0	74.4 ± 4.2	69.2 ± 7.8	77.7 ± 9.6
$PEG_{10}PDL_{10}$	8.8 ± 0.1	9.0 ± 0.1	8.7 ± 0.1	8.6 ± 0.1	8.2 ± 2.3	12.3 ± 1.7	8.1 ± 2.5	15.8 ± 3.6
$PEG_{10}PDL_{10}OVP_{0.1}$	8.8 ± 0.1	8.9 ± 0.1	8.6 ± 0.1	8.1 ± 0.1	30.2 ± 2.2	35.7 ± 6.1	25.1 ± 4.2	32.1 ± 4.5
$PEG_{10}PDL_{10}OVP_{0.2}$	8.5 ± 0.1	8.7 ± 0.1	8.1 ± 0.3	7.6 ± 0.1	60.5 ± 3.4	54.6 ± 7.2	44.8 ± 3.6	58.3 ± 6.3
$PEG_{10}PDL_{10}OVP_{0.3}$	7.6 ± 0.1	7.8 ± 0.1	7.7 ± 0.1	6.7 ± 0.1	76.4 ± 4.0	82.5 ± 8.8	54.4 ± 4.5	80.4 ± 6.8
$PEG_{10}PLA_{10}$	8.7 ± 0.1	8.8 ± 0.1	8.8 ± 0.1	8.3 ± 0.2	9.1 ± 2.2	12.9 ± 2.3	3.4 ± 2.5	12.1 ± 1.9
$PEG_{10}PLA_{10}OVP_{0.1}$	8.5 ± 0.1	8.7 ± 0.3	8.7 ± 0.2	8.2 ± 0.2	31.8 ± 2.0	35.8 ± 2.5	19.4 ± 3.9	22.1 ± 3.3
$PEG_{10}PLA_{10}OVP_{0.2}$	8.2 ± 0.2	8.3 ± 0.1	8.1 ± 0.1	7.6 ± 0.1	45.1 ± 5.4	60.2 ± 3.8	33.7 ± 4.0	37.6 ± 5.7
$PEG_{10}PLA_{10}OVP_{0.3}$	7.5 ± 0.2	7.7 ± 0.3	7.4 ± 0.2	6.6 ± 0.1	59.1 ± 6.2	79.7 ± 5.1	43.9 ± 6.4	50.4 ± 6.3

[a] Drug loading calculated from measured EE%. [b] Total drug release at pH 4.5 over 7 h excluding any release observed at pH 7.4.

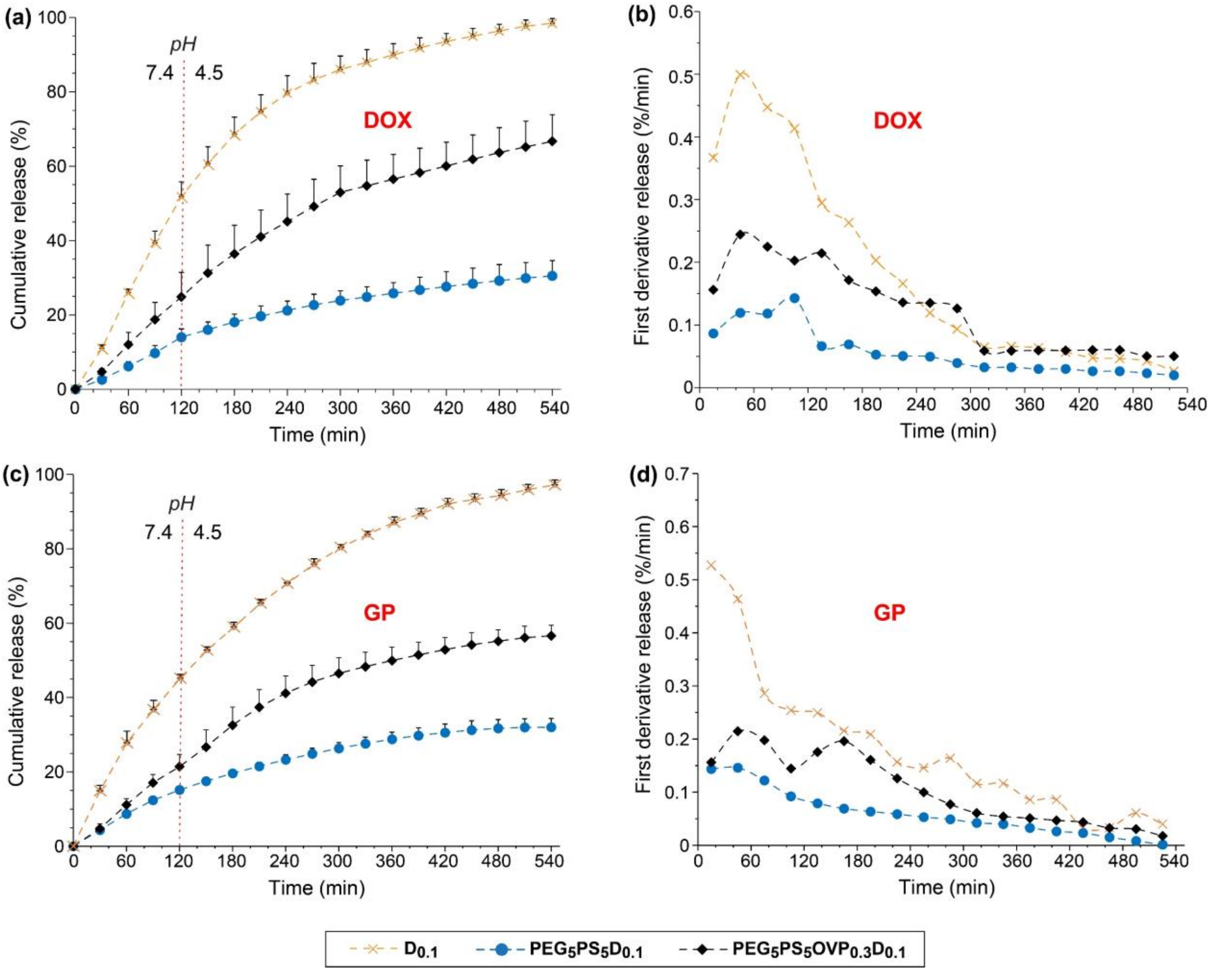

Figure S50: *In vitro* **(a,c)** DOX and GP (0.1 mg/mg of copolymer) release profiles, respectively, and **(b,d)** first derivative release curves, for free DOX/GP, and $PEG_5PS_5DOX_{0.1}$/$PEG_5PS_5GP_{0.1}$ and $PEG_5PS_5OVP_{0.3}DOX_{0.1}$/$PEG_5PS_5OVP_{0.3}GP_{0.1}$ micelles in PBS (pH 7.4) and acetate buffer (pH 4.5) with or without 0.5% tween 20 (in case of GP) at ambient temperature. Data are shown as the average cumulative release (%) + SD. Only symbols represent the experimental data; the dashed lines are guides to the eyes.

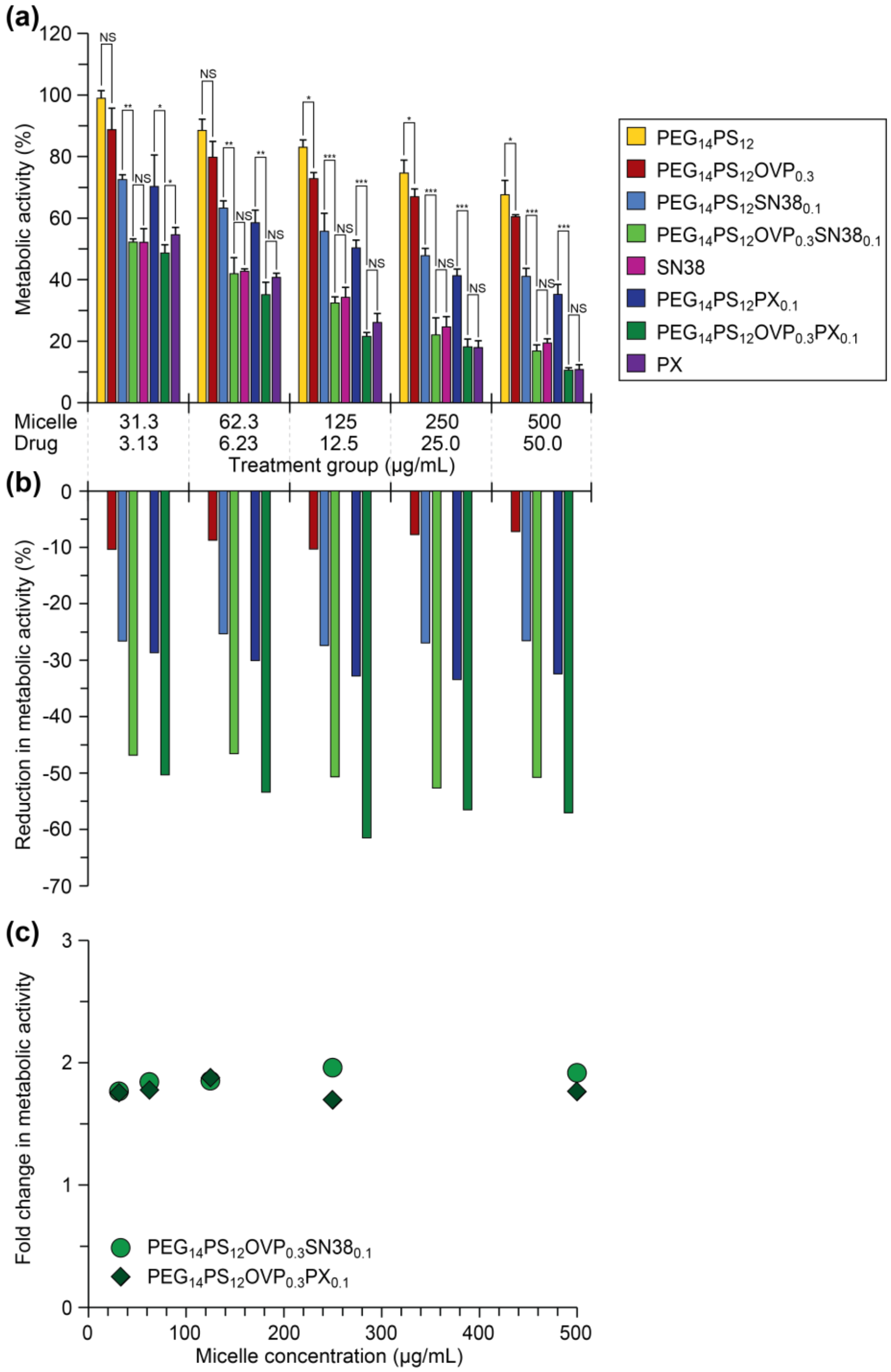

Figure S51: **(a)** *In vitro* percentage metabolic activity of SKOV-3 cells relative to negative controls (untreated cells) when treated with $PEG_{14}PS_{12}$ micelles formulations and free drugs at various doses, following incubation for 48 h; treatment group concentrations refer to the micelle (31.3-500 μg/mL) and drug concentrations (3.13-50.0 μg/mL) if present. Data are shown as the mean metabolic activity (%) ± SD (n = 3); NS, *, ** and *** represent not significant ($p \geq 0.05$), $p < 0.05$, $p < 0.01$ and $p < 0.001$, respectively. **(b)** Percentage reduction in metabolic activity of OVP- and drug-loaded micelles relative to blank $PEG_{14}PS_{12}$ micelles. **(c)** Fold change in reduction of metabolic activity of drug-loaded $PEG_{14}PS_{12}OVP_z$ micelles relative to drug-loaded $PEG_{14}PS_{12}$ micelles following subtraction of any reduction associated with OVP.

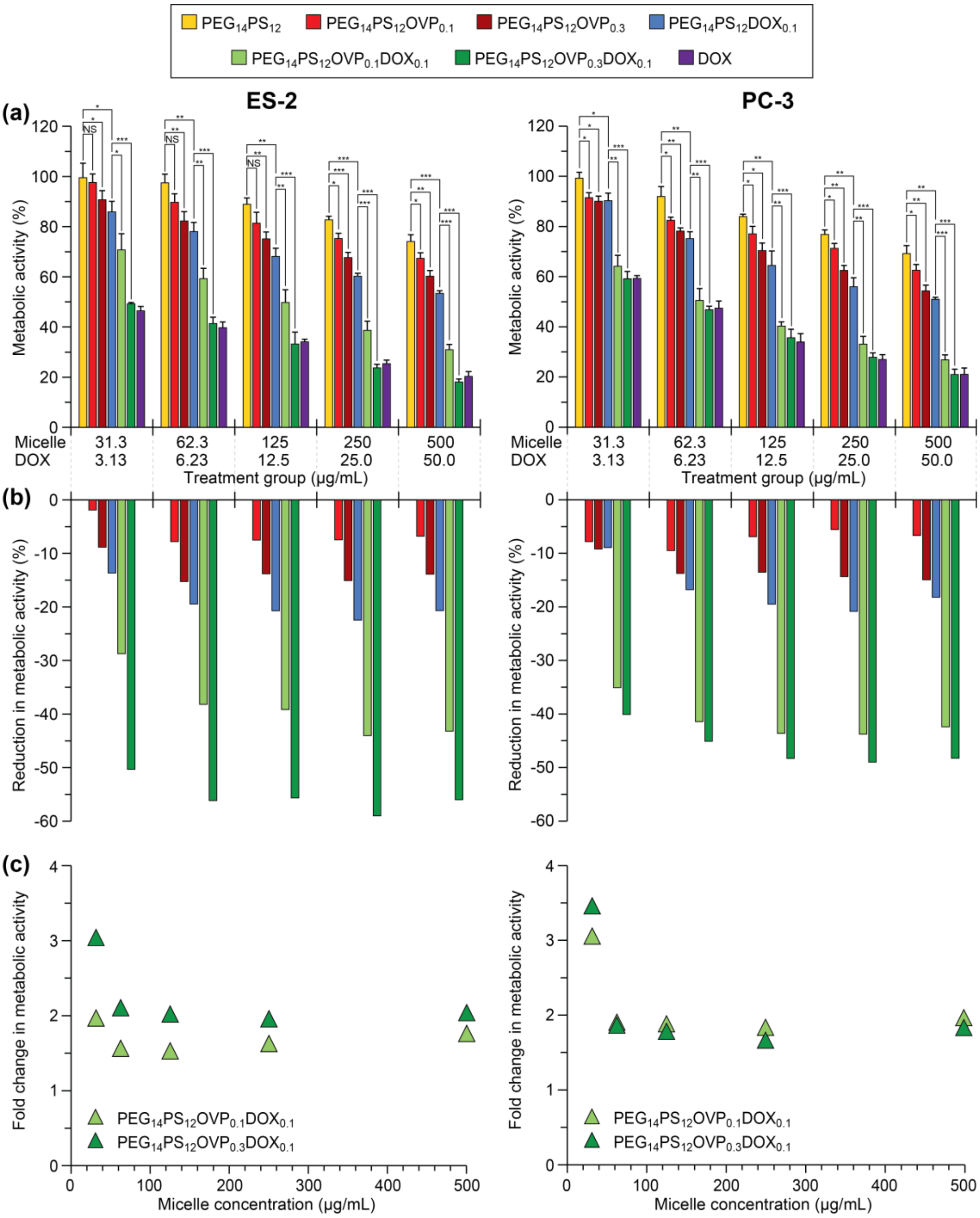

Figure S52: **(a)** *In vitro* percentage metabolic activity of ES-2 (left) and PC-3 cells (right) relative to negative controls (untreated cells) when treated with $PEG_{14}PS_{12}$ micelles formulations and free DOX at various doses, following incubation for 48 h; treatment group concentrations refer to the micelle (31.3-500 µg/mL) and DOX concentrations (3.13-50.0 µg/mL) if present. Data are shown as the mean metabolic activity (%) ± SD (n = 3); NS, *, ** and *** represent not significant ($p \geq 0.05$), $p < 0.05$, $p < 0.01$ and $p < 0.001$, respectively. **(b)** Percentage reduction in metabolic activity of OVP- and DOX-loaded micelles relative to blank $PEG_{14}PS_{12}$ micelles. **(c)** Fold change in reduction of metabolic activity of DOX-loaded $PEG_{14}PS_{12}OVP_z$ micelles relative to drug-loaded $PEG_{14}PS_{12}$ micelles following subtraction of any reduction associated with OVP.

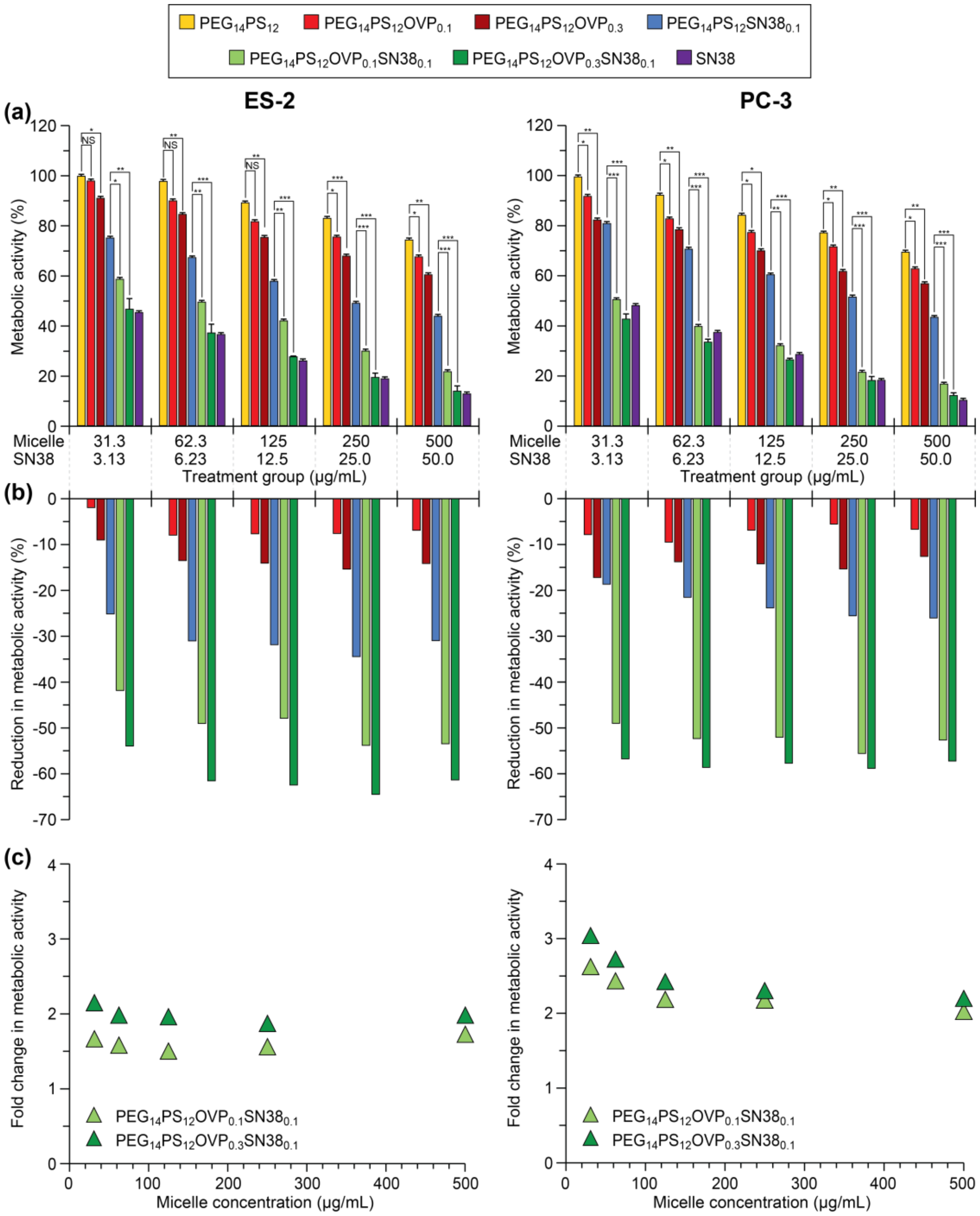

Figure S53: **(a)** *In vitro* percentage metabolic activity of ES-2 (left) and PC-3 cells (right) relative to negative controls (untreated cells) when treated with PEG$_{14}$PS$_{12}$ micelles formulations and free SN38 at various doses, following incubation for 48 h; treatment group concentrations refer to the micelle (31.3–500 μg/mL) and SN38 concentrations (3.13–50.0 μg/mL) if present. Data are shown as the mean metabolic activity (%) ± SD (n = 3); NS, *, ** and *** represent not significant ($p \geq 0.05$), $p < 0.05$, $p < 0.01$ and $p < 0.001$, respectively. **(b)** Percentage reduction in metabolic activity of OVP- and SN38-loaded micelles relative to blank PEG$_{14}$PS$_{12}$ micelles. **(c)** Fold change in reduction of metabolic activity of SN38-loaded PEG$_{14}$PS$_{12}$OVP$_{z}$ micelles relative to drug-loaded PEG$_{14}$PS$_{12}$ micelles following subtraction of any reduction associated with OVP.

References

1. Hussain, M.S.; Khetan, R.; Albrecht, H.; Krasowska, M.; Blencowe, A. Oligoelectrolyte-mediated, pH-triggered release of hydrophobic drugs from non-responsive micelles: Influence of oligo (2-vinyl pyridine)-loading on drug-loading, release and cytotoxicity. *Int. J. Pharm.* **2024**, *661*, 124368.

2. Hussain, M.S.; Khetan, R.; Clulow, A.J.; Ganesan, R.; MacMillan, A.; Robinson, N.; Ahmed-Cox, A.; Krasowska, M.; Al-brecht, H.; Blencowe, A.. Teaching an Old Dog New Tricks: A Global Approach to Enhancing the Cytotoxicity of Drug-Loaded, Non-responsive Micelles Using Oligoelectrolytes. *ACS Appl. Mater. Interfaces* **2024**, *16*, 9736-9748.

3. Sanna, V.; Roggio, A.M.; Posadino, A.M.; Cossu, A.; Marceddu, S.; Mariani, A.; Alzari, V.; Uzzau, S.; Pintus, G.; Sechi, M. Novel docetaxel-loaded nanoparticles based on poly(lactide-co-caprolactone) and poly(lactide-co-glycolide-co-caprolactone) for prostate cancer treatment: formulation, characterization, and cytotoxicity studies. *Nanoscale Res. Lett.* **2011**, *6*, 260.

4. Martuscelli, E.; Silvestre, C.; Addonizio, M.L.; Amelino, L. Phase structure and compatibility studies in poly (ethylene oxide)/poly (methyl methacrylate) blends. *Macromol. Chem. Phys.* **1986**, *187*, 1557-1571.

5. Zhang, Y.; Huo, M.; Zhou, J.; Zou, A.; Li, W.; Yao, C.; Xie, S. DDSolver: an add-in program for modeling and comparison of drug dissolution profiles. *AAPS J.* **2010**, *12*, 263-271.

6. Kosmidis, K.; Argyrakis, P.; Macheras, P. Fractal kinetics in drug release from finite fractal matrices. *J. Chem. Phys.* **2003**, *119*, 6373-6377.

7. Fosca, M.; Rau, J.V.; Uskoković, V. Factors influencing the drug release from calcium phosphate cements. *Bioact. Mater.* **2022**, *7*, 341-363.
